# Internal Dynamics of Pyrene-Labeled Polyols Studied Through the Lens of Pyrene Excimer Formation

**DOI:** 10.3390/polym17141979

**Published:** 2025-07-18

**Authors:** Franklin Frasca, Jean Duhamel

**Affiliations:** Institute for Polymer Research, Waterloo Institute for Nanotechnology, Department of Chemistry, University of Waterloo, 200 University Avenue West, Waterloo, ON N2L 3G1, Canada; fofrasca@uwaterloo.ca

**Keywords:** pyrene excimer formation (PEF), model-free analysis (MFA), local pyrene concentration ([*Py*]_loc_), internal dynamics, multifunctional oligomers

## Abstract

Series of pyrene-labeled diols (Py_2_-DOs) and polyols (Py-POs) were synthesized by coupling a number (*n*_PyBA_) of 1-pyrenebutyric acids to diols and polyols to yield series of end-labeled linear (*n*_PyBA_ = 2) and branched (*n*_PyBA_ > 2) oligomers, respectively. Pyrene excimer formation (PEF) between an excited and a ground-state pyrene was studied for the Py_2_-DO and Py-PO samples by analyzing their fluorescence spectra and decays in tetrahydrofuran, dioxane, *N*,*N*-dimethylformamide, and dimethyl sulfoxide. Global model-free analysis (MFA) of the pyrene monomer and excimer fluorescence decays yielded the average rate constant (<*k*>) for PEF. After the calculation of the local pyrene concentration ([*Py*]_loc_) for the Py_2_-DO and Py-PO samples, the <*k*>-vs.-[*Py*]_loc_ plots were linear in each solvent, with larger and smaller slopes for the Py_2_-DO and Py-PO samples, respectively, resulting in a clear kink in the middle of the plot. The difference in slope was attributed to a bias for PEF between pyrenes close to one another on the densely branched Py-PO constructs resulting in lower apparent [*Py*]_loc_ and <*k*> values. This study illustrated the ability of PEF to probe how steric hindrance along a main chain affects the dynamic encounters between substituents in multifunctional oligomers such as diols and polyols.

## 1. Introduction

The average end-to-end distance (*r*_EE_) is a useful mathematical parameter in macromolecular science for developing theoretical models describing macromolecular conformations [1,2,3]. The appeal of *r*_EE_ stems from its ability to simplify the minute chemical details involved in the wide variety of structural units that can constitute any given macromolecule while capturing the main features describing macromolecular conformations with a single parameter. Despite its mathematical elegance, it is usually not *r*_EE_ that is used in experimental and theoretical studies to characterize macromolecular conformations but rather the radius of gyration (*R*_G_) obtained from scattering experiments [4,5,6]. The main reason for the overwhelming use of *R*_G_ over *r*_EE_ arises from the ability to extract *R*_G_ from the scattering profile of any macromolecule by applying Guinier’s approximation at low scattering angles [7] and the absence of a similarly straightforward procedure for determining *r*_EE_ experimentally. Furthermore, the *r*_EE_ parameter is more challenging to apply for multi-ended macromolecules like dendrimers and polymeric bottlebrushes.

Theoretically, labeling the multiple ends of a macromolecule with fluorophores having a well-characterized ability to communicate photochemically with each other over a distance should provide a straightforward experimental means to determine *r*_EE_. In practice, however, the realization that every chain length spanning two fluorophores yields a different rate constant (*k*_i_) for any given distance-dependent photochemical process implies that any macromolecule labeled with more than two fluorophores generates a multiexponential decay, with each decay time (*τ*_i_) being associated with one of the rate constants *k*_i_ [8]. Unfortunately, the impossibility of extracting all *τ*_i_ values from the analysis of a multiexponential fluorescence decay with a sum of exponentials [9,10,11,12,13,14] prevents the quantitative determination of the distribution of rate constants *k*_i_ and thus the associated recovery of *r*_EE_. This situation rationalizes why, to this day, the quantitative study of any macromolecule by fluorescence involves the attachment at two specific positions in the macromolecule of two fluorophores capable of communicating photochemically, typically to the two ends of a monodisperse linear oligomer [15,16]. This experimental design ensures that the photophysical process undergone by the two fluorophores can be handled by a single rate constant *k*_1_ associated with a single decay time *τ*_1_ that can be easily extracted from the analysis of the corresponding monoexponential fluorescence decay. Unfortunately, this modus operandi is also extremely restrictive and prevents the characterization by fluorescence of the many macromolecules having architectures more complex than those of linear chains, such as dendrimers or polymeric bottlebrushes (PBBs). In fact, these multi-ended macromolecules with numerous reactive terminals lend themselves naturally to multiple labeling, which would result in a broad distribution of rate constants, thus preventing the determination of *r*_EE_.

A solution to this problem was proposed in a recent report involving the study of pyrene-labeled macromolecules (PyLMs) [17]. PEF has been harnessed in many applications, such as for the detection of hybridization between two complementary oligonucleotides [18,19,20,21,22,23] or the detection of metal cations [24,25,26,27,28]. However, most of these applications use PEF in a qualitative manner, focusing on the ratio of the fluorescence intensity of the pyrene excimer over that of the pyrene monomer. A much more quantitative analysis of PEF was achieved by noting that the average decay time (<*τ*>) for PEF was recovered more accurately than the individual pre-exponential factors (*a*_i_) and *τ*_i_ values retrieved from the fit of multiexponential fluorescence decays with sums of exponentials [29]. <*τ*> was used to calculate the average rate constant (<*k*>) for PEF between the excited pyrene and the multiple ground-state pyrenyl labels of a PyLM. Furthermore, <*k*> given in Equation (1) was found to be directly proportional to the local concentration ([*Py*]_loc_) of ground-state pyrenes in the PyLM, with the proportionality constant *k*_diff_ being the bimolecular rate constant for PEF by diffusive encounters. In Equation (1), *τ*_M_ is the natural lifetime of the pyrenyl label. By definition, [*Py*]_loc_ is equal to the number (*n*_Py_) of ground-state pyrenes inside the volume (= *A* × *L*_Py_^3^) probed by a pyrenyl label, while it remains excited, divided by that volume. *A* in Equation (1) is a geometric factor used to represent the assumed shape of the macromolecule, for example, a sphere, for which *A* equals *π*/6, and *L*_Py_ represents the average distance separating every two pyrenyl labels. Consequently, Equation (1) provides a direct link between the experimentally determined and theoretically predicted parameters <*k*> and *L*_Py_, respectively. If the pyrenyl labels are covalently attached to the ends of a macromolecule, *L*_Py_ provides a measure of *r*_EE_ after accounting for the few non-hydrogen atoms used to link the pyrenyl moieties to the macromolecule. Through the comparison of <*k*> and *L*_Py_, *L*_Py_ enables the validation of models developed to describe the conformation of macromolecules, which can then be applied to determine *r*_EE_, as was carried out for series of pyrene end-labeled PBBs [30] and dendrimers [31,32].

(1) $$<k>\;=\;\frac{\sum_{i}a_{i}}{\sum_{i}a_{i}\tau_{i}}\;-\;\frac{1}{\tau_{M}}\;=\;k_{diff}\times {\left[Py\right]}_{loc}\;=\;k_{diff}\times \frac{n_{Py}}{A\times L_{Py}^{3}}\;∼\;\frac{I_{E}}{I_{M}}$$

To date, the validity of Equation (1) has been demonstrated through the study of 55 PyLMs representing three different families of macromolecules, namely pyrene end-labeled linear monodisperse oligomers, polydisperse polymers randomly labeled with pyrene, and monodisperse dendrimers end-labeled with pyrene [17]. The versatility of the methodology developed with Equation (1) is illustrated in the present study aiming to characterize the conformation of a novel family of PyLMs, namely series of linear diols and polyols labeled with 1-pyrenebutyric acid to yield Py_2_-DO and Py-PO constructs, respectively, whose architecture differs from the three types of PyLMs already studied [17]. These oligomers are too small to provide any meaningful scattering signal, which prevents their conformational characterization by scattering techniques. In contrast, PEF provides a unique experimental means to probe both the conformation and internal dynamics of these complex pyrene-labeled multifunctional oligomers.

## 2. Materials and Methods

### 2.1. Chemicals

Tetrahydrofuran (THF, ≥99.0%, distilled in glass) was supplied by Honeywell Riedel-de Haën, Seelze, Germany. Ethanol (EtOH, anhydrous) was obtained from Greenfield Global, Brampton, ON, Canada. Sodium sulfate (Na_2_SO_4_, anhydrous) and glycerol (Glycerol, ACS grade, ≥99.5%) were purchased from EMD Millipore Corp., Burlington, MA, USA. *N*-Hydroxybenzotriazole monohydrate (HOBt·H_2_O, >98%) was supplied by Creosalus, Louisville, KY, USA. Acetone (HPLC grade), acetonitrile (ACN, HPLC grade, ≥99.9%), methanol (MeOH, HPLC grade), hexanes (98.5%), ethyl acetate (HPLC grade, 99.7%), deuterated chloroform (CDCl_3_, 99.8%), dichloromethane (DCM, HPLC grade, 99.8%), chloroform (CHCl_3_, HPLC grade, 99.8%), hydrochloric acid (HCl, 12.2 M), sodium chloride (NaCl, ≥99.5%), sodium hydroxide pellets (ACS grade, 97.0%), toluene (HPLC grade, 99.9%), *N*,*N*-dimethylformamide (DMF, ACS grade, 99.8%), dimethyl sulfoxide (DMSO, ACS, 99.9%), 1,4-dioxane (dioxane, ACS grade, 99.0%), *N*-ethyl-*N*′-(3-dimethylaminopropyl)carbodiimide hydrochloride (EDC·HCl, crystalline), 1-pyrenebutyric acid (PyBA, 97%), 4-dimethylaminopyridine (DMAP, 99%), *N*,*N*-diisopropylethylamine (DIPEA, >99.5%, Biotech. Grade), 1,16-hexadecanediol (HexadecDiol, 98%), 1,10-decanediol (DecDiol, 98%), 1,6-hexanediol (HexDiol, 99%), 1,4-butanediol (ButDiol, 99%), ethylene glycol (EG, anhydrous, 99.8%), *meso*-erythritol (Erythritol, ≥99%), adonitol (Adonitol, ≥99%), and D-sorbitol (Sorbitol, 99%) were obtained from Sigma-Aldrich, Markham, ON, Canada. All chemicals were used as received unless otherwise stated.

### 2.2. Instrumentation

#### 2.2.1. Nuclear Magnetic Resonance (NMR)

^1^H NMR and COSY spectra of the Py_2_-DO and Py-PO samples were acquired on a 300 MHz NMR instrument from Brucker (Billerica, MA, USA) in CDCl_3_ with sample concentrations ranging from 1 to 10 mg/mL depending on sample availability after purification. They are provided in Figures S1–S22 as Supplementary Materials.

#### 2.2.2. Mass Spectrometry (MS)

A Q-Exactive Orbitrap mass spectrometer from Thermo Fisher Scientific (Mississauga, ON, Canada) equipped with a high-resolution hybrid quadrupole orbitrap using an ESI ion source in positive mode with lock mass correction was used for high-resolution mass spectrometry. All accurate masses reported were within 4 ppm of the calculated masses for the given compound or adduct. Low-resolution mass spectra and resultant tandem mass spectroscopy fragmentation data were acquired on a Thermo Fisher Scientific LTQ-XL instrument equipped with a low-resolution linear ion trap. Samples were prepared in acetonitrile at concentrations of 1–10 μM before the addition of formic acid (0.1 vol%) for ionization prior to infusion at 10–20 μL/min with either system. Mass spectra are provided in Figures S23–S38 as Supplementary Materials.

#### 2.2.3. UV–Visible Spectrophotometry (UV-Vis)

Quartz cells with a 1.0 cm pathlength were used in a Cary 100 UV–visible spectrophotometer (Varian, Palo Alto, CA, USA). All absorption spectra were acquired from 200 to 400 nm and blanked against the solvent used (THF, DMF, DMSO, or dioxane).

#### 2.2.4. Steady-State Fluorescence (SSF)

A HORIBA QM-400 spectrofluorometer (HORIBA, London, ON, Canada) with a xenon arc lamp was used to obtain the fluorescence spectra. All solutions were degassed with a gentle flow of nitrogen to remove dissolved oxygen and prevent unwanted quenching of pyrene fluorescence. The solutions were prepared with a 2.5 μM pyrene concentration for the fluorescence spectra, which were acquired with the right-angle geometry.

#### 2.2.5. Time-Resolved Fluorescence (TRF)

Fluorescence decays were obtained with an IBH (IBH, Glasgow, UK) or a HORIBA Ultima Ultrafast time-resolved fluorometer (HORIBA, Piscataway, NJ, USA) with a 340 nm nano-LED or a 336 nm delta diode laser as the light source, respectively. Acquisition of the monomer and excimer fluorescence decays was conducted at 375 and 510 nm with 370 and 495 nm long-pass cutoff filters, respectively, to shield the detector from residual stray light. To outgas oxygen, which is a powerful quencher of pyrene, nitrogen was gently bubbled through all the samples for at least 20 min for solutions in THF and for at least 40 min for the more viscous solutions in DMF, DMSO, and dioxane. Decays were acquired with 20,000 counts at the decay maximum using the same right-angle geometry used in the SSF measurements.

### 2.3. Fluorescence Decay Analysis

Model-free analysis (MFA) was applied for the global fitting of the monomer and excimer fluorescence decays of the Py-PO constructs with Equations (S1) and (S2) in the Supplementary Materials. The four pyrene species *Py*_diff_*, *Py*_free_*, *E*0*, and D* have been found to describe PEF between the pyrene moieties covalently attached onto a macromolecule. *Py*_free_* represents the pyrenyl labels that are isolated, cannot form an excimer, and emit as if they were free in solution with their lifetime *τ*_M_. *Py*_diff_* describes those pyrenyl labels that form an excimer by diffusive encounters with a ground-state pyrene. *E*0* and D* represent those pyrene species involved in pyrene aggregates that form an excimer instantaneously upon direct excitation of a pyrene aggregate, resulting in a complex where the pyrenyl labels are properly or poorly stacked, resulting in an excimer emitting with a short (*τ*_E0_) or long (*τ*_D_) lifetime, respectively. The amounts of each pyrenyl species *Py*_free_*, *Py*_diff_*, *E*0*, and D* are quantified by their molar fractions *f*_free_, *f*_diff_, *f*_E0_, and *f*_D_, respectively, which are retrieved from the global MFA of the fluorescence decays with Equations (S3)–(S9) in the Supplementary Materials. The quality of the fits obtained with the MFA was assessed by ensuring that a χ^2^ value smaller than 1.3 was obtained and that both the residuals and autocorrelation of the residuals were randomly distributed around 0. The parameters obtained through the global MFA of the decays of the Py-PO samples are listed in Tables S1–S3 in the Supplementary Materials. These parameters include the fluorescence decay times *τ*_i_ and their preexponential factors *a*_i_ used to calculate both <*k*> according to Equation (1) and the molar fractions *f*_free_, *f*_diff_, and *f*_agg_ (= *f*_E0_ + *f*_D_).

### 2.4. Synthesis of the Pyrene-Labeled Diols (Py_2_-DOs) and Polyols (Py-POs)

The synthesis of the Py_2_-DO and Py-PO samples was achieved with carbodiimide coupling to append 1-pyrenebutyric acid to the diols or polyols of interest through an ester linkage. The Py_2_-DO and Py-PO samples were isolated via column chromatography with silica gel. The syntheses are described in further detail as Supplementary Materials, where their ^1^H NMR and COSY spectra, accurate masses obtained via high-resolution MS (HRMS), and a brief discussion of their fragmentation patterns from tandem mass spectrometry (MS^n^) experiments are provided.

## 3. Results and Discussion

The direct relationship that exists between <*k*> and [*Py*]_loc_ has been established for several macromolecules that include polydisperse long linear chains randomly labeled with pyrene and pyrene end-labeled monodisperse short chains, dendrimers, and polymeric bottlebrushes [17,29,30,31,32]. The present work departs from these earlier studies as it aims to characterize the PEF response not of pyrene labeled macromolecules (PyLMs) but instead of multifunctional linear or branched oligomers that enable the introduction of pyrene labels at the ends or the branching points of these oligomers. Deviation from the <*k*>-vs.-[*Py*]_loc_ relationship established for PyLMs is expected to provide information on how the internal dynamics of these multifunctional oligomers are affected by their linear or branched architecture.

### 3.1. Preparation of the Py_2_-DO and Py-PO Samples

**Figure 1:**
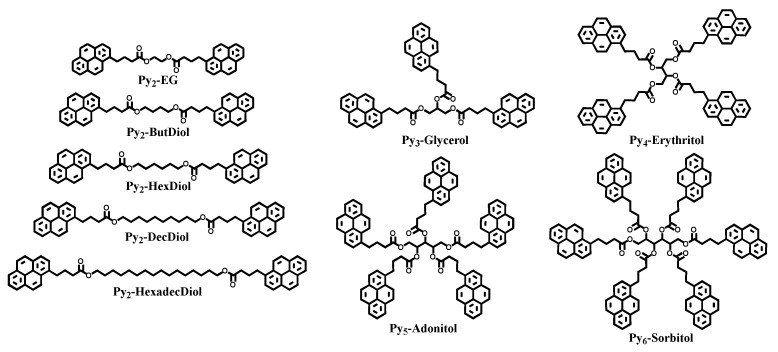
Chemical structures of the synthesized (**left**) Py_2_-DO and (**right**) Py-PO samples.

To investigate how the <*k*>-vs.-[*Py*]_loc_ relationship holds with pyrene-labeled oligomers, series of *linear* diols (ethylene glycol, 1,4-butanediol, 1,6-hexanediol, 1,10-decanediol, and 1,16-hexadecanediol) and *branched* polyols (glycerol, erythritol, adonitol, and sorbitol) were labeled with 1-pyrenebutyric acid (PyBA) before subsequent isolation and characterization to obtain the series of Py_2_-DO and Py-PO constructs shown in Figure 1. More details about the synthesis of the Py_2_-DO and Py-PO samples are provided in the Supplementary Materials. Characterization via ^1^H NMR and COSY spectra (see Figures S4–S22 in the Supplementary Materials) and accurate mass determination via HRMS were performed to ensure that the diols and polyols were fully labeled with PyBA, along with MSMS fragmentation, which showed the successive fragmentation of pyrenes from the fully labeled *branched* Py-PO samples.

### 3.2. Calculation of [Py]_loc_ for the Py_2_-DO and Py-PO Samples

The [*Py*]_loc_ values were calculated according to Equation (2), where *n*_Py_ is the number of ground-state pyrenes in the oligomer after the excitation of one of the pyrenyl labels (i.e., *n*_Py_ equals the total number of pyrenyl labels in the pyrene-labeled oligomer minus 1), *L*_Py_ is the root-mean-squared end-to-end distance between the pyrenyl labels of the Py_2_-DO or Py-PO samples, *l* is a bond length, and *A* is a geometrical factor that would equal *π*/6 if the macromolecular volume were approximated as a sphere. Since *A* is a mere constant, [*Py*]_loc_ and *n*_Py_/(*L*_Py_/*l*)^3^ are used interchangeably from this point on.

(2) $${\left[Py\right]}_{loc}\;=\;\frac{n_{Py}}{(A\times l^{3})\times {\left(L_{Py}/l\right)}^{3}}$$

**Figure 2:**
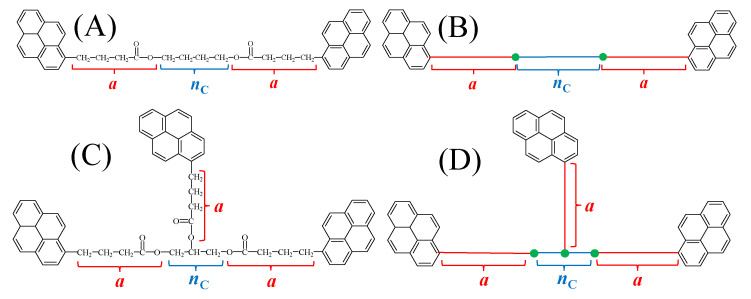
Illustration of the molecular parametrization conducted for the (**A**) Py_2_-DO and (**C**) Py-PO samples and their equivalent schematic representations shown in (**B**) and (**D**) used for *L*_Py_ calculations. (red) linker made of *a* atoms connecting pyrene to the oligomer, (blue) number of *n*_C_ carbon atoms in the linear part of the construct, (green dots) attachment points of the pyrene derivative.

The calculation of *L*_Py_ required that each pyrene-labeled oligomer be molecularly parametrized as shown in Figure 2. The chemical structures of the Py_2_-DO and Py-PO samples were parametrized by using the number (*a* = 5) of non-hydrogen atoms in the linker connecting the pyrenyl moiety to one of the carbon atoms in the diols or polyols. In these derivations, the molecular segments connecting every two pyrenyl groups were assumed to obey Gaussian statistics, an assumption which has proved highly valuable to determine (*L*_Py_/*l*)^2^ for numerous pyrene-labeled macromolecules [17,29,30,31,32]. Using this assumption, a diol made of an alkyl chain with *n*_C_ methylenes would yield the expression for (*L*_Py_/*l*)^2^ given by Equation (3).

For a branched Py-PO made of a polyol with *n*_C_ carbons, the total number of end-to-end distances between every two pyrenyl end groups equals *n*_C_ × (*n*_C_−1). Summing all the end-to-end distances between every two pyrenyl groups yields the numerator of Equation (4), which needs to be normalized by the total number of end-to-end distances to yield (*L*_Py_/*l*)^2^. Equation (4) was then simplified into Equation (5). Equation (5) was used to calculate (*L*_Py_/*l*)^2^ for the branched polyols. In both equations, *l* represents the average bond length between two non-hydrogen atoms involved in a segment connecting two pyrenyl moieties.

(3) $${\left(\frac{L_{Py}}{l}\right)}^{2}\;=\;2a\;+\;n_{C}\;\mathrm{for}\;a\;\mathrm{linear}\;{\mathrm{Py}}_{2}\;\mathrm{-DO}\;\mathrm{made}\;\mathrm{of}\;n_{c}\;\mathrm{methylenes}$$

(4) $${\left(\frac{L_{Py}}{l}\right)}^{2}\;=\;\frac{2\times \left(\left(n_{C}-1)\times (2a+1+1)+(n_{C}-2)\times (2a+1+2)+\ldots +1\times (2a+1+(n_{C}-1\right)\right)}{n_{C}(n_{C}-1)}$$

(5) $${\left(\frac{L_{Py}}{l}\right)}^{2}=\;2a\;+\;1\;+\;\frac{n_{C}\;+\;1}{3}\;\mathrm{for}\;a\;\mathrm{branched}\;\mathrm{Py-PO}\;\mathrm{made}\;\mathrm{of}\;n_{c}\;\mathrm{carbons}$$

The *n*_Py_/(*L*_Py_/*l*)^3^ values calculated with Equations (2)–(5) for the Py_2_-DO and Py-PO samples, whose chemical structure is shown in Figure 1 and molecular parametrization is illustrated in Figure 2, are summarized in Table 1. They span over one order of magnitude, which should result in large differences in the <*k*> values retrieved with the MFA.

### 3.3. Analysis of the Fluorescence Spectra

**Figure 3:**
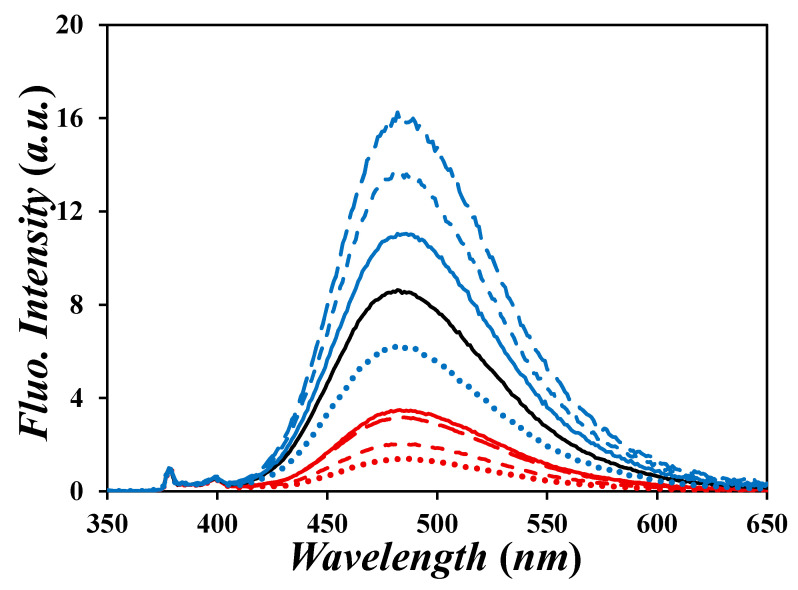
Steady-state fluorescence spectra of degassed solutions of the Py_2_-DO and Py-PO samples in THF with [*Py*] = 2.5 μM. *λ*_ex_ = 344 nm. From top to bottom: (long dashes, blue) Py_5_-Adonitol, (short dashes, blue) Py_4_-Erythritol, (solid line, blue) Py_6_-Sorbitol, (solid line, black) Py_2_-EG, (dots, blue) Py_3_-Glycerol, (solid line, red) Py_2_-ButDiol, (long dashes, red) Py_2_-HexDiol, (short dashes, red) Py_2_-DecDiol, and (dots, red) Py_2_-HexadecDiol.

The fluorescence spectra for each of the Py_2_-DO and Py-PO samples in THF are shown in Figure 3, with the fluorescence spectra of the Py_2_-DO and Py-PO samples in dioxane, DMF, and DMSO being shown in Figure S39 in the Supplementary Materials. All the fluorescence spectra were normalized to unity at either 378 or 379 nm in THF and dioxane or DMF and DMSO, respectively, which corresponds to the 0–0 transition of the pyrene monomer. Each spectrum displayed the characteristic features of a pyrene-labeled species, including the sharp monomer peaks from 375 to 400 nm and the large structureless excimer emission centered at 480 nm. The relative excimer intensities were highest for the Py_2_-DO and Py-PO samples in THF (*η* = 0.46 mPa.s at 25 °C) [33] and lowest for those in DMSO (*η* = 1.99 mPa.s at 25 °C) [33], indicative of the low and high viscosities, respectively, either aiding or hindering the diffusion-controlled PEF. The fluorescence spectra acquired for Py_2_-DO and Py-PO solutions in DMF (*η* = 0.79 mPa.s at 25 °C) [33] and dioxane (*η* = 1.20 mPa.s at 25 °C) [33], two solvents with viscosities that were intermediate between those of THF and DMSO, yielded intermediate excimer fluorescence intensities in comparison.

According to Equation (1), the fluorescence intensity of the pyrene excimer (*I*_E_) relative to that of the pyrene monomer (*I*_M_) is proportional to [*Py*]_loc_. Consequently, Py_6_-Sorbitol with six pyrenyl labels should generate more excimer than Py_5_-Adonitol and Py_4_-Erythritol with five and four pyrenyl labels, respectively. This is not observed in Figure 3. A similar sequence inversion is observed when comparing *I*_E_ for Py_2_-EG with that for Py_3_-Glycerol, with the former being larger than the latter, contrary to expectations based on [*Py*]_loc_. Such inconsistencies are common for the analysis of fluorescence spectra with pyrene-labeled molecules, where the presence of minute quantities of unreacted pyrene derivative results in a large increase in *I*_M_, which affects the value of *I*_E_ relative to *I*_M_ [34,35].

**Figure 4:**
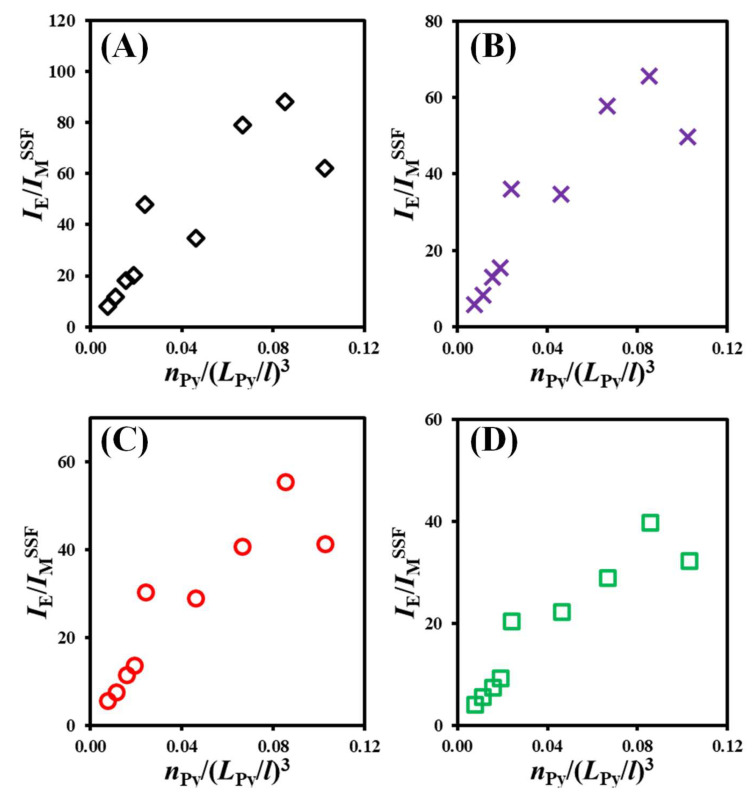
Plots of *I*_E_/*I*_M_^SSF^ as a function of *n*_Py_/(*L*_Py_/*l*)^3^ for degassed solutions of the Py_2_-DO and Py-PO samples with [*Py*] = 2.5 μM in (**A**) THF, **(B**) dioxane, (**C**) DMF, and (**D**) DMSO.

The *I*_E_/*I*_M_ ratios obtained through integration of the steady-state fluorescence (SSF) spectra (*I*_E_/*I*_M_^SSF^) are plotted as a function of *n*_Py_/(*L*_Py_/*l*)^3^ in Figure 4. While there was a general increase in *I*_E_/*I*_M_^SSF^ with increasing *n*_Py_/(*L*_Py_/*l*)^3^ as predicted by Equation (1), there was a notable degree of scatter resulting from the presence of small amounts of unattached pyrene derivative impurities in the samples. This artifact has arisen in numerous earlier publications and highlights the main drawback of using the fluorescence spectra of pyrene-labeled constructs to conduct more quantitative studies by PEF [17,29,30,31,32,34,35].

Despite their very low molar percentage relative to the pyrenyl labels covalently bound to the diols and polyols (usually ≤ 1% as determined through the global MFA of the monomer and excimer fluorescence decays), these impurities contribute an outsized amount to the monomer emission in the fluorescence spectra. This outcome is a consequence of their long natural monomer lifetime τ_M_ (~200 ns) and associated large fluorescence quantum yield (*q*_F_) relative to the *q*_F_ of the pyrene monomers bound to the polyols with a much shorter monomer lifetime τ_M_ (<5 ns). Since *q*_F_ is proportional to the lifetime [36], a 40-fold increase in τ_M_ meant that one free pyrene derivative in the solvent emits as strongly as 40 pyrenyl labels bound to a polyol. This increased monomer emission thus lowers the observed *I*_E_/*I*_M_^SSF^ ratio due to the uncontrolled minute quantities of unattached pyrenyl labels present in the Py_2_-DO and Py-PO solutions. These free pyrenyl labels give rise to the non-linear *I*_E_/*I*_M_^SSF^-vs.-*n*_Py_/(*L*_Py_/*l*)^3^ trends obtained for the larger [*Py*]_loc_ values corresponding to the polyols in Figure 4.

As was pointed out earlier, the effect that small quantities of unattached pyrene derivatives have on the *I*_E_/*I*_M_^SSF^ ratio is much more pronounced for pyrene-labeled molecules having short decay times for the pyrene monomer, such as those observed for the branched Py-PO constructs [35]. It is thus not surprising to observe more scatter in the *I*_E_/*I*_M_^SSF^-vs.-*n*_Py_/(*L*_Py_/*l*)^3^ plots in Figure 4 for the branched Py-PO samples than for the linear Py_2_-DO samples, which yield relatively longer decay times. Since steady-state fluorescence alone cannot differentiate between the monomer signal emanating from the Py_2_-DO and Py-PO samples or the free pyrene impurities, the *I*_E_/*I*_M_^SSF^-vs.-*n*_Py_/(*L*_Py_/*l*)^3^ trends shown in Figure 4 can only be interpreted qualitatively. Quantitative information about the kinetics of PEF for the Py_2_-DO and Py-PO samples can only be retrieved through the global MFA of the fluorescence decays of the pyrene monomer and excimer, as described in the next sections.

### 3.4. Model-Free Analysis of the Fluorescence Decays

**Figure 5:**
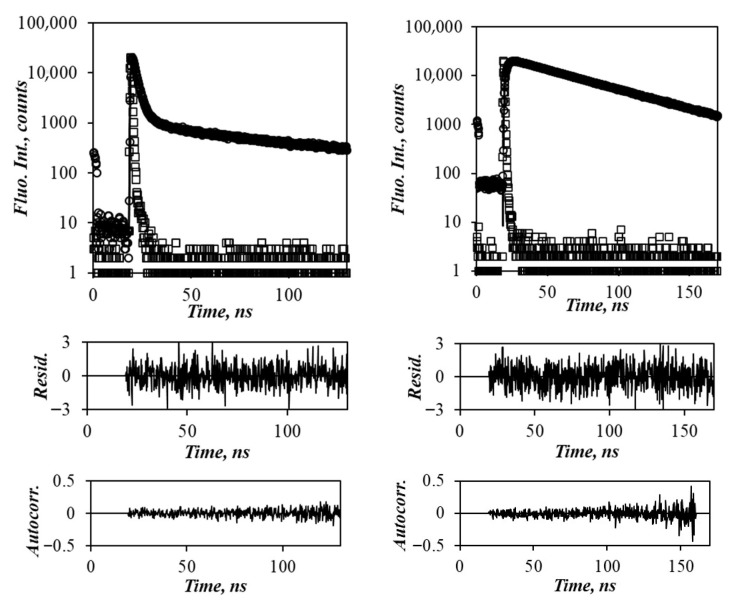
Monomer (**left**) and excimer (**right**) fluorescence decays for Py_3_-Glycerol in THF. [*Py*] = 2.5 μM, λ_ex_ = 336 nm, χ^2^ = 0.97.

The monomer and excimer fluorescence decays of the Py_2_-DO and Py-PO solutions in each solvent were fit globally with the MFA according to Equations (S1) and (S2) given in the Supplementary Materials. An example fit of the monomer and excimer fluorescence decays of the Py_3_-Glycerol sample according to the MFA is presented in Figure 5. Although there was a slight increase in the aggregation of the pyrenyl labels for the branched Py-PO constructs, which were more densely labeled and had a higher [*Py*]_loc_ than the linear Py_2_-DO samples, the *f*_agg_ values (see Figure S40 in the Supplementary Materials) remained small in comparison to *f*_diff_ for all samples in each solvent, indicating that PEF occurred mainly through diffusive encounters between the pyrenyl labels.

**Figure 6:**
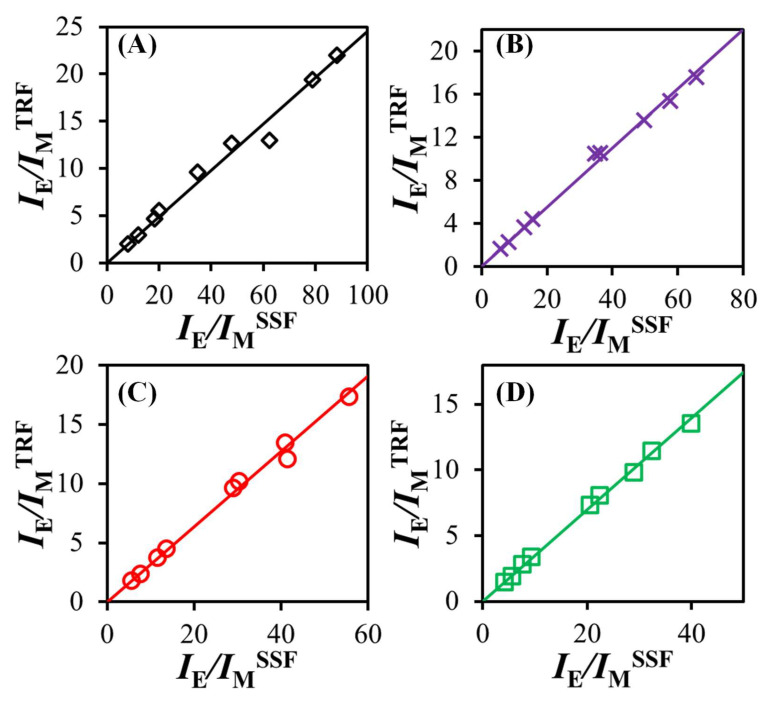
Plots of *I*_E_/*I*_M_^TRF^ vs*. I*_E_/*I*_M_^SSF^ for degassed solutions of the Py_2_-DO and Py-PO samples with [*Py*] = 2.5 μM in (**A**) THF, (**B**) dioxane, (**C**) DMF, and (**D**) DMSO.

While the MFA of the fluorescence decays for all the Py_2_-DO and Py-PO samples in each solvent yielded extremely low *f*_free_ values (≤0.01) highlighting the efficient removal of the pyrene derivatives used in the labeling reactions from the Py_2_-DO and Py-PO products during the purification, the small *f*_free_ values were enough to generate the scatter seen in the *I*_E_/*I*_M_^SSF^-vs.-*n*_Py_/(*L*_Py_/*l*)^3^ trends in Figure 4 at high [*Py*]_loc_. The molar fraction *f*_free_ and all the other parameters retrieved from the global MFA of the time-resolved fluorescence (TRF) decays could be combined into Equation (6) to obtain the absolute *I*_E_/*I*_M_^TRF^ ratio for the Py_2_-DO and Py-PO samples in each solvent. The *I*_E_/*I*_M_^TRF^ ratios are plotted against *I*_E_/*I*_M_^SSF^ for comparison in Figure 6.

(6) $${\left(\frac{I_{E}}{I_{M}}\right)}^{TRF}\;=\;\frac{(f_{diffE0}\tau_{E0}+f_{diffD}\tau_{D})<k><\tau >+f_{E0}\tau_{E0}+f_{D}\tau_{D}}{(f_{diffE0}+f_{diffD})<\tau >+f_{free}\tau_{M}}$$

The *I*_E_/*I*_M_^TRF^-vs.-*I*_E_/*I*_M_^SSF^ trends in Figure 6 yielded Pearson correlation coefficients equal to 0.991, 0.996, 0.998, and 0.999 in THF, DMF, dioxane, and DMSO, respectively. The Pearson correlation coefficients being so close to unity confirmed that the photophysical processes resulting in the fluorescence spectra shown in Figure 3 were well represented by the MFA parameters and that these parameters could be considered for further analysis.

**Figure 7:**
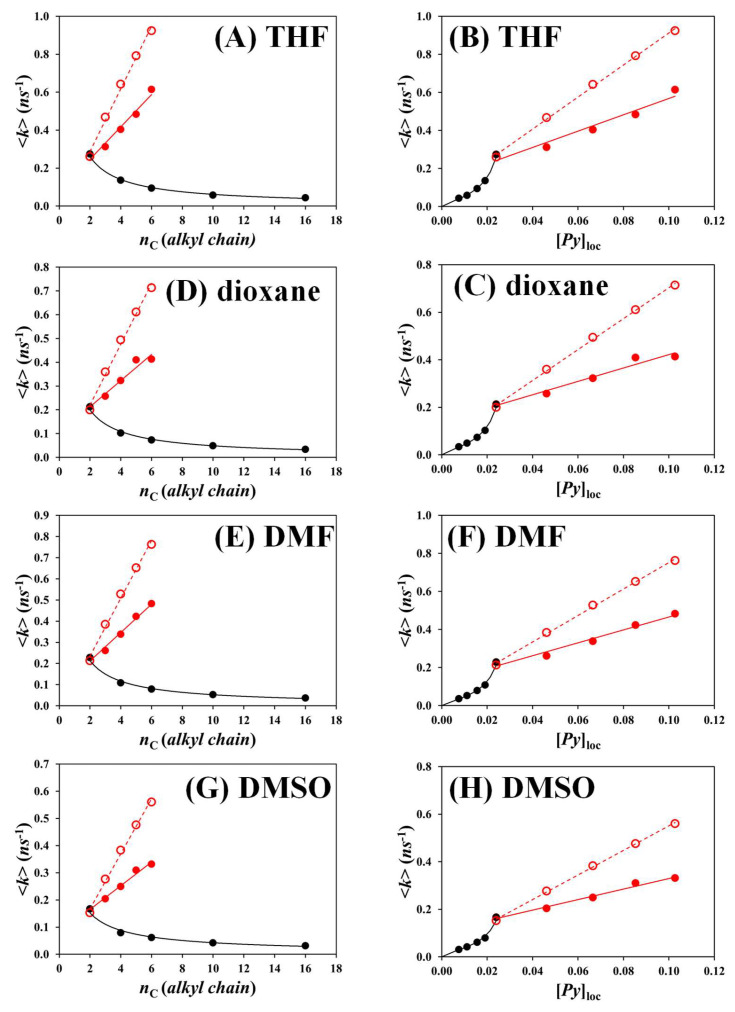
Plots of experimental (filled) and theoretical (hollow) <*k*> in (**A**,**B**) THF, (**C**,**D**) dioxane, (**E**,**F**) DMF, and G,H) DMSO as a function of (**A**,**C**,**E**,**G**) *n*_C_ and (**B**,**D**,**F**,**H**) [*Py*]_loc_ for the (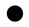) Py_2_-DO and (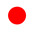,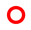)Py-PO samples. The black solid line represents the function *A *× *n*_C_^−*B*^ used to fit the experimental <*k*> values obtained for the Py_2_-DO samples.

Plots of <*k*> as a function of the number (*n*_C_) of carbon atoms of the main alkyl chain in the diols and polyols and [*Py*]_loc_ are presented in Figure 7 for the Py_2_-DO and Py-PO samples in the four solvents considered and show similar behavior in each solvent. The <*k*>-vs.-*n*_C_ trends are discussed first. The <*k*>-vs.-*n*_C_ profiles were very different for the linear Py_2_-DO and branched Py-PO samples, with <*k*> increasing and decreasing with increasing *n*_C_, respectively.

For the same *n*_C_, <*k*> was much larger for a branched Py-PO than for a linear Py_2_-DO construct. This result illustrates the significant increase in [*Py*]_loc_ for the branched Py-PO constructs, where each carbon in the polyol backbone bears a pyrenyl label, contrary to the Py_2_-DO samples that only contain two pyrenes held far apart from each other. Whereas adding one carbon atom to the alkyl chain of the diols reduces [*Py*]_loc_, it increases [*Py*]_loc_ for the polyols.

### 3.5. Molecular Parametrization of the Constructs to Calculate <k>^th^

Interestingly, the <*k*> values for the Py_2_-DO samples were well represented by the function *A* × *n*_C_^−B^ for 2 ≤ *n*_C_ ≤ 16, whose parameters *A* and *B* have been listed in Table 2. In turn, this function provided a mathematical means to estimate the <*k*>(*i*) value of a Py_2_-DO sample made from an oligomethylene chain containing *n*_c_ = *i* carbons.

**Figure 8:**
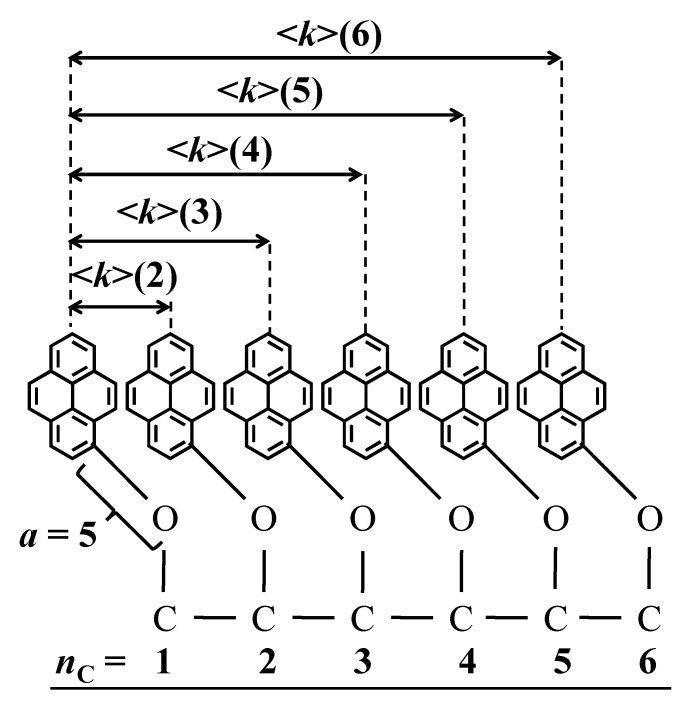
Molecular parametrization of the branched Py-PO constructs.

The <*k*>(*i*) values were used to predict the <*k*> value for a branched Py-PO construct based on the molecular parametrization presented in Figure 8. Taking the pyrenyl label attached to carbon (3) as an example, it could form an excimer with the pyrenyl labels attached to carbons (2) and (4) with the rate constant 2 × <*k*>(2), with the pyrenyl labels attached to carbons (1) and (5) with the rate constant 2 × <*k*>(3), and with the pyrenyl label attached to carbon (6) with the rate constant 1 × <*k*>(4). After all the pyrenyl labels attached to a polyol were considered and all their contributions were averaged, Equation (7) was derived.

Equation (7) was applied to predict the <*k*>^th^ values of the branched Py-PO samples shown as the hollow red circles in Figure 7. Both the theoretical and experimental <*k*> values obtained for the Py-PO constructs with Equation (6) increased linearly with *n*_C_ and [*Py*]_loc_. The slope obtained for the theoretical <*k*>-vs.-[*Py*]_loc_ straight lines after averaging for all solvents was always larger than the slope for the experimental <*k*>-vs.-[*Py*]_loc_ straight lines, suggesting that PEF was less efficient than theoretically expected for the Py-PO samples.

The same <*k*> values were compared to [*Py*]_loc_ calculated with Equations (2)–(5). <*k*> was found to increase linearly with [*Py*]_loc_ for larger *n*_C_ and thus lower [*Py*]_loc_ for the Py_2_-DOs, but for *n*_C_ equal to 2 and 4, a clear deviation from linearity was observed. The linear relationship between <*k*> and [*Py*]_loc_ is reasonable since <*k*> has been reported to scale with the number (*N*) of segments in a linear chain as *N*^α^, where *α* has been reported to range from 0.9 to 1.9 [37,38,39,40,41,42,43,44], as would be predicted by Equations (2) and (3). The uptick observed in Figure 7B,D,F,H for *n*_C_ equal to 2 and 4 indicates a deviation from this relationship, which is attributed to an increase in *k*_diff_ in Equation (1). As mentioned earlier, the shorter linker between the two pyrenyl labels must reduce the number of conformations available to the linker, which results in a faster path toward PEF associated with a larger *k*_diff_ value. The fact that the pyrenyl labels separated by 14 or fewer atoms have a larger *k*_diff_ value affects all the <*k*> values of the Py-PO samples since most of their pyrenyl labels are separated by 14 or fewer atoms. This results in a steep and linear increase in the <*k*> values of the Py-PO constructs. However, while the linear increase in <*k*> with respect to [*Py*]_loc_ is predicted by Equation (1), the resulting straight line no longer passes through the origin. It is also responsible for the clear breakpoint marking the transition from the linear diols with *n*_C_ equal to 6, 8, and 16 corresponding to the low-[*Py*]_loc_ regime of the Py_2_-DOs and the branched Py-PO polyols at high [Py]_loc_.

**Figure 9:**
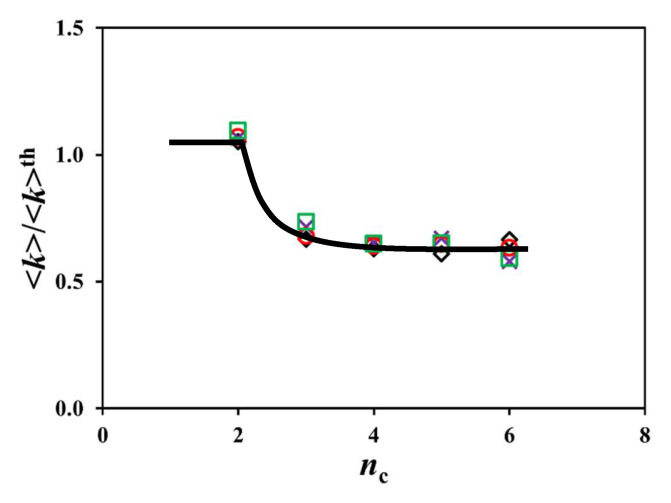
Plot of <*k*>/<*k*>^th^ as a function of *n*_c_ for the Py-PO constructs in (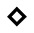) THF, (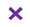) dioxane, (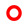) DMF, and (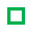) DMSO.

The experimental <*k*> values obtained for the Py_2_-DO samples were used to predict the <*k*>^th^ values expected for the Py-PO constructs by applying Equation (7). The <*k*>^th^ values predicted with Equation (6) agreed qualitatively with the experimental <*k*> values, increasing linearly with increasing [*Py*]_loc_ in Figure 7B,D,F,H. However, the slope of the straight lines obtained for the experimental <*k*> values was 53 (±4) % lower than the slope of the straight lines for <*k*>^th^ after averaging for the trends in all solvents, suggesting that PEF was not as efficient as it should have been. This effect is illustrated in Figure 9, where the <*k*>/<*k*>^th^ ratio is plotted as a function of *n*_C_ for all of the Py-PO samples in each solvent. All the <*k*>/<*k*>^th^ ratios clustered around a master curve, showing that the same reduction in <*k*> was observed in all solvents and was not a consequence of solvent interactions with the Py-PO samples. The reduction in <*k*> must be a consequence of steric hindrance, whereby pyrenyl labels neighboring an excited pyrene hinder the access of pyrenyl labels that are further away along the polyol backbone, an effect which is not accounted for in the calculation of <*k*>^th^ with Equation (7).

(7) $$<k{>}^{th}\;=\;\frac{2}{n_{C}}\sum_{i=1}^{n_{C}-1}\left(n_{C}-i)\times <k>(i+1\right)$$

**Figure 10:**
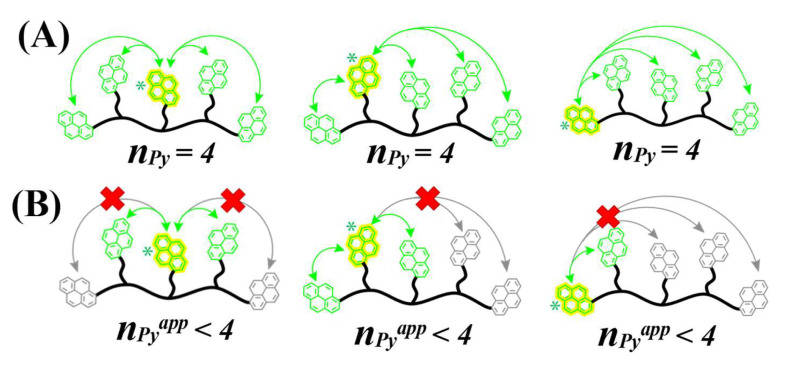
Depiction of (**A**) unhindered and (**B**) hindered PEF using Py_5_-Adonitol as example. Pyrenes in yellow, green, and gray are excited (*), unhindered, and sterically hindered, respectively. Red cross indicates sterically hindered access of ground-state pyrenes to excited pyrene.

The effect that steric hindrance might have on PEF by diffusive encounters between an excited pyrene and ground-state pyrenyl labels further away along the polyol backbone is illustrated in Figure 10 for Py_5_-Adonitol (see chemical structure in Figure 1). Exciting one of the five pyrenyl labels of Py_5_-Adonitol would yield an *n*_Py_ value of four ground-state pyrenes with three possible configurations reflecting the three possible positions of the excited pyrenyl label. If all *n*_Py_ = 4 ground-state pyrenyl labels had equivalent access to the excited pyrene, then <*k*> should exhibit a strong response to *n*_Py_ since [*Py*]_loc_ is directly proportional to *n*_Py_ in Equation (2). This is observed for the predicted <*k*>^th^-vs.-[*Py*]_loc_ trends in Figure 7B,D,F,H, which show a steep increase in <*k*>^th^ with increasing [*Py*]_loc_. The fact that a 53% smaller slope was obtained for the experimental <*k*>-vs.-[*Py*]_loc_ trends compared to the <*k*>^th^-vs.-[*Py*]_loc_ trends supports the notion that not all pyrenyl labels are equivalent along the polyol backbone and that neighboring ground-state pyrenes must react preferentially with an excited pyrenyl label as they hinder PEF with ground-state pyrenyl labels located further away from the excited pyrene. A depiction of the proposed effect is presented in Figure 10B.

### 3.6. Accounting for Solvent Effects

While the <*k*>-vs.-[*Py*]_loc_ trends shown in Figure 7B,D,F,H provide an informative means to characterize the internal dynamics and conformation of the Py_2_-DO and Py-PO constructs, they do not address the possibility that solvent effects might contribute to these trends. In particular, *k*_diff_ in Equation (1) is proportional to *p*/*η*, where *p* is the probability of excimer formation upon an encounter between an excited and a ground-state pyrene [36] and *η* is the solvent viscosity. It follows that since PEF is diffusion-controlled, the rate constant *k*_diff_ in Equation (1) for diffusive PEF and thus the <*k*> values and *I*_E_/*I*_M_ ratios are all inversely proportional to the solvent viscosity. The *p* parameter, however, is more related to the solvent polarity stabilizing the formation of the excimer complex, with more polar solvents usually having larger *p* values resulting in higher *k*_diff_ values. These differences have been accounted for in the past through the determination of *k*_diff_ in a solvent of interest using a small pyrene derivative, whose solution concentration ([*Py*]) is known and equals [*Py*]_loc_. Since [*Py*] is known experimentally, a plot of <*k*>-vs.-[*Py*] for the pyrene derivative yields a straight line with a slope equal to *k*_diff_. Pyrene derivatives such as *n*-hexyl-1-pyrenebutyramide, ethyl-1-pyreneoctanamide, ethyl-1-pyrenedodecanamide, and ethyl-1-pyrenebutyrate (PyBE) have been prepared previously [31], and their *k*_diff_ values were determined in different solvents. In the case of PyBE, which is more relevant to the Py_2_-DO and Py-PO samples, *k*_diff_ values of 1.90, 1.42, and 1.00 M^−1^.ns^−1^ were obtained in THF, DMF, and DMSO, respectively. To determine *k*_diff_ for PyBE in dioxane, the compound was synthesized again, and its monomer and excimer fluorescence decays were acquired in the front-face geometry to avoid the inner filter effect at the much larger PyBE concentrations used in these experiments ranging from 6.5 to 12.5 mM. After <*k*> was determined from the global MFA of the fluorescence decays, a plot of <*k*>-vs.-[*Py*] (see Figure S41 in the Supplementary Materials) yielded a slope of 1.40 M^−1^.ns^−1^, which was taken as the *k*_diff_ value for PyBE in dioxane.

**Figure 11:**
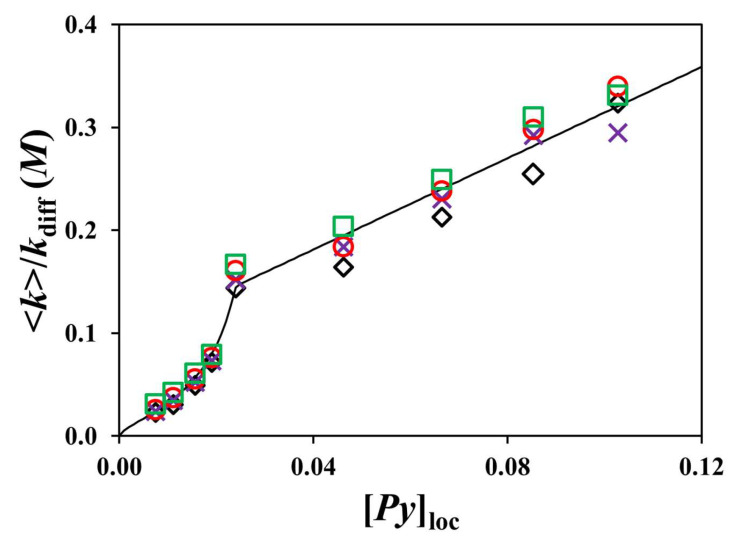
Plot of <*k*>/*k*_diff_ as a function of [*Py*]_loc_ for degassed solutions of the Py_2_-DO and Py-PO samples with [*Py*] = 2.5 μM in (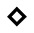) THF, (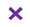) dioxane,(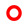) DMF, and (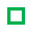) DMSO.

Since the *k*_diff_ values obtained in all solvents account for the *p*/*η* ratios, which are solvent-dependent and should affect PEF in a similar manner, the <*k*> values were divided by the respective *k*_diff_ value in a given solvent, and the <*k*>/*k*_diff_ ratio is plotted as a function of [*Py*]_loc_ in Figure 11. All <*k*>/*k*_diff_ values collapsed on a master curve reflecting the general trends highlighted in Figure 7B,D,F,G, namely that <*k*>/*k*_diff_ was well represented by a function of the type *A* × *n*_C_^−B^ for the Py_2_-DO samples and a straight line for the Py-PO constructs. This result implies that the solvents used in the study are not perturbing the rate of PEF in the Py-PO samples beyond the degree expected from their differences in polarity and viscosity and that the conclusions drawn from the <*k*> ratios reflect the properties of the Py_2_-DO and Py-PO samples.

While the plots shown in Figure 6D,F,H, Figure 7B and Figure 11 clearly illustrate a breakdown of the <*k*>-vs.-[*Py*]_loc_ relationship since no linear trend was observed between <*k*> and [*Py*]_loc_ over the range of [*Py*]_loc_ investigated, the existence of the <*k*>-vs.-[*Py*]_loc_ relationship was instrumental in identifying these constructs that deviated from a linear trend. In particular, analysis of the deviations from the expected <*k*>-vs.-[*Py*]_loc_ linear relationship led to a physical understanding of their origin, such as a reduction of the number of pathways leading to an increase in the PEF efficiency for the Py_2_-ButDiol and Py_2_-EG constructs with a shorter butylene or ethylene chain and steric hindrance for the Py-PO samples resulting in a decrease in the PEF efficiency. The increase in <*k*> found for the shorter Py_2_-ButDiol and Py_2_-EG samples shifted the <*k*> values of all the Py-PO constructs up, resulting in a straight <*k*>-vs.-[*Py*]_loc_ line that no longer passed through the origin. Such a detailed understanding of the subtle effects leading to PEF in these pyrene-labeled multifunctional oligomers indicates that the <*k*>-vs.-[*Py*]_loc_ relationship represents an excellent investigative tool to better understand the behavior of not only the large macromolecules studied earlier [17,29,30,31,32], but also multifunctional oligomers like the diols and polyols investigated herein.

## 4. Conclusions

The inherent relationship existing between <*k*> and [*Py*]_loc_ established experimentally for larger pyrene-labeled macromolecules [17,29,30,31,32] appears to be an appealing tool for probing the conformation and internal dynamics of multifunctional oligomers. The different architectures of the pyrene-labeled linear diols and branched polyols had a dramatic effect on PEF. Since each carbon atom of the polyol backbone bore a 1-pyrenebutanoate derivative, an increase in the number (*n*_C_) of carbons in the polyol chain led to a massive and linear increase in <*k*> in Figure 7A,C,E,G, since [*Py*]_loc_ is directly proportional to the number (*n*_Py_) of ground-state pyrenyl labels according to Equation (2), which equaled *n*_C_–1. In contrast, increasing *n*_C_ for the diols had the opposite effect of decreasing <*k*> with increasing *n*_C_ since increasing the length of the diol backbone kept the two pyrenyl labels apart.

The different behaviors between the diols and polyols were also observed in the <*k*>-vs.-[*Py*]_loc_ plots. Whereas <*k*> scaled as *L*_Py_^−3/2^, indicating that <*k*> was proportional to [*Py*]_loc_ for the longer diols, as has been reported for other small linear alkyl chains [44], the <*k*>-vs.-[*Py*]_loc_ plots showed an upward increase for the shorter diols, which reflected faster PEF, probably due to the reduction in the number of conformations available to the linear linker between the two pyrenyl end groups. The steep increase in <*k*> for the Py_2_-EG sample pushed up the <*k*> values of the Py-PO samples, which increased linearly with increasing [*Py*]_loc_ but did not pass through the origin. The slope of the <*k*>-vs.-[*Py*]_loc_ straight lines was not as large as expected, however, indicating that PEF was less effective with increasing *n*_C_. This effect, depicted in Figure 10, was attributed to the steric hindrance generated by the ground-state pyrenyl labels neighboring the excited pyrene as they restrict access to the excited pyrene by other ground-state pyrenyl labels located further away along the polyol backbone. Consequently, the kinetics for PEF appear to be quite complex for the Py-PO constructs, with the small *L*_Py_ distance separating every two pyrenyl labels resulting in a larger <*k*>, but with steric hindrance generated by those pyrenyl labels neighboring an excited pyrene leading to a reduction in <*k*>. The compounding of both effects yields the complex trends shown for the <*k*>-vs.-[*Py*]_loc_ plots in Figure 7 and Figure 11. Such effects would be challenging to identify by techniques other than PEF, which demonstrates its value in the study of these complex multifunctional oligomers.

Since PEF is a well-known form of fluorescence collisional quenching (FCQ), the experiments described herein with PEF could in principle be conducted with other FCQ processes. The main difference is that most FCQ phenomena do not yield a fluorescent product like the pyrene excimer. Consequently, <*k*> would be obtained through single decay analysis of the dye being quenched by the selected FCQ process and could not be as accurate as the <*k*> values retrieved from the global MFA of the pyrene monomer and excimer fluorescence decays. That said, good correlations have been obtained between the <*k*> values retrieved from the multiexponential analysis of single fluorescence decays of the pyrene monomer and those obtained through the MFA, suggesting that single decay analysis of FCQ events should provide a reasonable representation for <*k*> [29]. Of course, one also needs to acknowledge that the probability (*p*) of quenching upon encounter will likely be different for other dyes and quenchers compared to the *p* value for PEF. But these differences can easily be accounted for by using model compounds such as the PyBE sample used to normalize the <*k*> trends in Figure 11.

## Figures and Tables

**Table 1:** *n*_Py_/(*L*_Py_/*l*)^3^ values calculated for the Py_2_-DO and Py-PO samples using Equations (2)–(5).

Py-PO	*n*_C_	*n*_Py_	*n*_Py_/(*L*_Py_/*l*)^3^
**Py_2_-HexadecDiol**	16	1	0.008
**Py_2_-DecDiol**	10	1	0.011
**Py_2_-HexDiol**	6	1	0.016
**Py_2_-ButDiol**	4	1	0.019
**Py_2_-EG**	2	1	0.024
**Py_3_-Glycerol**	3	2	0.046
**Py_4_-Erythritol**	4	3	0.067
**Py_5_-Adonitol**	5	4	0.085
**Py_6_-Sorbitol**	6	5	0.103

**Table 2:** Parameters used to fit the <*k*> = *A* × *n*_C_^−*B*^ trends in Figure 7A,C,E,G.

Solvent	A (ns^−1^)	B
THF	0.484	0.895
dioxane	0.367	0.876
DMF	0.391	0.873
DMSO	0.264	0.792

## Data Availability

All fluorescence spectra and decays used in this study are provided as Supplementary Materials with Excel files for Py-PO SSF and Py-PO TRF, respectively.

## Supplementary Materials

The following supporting information can be downloaded at https://www.mdpi.com/article/10.3390/polym17141979/s1: Description of the synthesis for the Py_2_-DO and Py-PO samples; Figures S1–S22: ^1^H NMR and COSY spectra of Py-BE, Py_2_-HexadecDiol, Py_2_-DecDiol, Py_2_-HexDiol, Py_2_-ButDiol, Py_2_-EG, Py_3_-Glycerol, Py_4_-Erythritol, Py_5_-Adonitol, Py_6_-Sorbitol; Figures S23–S38: MS^n^ spectra of Py-BE, Py_2_-HexadecDiol, Py_2_-DecDiol, Py_2_-HexDiol, Py_2_-ButDiol, Py_2_-EG, Py_3_-Glycerol, Py_4_-Erythritol, Py_5_-Adonitol, Py_6_-Sorbitol; Figure S39: Fluorescence spectra of the Py_2_-DO and Py-PO samples; Equations used in the MFA; Tables S1–S3: Parameters retrieved from the MFA of the fluorescence decays; Figure S40: Molar fraction of the pyrenyl species in the Py_2_-DO and Py-PO solutions; Figure S41: Plot of <*k*>-vs.-[*Py-BE*].

## Abbreviations

The following abbreviations are used in this manuscript:

*I*_E_/*I*_M_	Excimer-to-monomer intensity ratio
*k*_diff_	Rate constant for diffusive PEF
<*k*>	Average rate constant for PEF
MFA	Model-free analysis
PEF	Pyrene excimer formation
Py_2_-DO	Pyrene end-labeled diol
PyLM	Pyrene labeled macromolecule
[*Py*]_loc_	Local concentration of ground-state pyrenes
Py-PO	Pyrene end-labeled polyol
*R*_G_	Radius of gyration
SSF	Steady-state fluorescence
TRF	Time-resolved fluorescence
[*η*]	Intrinsic viscosity
