# Supplementary material for: Internal Dynamics of Pyrene-Labeled Polyols Studied Through the Lens of Pyrene Excimer Formation
Source: Polymers (Basel). 2025 Jul 18;17(14):1979. doi: 10.3390/polym17141979 (PMC12298612; doi:10.3390/polym17141979)
Supplement: Supplementary file 1 [file polymers-17-01979-s001.zip › polymers-3720663-supplementary.pdf]

Supporting Materials for

Dynamics of Pyrene-labeled Polyols

Studied Through the Lens of Pyrene Excimer Fluorescence

Franklin Frasca and Jean Duhamel

Institute for Polymer Research, Waterloo Institute for Nanotechnology, Department of Chemistry, University of Waterloo, Waterloo, ON, N2L 3G1, Canada

Table of Contents

A) Synthesis of the Py_2-DO and Py-PO samples 2

B) ^{1}H NMR and COSY Spectra of the Py_2-DO and Py-PO samples 7

C) MS^n Fragmentation of the Py_2-DO and Py-PO samples and Discussion 29

D) Fluorescence spectra of the Py_2-DO and Py-PO sampless in dioxane, DMF, and DMSO 46

E) Equations used for the global MFA of the monomer and excimer fluorescence decays 47

F) Equations used to calculate the molar fractions f_{diffE0}, f_{diffD}, f_{free}, f_{E0}, f_D, f_{diff}, and f_{agg} 48

G) Parameters obtained from the Model Free Analysis of the Py_2-DO and Py-PO samples 49

H) Molar Fractions of the Py_2-DO and Py-PO samples obtained from the MFA 52

I) Determination of k_{diff} for PyBE in dioxane 53

A) Synthesis of the Py_2-DO and Py-PO samples

Synthesis of ethyl 4-(pyren-1-yl)butanoate (*Py-BE*). 1-Pyrene butyric acid (PyBA, 1.01 g, 3.52 mmol, 1.0 eq), ethanol (EtOH, 1.70 g, 36.9 mmol, 10.5 eq), and 4-dimethylaminopyridine (DMAP, 0.63 g, 5.2 mmol, 1.5 eq) were added to 10 mL of distilled dichloromethane (DCM) in a 20 mL scintillation vial to form a brown solution upon stirring. *N*-Ethyl-*N*'-(3-dimethylaminopropyl)carbodiimide hydrochloride (EDC·HCl, 1.08 g, 5.66 mmol, 1.6 eq) was then added to the reaction vessel and the vessel wall was rinsed with 4 mL of distilled DCM. The reaction vessel was stirred overnight under a cover of aluminum foil to minimize photobleaching of the pyrenyl groups. The reaction mixture was transferred to a separatory funnel and diluted with 80 mL of DCM for workup. The organic layer was washed with a 2 M hydrochloric acid (HCl) solution (1 x 30 mL) and brine (1 x 30 mL) before drying over Na_2SO_4 and condensing on a rotary evaporator. The product was purified via silica gel chromatography using a 9:1 hexanes:ethyl acetate mixture as eluent. Condensing the fractions yielded white-yellow crystals which were isolated via suction filtration (Whatman #5), while evaporation of the filtrate yielded more white-yellow crystals. Both fractions were dried to completion in a vacuum oven at room temperature overnight to yield white-yellow crystals having identical ^{1}H NMR and COSY spectra (see Figures S1 and S2). (745.8 mg, 67%). ^{1}H NMR (300MHz, $CDCl_3$): δ = 8.43 – 7.83 (m, 9H), 4.26 – 4.11 (q, J = 7.1 Hz, 2H), 3.49 – 3.36 (t, J = 7.7 Hz, 2H), 2.57 – 2.38 (t, J = 7.3 Hz, 2H), 2.32 – 2.12 (tt, J = 7.7, 7.3 Hz, 2H), 1.37 – 1.22 (t, J = 7.1 Hz, 3H) ppm. **HRMS** (ESI+): calc'd for $C_{22}H_{20}O_2$ [M^+] 316.1458, found 316.1466, calc'd for $C_{22}H_{20}O_2Na$ [$M+Na^+$] 339.1356, found 339.1354, calc'd for $C_{22}H_{20}O_2K$ [$M+K^+$] 355.1095, found 355.1091.

General Procedure for the synthesis of the Py_2-DO and Py-PO samples: To a 20 mL scintillation vial equipped with a stir bar, PyBA (4.0 eq), hydroxybenzotriazole monohydrate (0.6 eq), and the diol or polyol expressed in terms of their hydroxyl content (a 2:1 PyBA:ROH molar ratio) were added to 10 mL of distilled DCM. The mixture was stirred to form a brown/orange dispersion. Then diisopropylethylamine (DIPEA, 6.0 eq) was added and the brown/orange precipitate dissolved to form a brown/orange solution. After 5 – 10 minutes EDC·HCl (6.0 eq) was added to the reaction mixture before allowing it to stir overnight. The overall PyBA:ROH:HOBt·H_2O:DIPEA:EDC·HCl molar composition for the reaction mixture would thus be a 2:1:0.3:3:3 ratio. The next day, 2 mL of toluene was added to the reaction mixture before

evaporating the DCM under a flow of nitrogen. The residual crude product mixture was purified via column chromatography on silica gel (230 – 400 mesh size) using either toluene or a specified toluene:acetone mixture as eluent. The fractions with product were concentrated either under a flow of nitrogen or in a rotary evaporator to ~ 5 mL for precipitation from 80 mL of cold hexanes. The precipitate was isolated via vacuum filtration (Whatman #1 or #5) to yield the product before drying overnight in a vacuum oven at room temperature.

Synthesis of Py_2-Hexadecane diol (*Py_2-HexadecDiol*): Py_2-HexadecDiol was prepared according to the general procedure from PyBA (0.30 g, 1.06 mmol), 1,16-hexadecane diol (65.6 mg, 0.25 mmol), HOBt·H_2O (23 mg, 0.15 mmol), DIPEA (0.3 mL, 1.72 mmol), and EDC·HCl (0.31 g, 1.64 mmol). The product was obtained as a white cakey solid (86.5 mg, 43 %). **^{1}H NMR** (300 MHz, $CDCl_3$) δ = 8.38 – 7.83 (m, 18H), 4.18 – 4.03 (t, *J* = 6.8 Hz, 4H), 3.54 – 3.34 (t, *J* = 7.7 Hz, 4H), 2.57 – 2.38 (t, *J* = 7.3 Hz, 4H), 2.30 – 2.13 (quintet, *J* = 7.5 Hz, 4H), 1.72 – 1.54 (quintet, *J* = 7.1 Hz, 4H), 1.49 – 1.10 (m, 24H) ppm. **HRMS** (ESI+): calc'd for $C_{56}H_{62}O_4$ [M^+] 798.4643, found 798.4656, calc'd for $C_{56}H_{62}O_4Na$ [$M+Na^+$] 821.4540, found 821.4551, calc'd for $C_{56}H_{62}O_4K$ [$M+K^+$] 837.4280, found: 837.4287.

Synthesis of Py_2-Decane diol (*Py_2-DecDiol*): The general procedure was followed by using PyBA (0.30 g, 1.06 mmol), 1,10-decane diol (44.2 mg, 0.25 mmol), HOBt·H_2O (23.4 mg, 0.15 mmol), DIPEA (0.3 mL, 1.72 mmol), and EDC·HCl (0.30 g, 1.58 mmol). The product was obtained as a white powder (65.5 mg, 36 %). **^{1}H NMR** (300 MHz, $CDCl_3$) δ = 8.42 – 7.80 (m, 18H), 4.14 – 4.01 (t, *J* = 6.7 Hz, 4H), 3.48 – 3.34 (t, *J* = 7.6 Hz, 4H), 2.55 – 2.38 (t, *J* = 7.1 Hz, 4H), 2.31 – 2.12 (quintet, *J* = 7.6 Hz, 4H), 1.70 – 1.50 (quintet, *J* = 6.8 Hz, 4H), 1.44 – 1.17 (m, 12H) ppm. **HRMS** (ESI+): calc'd for $C_{50}H_{50}O_4$ [M^+] 714.3704, found 714.3710, calc'd for $C_{50}H_{50}O_4Na$ [$M+Na^+$] 737.3601, found 737.3597, calc'd for $C_{50}H_{50}O_4K$ [$M+K^+$] 753.3341, found 753.3334.

Synthesis of Py_2-Hexane diol (*Py_2-HexDiol*): The synthesis was performed according to the general procedure from PyBA (0.20 g, 0.69 mmol), 1,6-hexane diol (20.9 mg, 0.18 mmol), HOBt·H_2O (17.7 mg, 0.12 mmol), DIPEA (0.2 mL, 1.15 mmol), and EDC·HCl (0.20 g, 1.06 mmol). The product was obtained as a white cakey solid (9.9 mg, 8 %). **^{1}H NMR** (300 MHz, $CDCl_3$) δ = 8.43 – 7.77 (m, 18H), 4.18 – 3.96 (t, *J* = 6.7 Hz, 4H), 3.52 – 3.26 (t, *J* = 7.6 Hz, 4H), 2.54 – 2.38 (t, *J* = 7.3 Hz, 4H), 2.30 – 2.10 (quintet, *J* = 7.6 Hz, 4H), 1.72 – 1.51 (quintet, *J* = 6.7 Hz, 4H), 1.46 – 1.28 (m, 4H) ppm. **HRMS** (ESI+): calc'd for $C_{46}H_{42}O_4$ [M^+] 658.3078, found 658.3094, calc'd for

$C_{46}H_{42}O_4Na$ [M+Na$^+$] 681.2975, found 681.2995, calc'd for $C_{46}H_{42}O_4K$ [M+K$^+$] 697.2715, found 697.2729.

Synthesis of Py$_2$-Butane diol (*Py$_2$-ButDiol*): Py$_2$-ButDiol was prepared according to the general procedure with PyBA (0.20 g, 0.69 mmol), 1,4-butane diol (16.1 mg, 0.18 mmol), HOBt·H_2O (16.2 mg, 0.11 mmol), DIPEA (0.2 mL, 1.15 mmol), and EDC·HCl (0.20 g, 1.16 mmol). The product was obtained as a white powder (12.0 mg, 11 %). **^{1}H NMR** (300 MHz, $CDCl_3$) δ = 8.32 – 7.78 (m, 18H), 4.18 – 4.02 (br. t, 4H), 3.44 – 3.28 (t, *J* = 7.7 Hz, 4H), 2.52 – 2.36 (t, *J* = 7.3 Hz, 4H), 2.28 – 2.08 (quintet, *J* = 7.5 Hz, 4H), 1.76 – 1.60 (m, 4H) ppm. **HRMS** (ESI+): calc'd for $C_{44}H_{38}O_4$ [M$^+$] 630.2765, found 630.2754, calc'd for $C_{44}H_{38}O_4Na$ [M+Na$^+$] 653.2662, found 653.2654.

Synthesis of Py$_2$-Ethylene Glycol (*Py$_2$-EG*): Py$_2$-EG was synthesized according to the general procedure with PyBA (0.20 g, 0.69 mmol), ethylene glycol (10.9 mg, 0.18 mmol), HOBt·H_2O (19.1 mg, 0.12 mmol), DIPEA (0.2 mL, 1.15 mmol), and EDC·HCl (0.20 g, 1.16 mmol). The product was obtained as a cream-colored powder (38.8 mg, 37 %). **^{1}H NMR** (300 MHz, $CDCl_3$) δ = 8.30 – 7.73 (m, 18H), 4.47 – 4.23 (s, 4H), 3.45 – 3.23 (t, *J* = 7.7 Hz, 4H), 2.58 – 2.37 (t, *J* = 7.3 Hz, 4H), 2.30 – 2.05 (quintet, *J* = 7.5 Hz, 4H) ppm. **HRMS** (ESI+): calc'd for $C_{42}H_{34}O_4$ [M$^+$] 602.2452, found 602.2439, calc'd for $C_{42}H_{34}O_4Na$ [M+Na$^+$] 625.2349, found 625.2349, calc'd for $C_{42}H_{34}O_4K$ [M+K$^+$] 641.2089, found 641.2081.

Synthesis of Py$_3$-Glycerol. The general procedure was applied to synthesize Py$_3$-Glycerol with PyBA (0.20 g, 0.69 mmol), glycerol (9.1 mg, 0.10 mmol), HOBt·H_2O (21.2 mg, 0.14 mmol), DIPEA (0.2 mL, 1.15 mmol), and EDC·HCl (0.20 g, 1.16 mmol). Column chromatography was performed using a 98:2 toluene:acetone mixture as eluent. The product was isolated as a white powder (5.5 mg, 6 %). **^{1}H NMR** (300 MHz, $CDCl_3$) δ = 8.40 – 7.60 (m, 27H), 5.44 – 5.32 (tt, *J* = 6.1, 4.1 Hz, 1H), 4.47 – 4.31 (dd, *J* = 12.0, 4.1 Hz, 2H), 4.29 – 4.14 (dd, *J* = 12.0, 6.1 Hz, 2H), 3.43 – 3.16 (m, 6H), 2.54 – 2.33 (m, 6H), 2.29 – 2.01 (m, 6H) ppm. **HRMS** (ESI+): calc'd for $C_{63}H_{50}O_6$ [M$^+$] 902.3602, found 902.3606, calc'd for $C_{63}H_{50}O_6Na$ [M+Na$^+$] 925.3500, found 925.3506, calc'd for $C_{63}H_{50}O_6K$ [M+K$^+$] 941.3239, found 941.3245.

Synthesis of Py$_4$-Erythritol. In a 20 mL scintillation vial equipped with a stir bar, PyBA (0.30 g, 1.05 mmol, 5.0 eq), HOBt·H_2O (24.6 mg, 0.16 mmol, 0.8 eq), and *meso*-erythritol (25.7 mg, 0.21 mmol, 1.0 eq) were added to 10 mL of distilled DCM. The mixture was stirred to form a brown

dispersion which persisted after sonication. After 5 minutes, DIPEA (0.55 mL, 3.16 mmol, 15.0 eq) was added which led to the dissolution of most, but not all, of the precipitate. After waiting 10 minutes, EDC·HCl (0.31 g, 1.59 mmol, 7.6 eq) was added to the reaction mixture before leaving it to stir overnight. The following day, the precipitate was removed from the reaction mixture by vacuum filtration (Whatman # 5). The filtrate was transferred to a 125 mL separatory funnel with 40 mL of DCM and washed with 10 mL of DI H_2O. The organic layer was dried over sodium sulfate (Na_2SO_4) and filtered through celite before condensing to 2 mL for column chromatography. A silica gel column was run with a solvent gradient from toluene to 97:3 toluene:acetone before recombining the product-containing fractions and precipitating from 80 mL of cold hexanes. The product was then isolated via vacuum filtration (Whatman #5) before drying in a vacuum oven at room temperature overnight to yield a white cakey solid (7.0 mg, 3 %). **^{1}H NMR** (300 MHz, $CDCl_3$) δ = 8.38 – 7.57 (m, 36H), 5.51 – 5.33 (m, 2H), 4.55 – 4.35 (m, 2H), 4.34 – 4.17 (m, 2H), 3.43 – 3.13 (br. t, *J* = 7.6 Hz, 8H), 2.55 – 2.34 (m, 8H), 2.25 – 1.99 (m, 8H) ppm. **HRMS** (ESI+): calc'd for $C_{84}H_{66}O_8$ [M^+] 1202.4752, found 1202.4781, calc'd for $C_{84}H_{66}O_8Na$ [$M+Na^+$] 1225.4650, found 1225.4681, calc'd for $C_{84}H_{66}O_8K$ [$M+K^+$] 1241.4389, found 1241.4422.

Synthesis of Py_5-Adonitol. This synthesis was carried out by applying the general procedure but with a smaller excess of PyBA, base, and coupling agents (PyBA:ROH:HOBt·H_2O:DIPEA:EDC·HCl = 1.25:1:0.19:1.87:1.87) to facilitate simpler removal of free pyrene with PyBA (0.30 g, 1.05 mmol), adonitol (24.7 mg, 0.16 mmol, 1.0 eq), HOBt·H_2O (24.0 mg, 0.16 mmol), DIPEA (0.2 mL, 1.15 mmol), and EDC·HCl (0.20 g, 1.16 mmol) and using a gradient from toluene to 19:1 toluene:acetone as column eluent. The product was obtained as a white cakey solid (1.4 mg, 1 %). **^{1}H NMR** (300 MHz, $CDCl_3$) δ = 8.25 – 7.60 (m, 45H), 5.63 – 5.53 (t, *J* = 5.6 Hz, 1H), 5.52 – 5.41 (ddd, *J* = 6.1, 6.1, 2.8 Hz, 2H), 4.58 – 4.38 (dd, *J* = 12.1, 3.0 Hz, 2H), 4.34 – 4.15 (dd, *J* = 6.4, 12.2 Hz, 2H), 3.38 – 3.07 (m, 10H), 2.61 – 2.29 (m, 10H), 2.24 – 1.97 (m, 10H) ppm. **HRMS** (ESI+): calc'd for $C_{105}H_{82}O_{10}$ [M^+] 1502.5903, found 1502.5957, calc'd for $C_{84}H_{66}O_8Na$ [$M+Na^+$] 1525.5800, found 1525.5843.

Synthesis of Py_6-Sorbitol. In a 20 mL scintillation vial equipped with a stir bar, PyBA (0.31 g, 1.07 mmol, 8.6 eq), HOBt·H_2O (23.0 mg, 0.15 mmol, 1.2 eq), and D-sorbitol (22.6 mg, 0.12 mmol, 1.0 eq) were added to 10 mL of distilled DCM to form a brown/orange dispersion. After 5 minutes,

DIPEA (0.55 mL, 3.16 mmol, 25.5 eq) was added which led to the dissolution of most of the dispersed material. After another 15 minutes, EDC·HCl (0.29 g, 1.51 mmol, 12.2 eq) was added to the reaction mixture which again formed a dispersion and was left to stir overnight. The next day the solution was run through a pipette of celite to remove precipitates before adding 4 mL of DI H_2O and 10 mg of NaCl to the DCM filtrate to stir and extract residual byproducts. The organic layer was then removed via pipette and dried over Na_2SO_4 before being condensed for column chromatography. Silica gel chromatography was used with an eluent gradient from toluene to 97:3 toluene:acetone to separate the product before combining the relevant fractions for precipitation from 80 mL of ice-cold hexanes. The solids were then isolated via vacuum filtration (Whatman #5) and dried in a vacuum oven overnight at room temperature to yield the product as a white cakey solid (4.0 mg, 2 %). **^{1}H NMR** (300 MHz, $CDCl_3$) δ = 8.25 – 7.52 (m, 54H), 5.71 – 5.57 (br. d, *J* = 6.4 Hz, 2H), 5.53 – 5.35 (m, 1H), 5.32 – 5.17 (m, 1H), 4.61 – 4.48 (dd, *J* = 12.1, 3.3 Hz, 1H), 4.45 – 4.32 (dd, *J* = 12.2, 3.3 Hz, 1H), 4.28 – 4.07 (m, 2H), 3.37 – 3.01 (m, 12H), 2.58 – 2.24 (m, 12H), 2.21 – 1.84 (m, 12H) ppm. **HRMS** (ESI+): calc'd for $C_{126}H_{98}O_{12}$ [M^+] 1802.7053, found 1802.7079, calc'd for $C_{126}H_{98}O_{12}Na$ [$M+Na^+$] 1825.6951, found 1825.6954.

B) ^{1}H NMR and COSY Spectra of the Py_2-DO and Py-PO samples

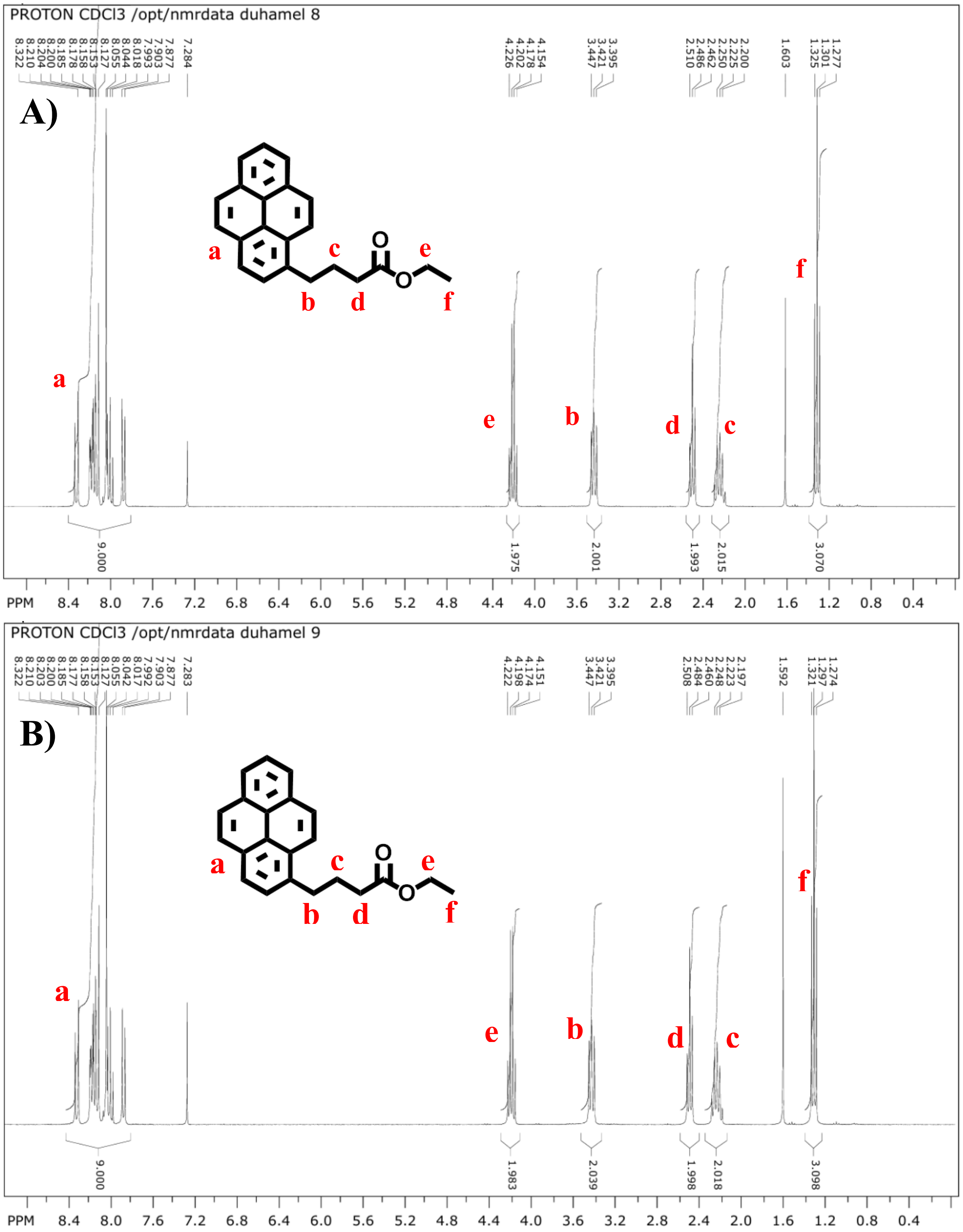

Figure S1. ^{1}H NMR spectrum of Py-BE in $CDCl_3$ obtained from either A) evaporation of the filtrate or B) precipitate. The peaks at 7.27 and 1.58 ppm are from $CHCl_3$ and H_2O, respectively.

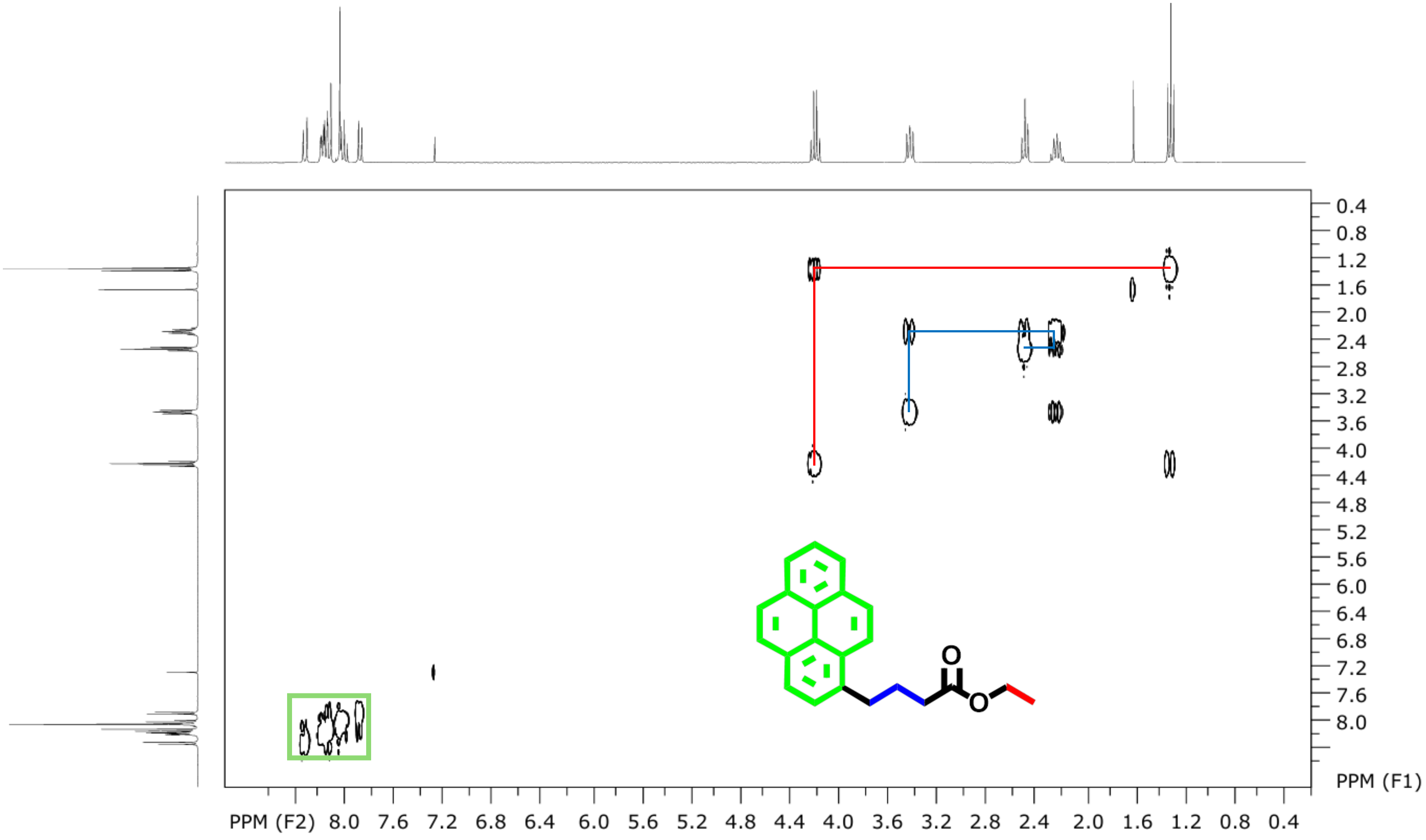

Figure S2. COSY spectrum of Py-BE obtained from precipitation, in $CDCl_3$.

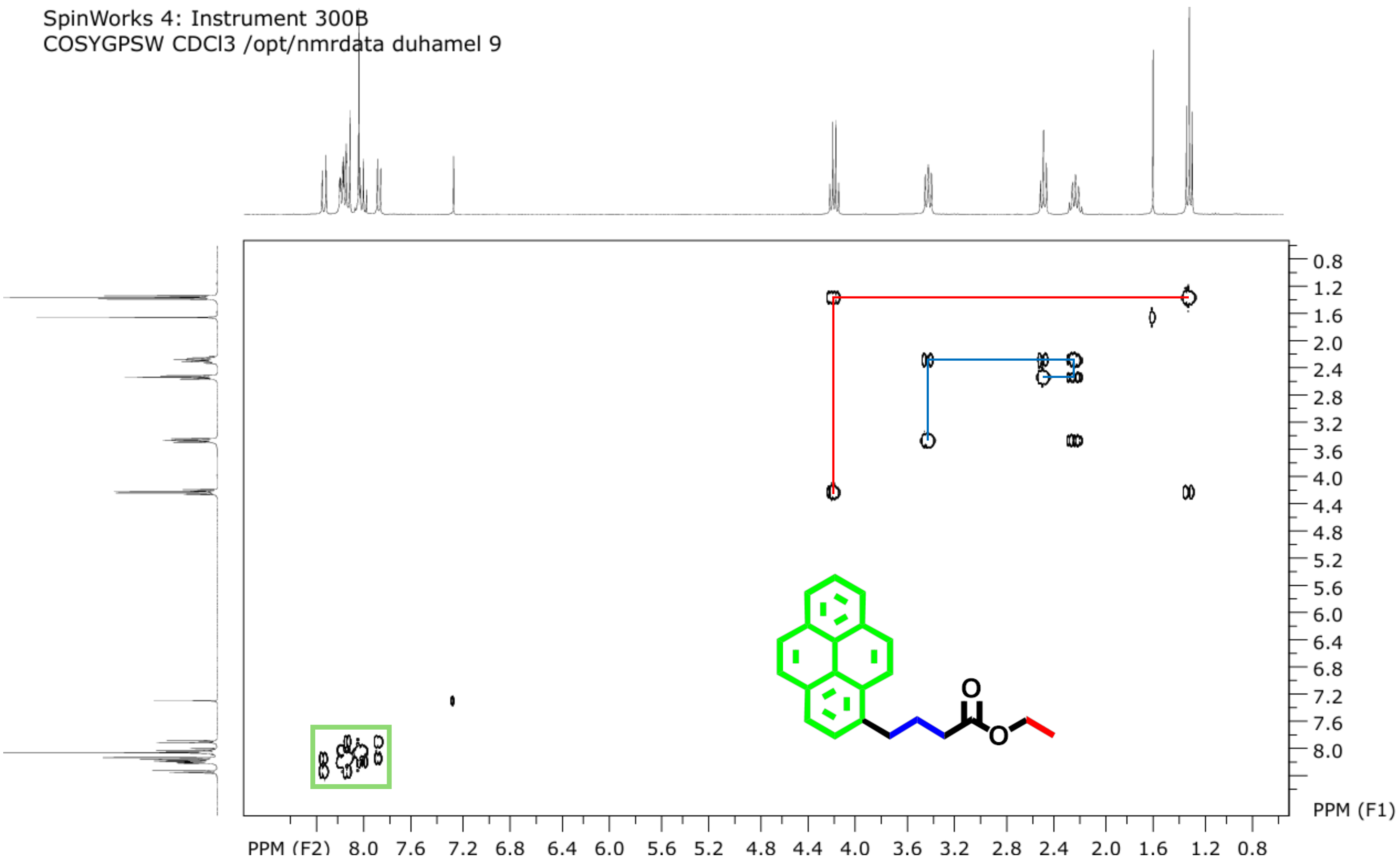

Figure S3. COSY spectrum of Py-BE obtained from evaporation of filtrate, in $CDCl_3$.

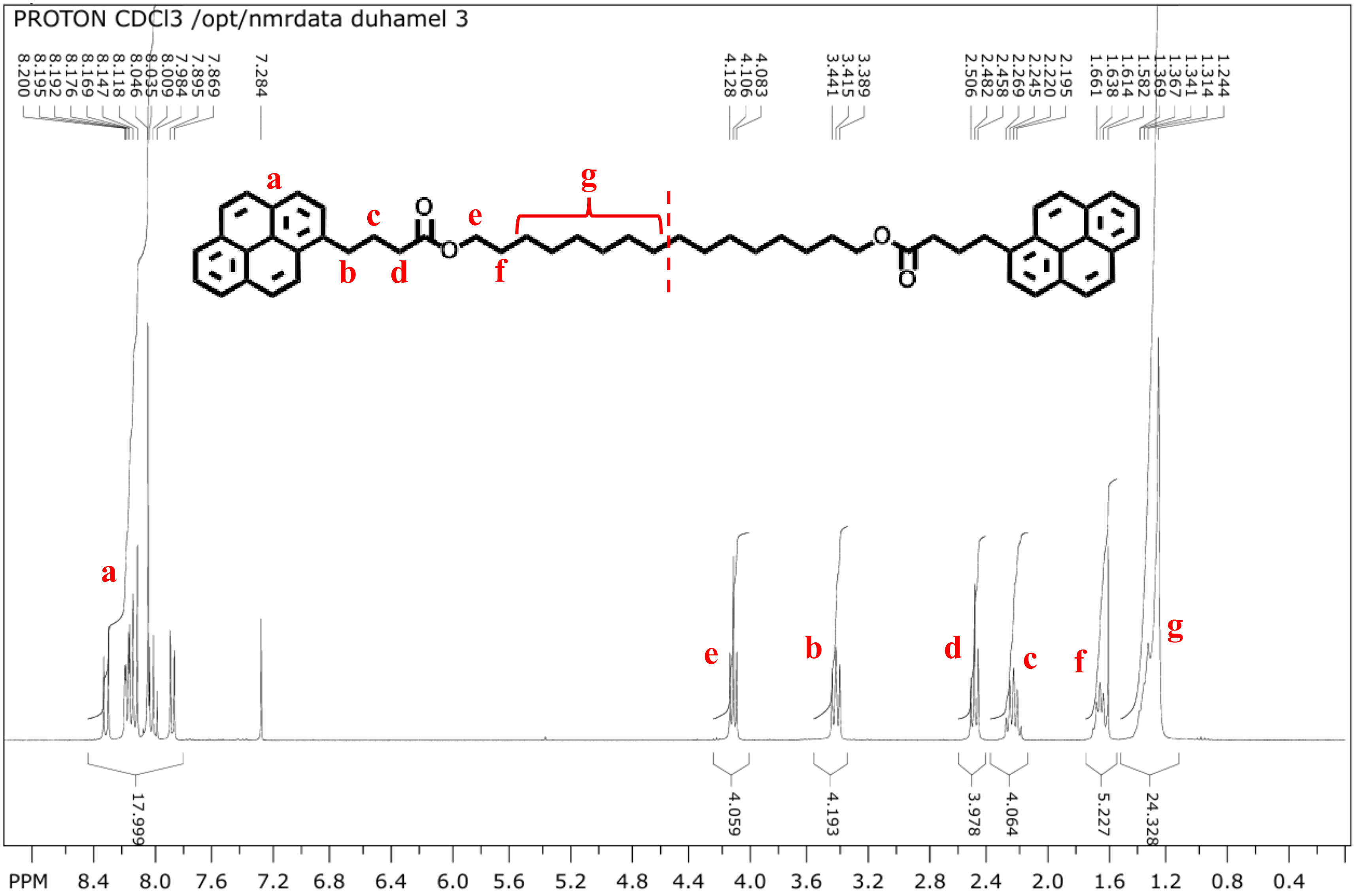

Figure S4. ^{1}H NMR spectrum of Py_2-HexadecDiol in $CDCl_3$. The peaks at 7.28 and 1.58 ppm are from $CHCl_3$ and H_2O, respectively.

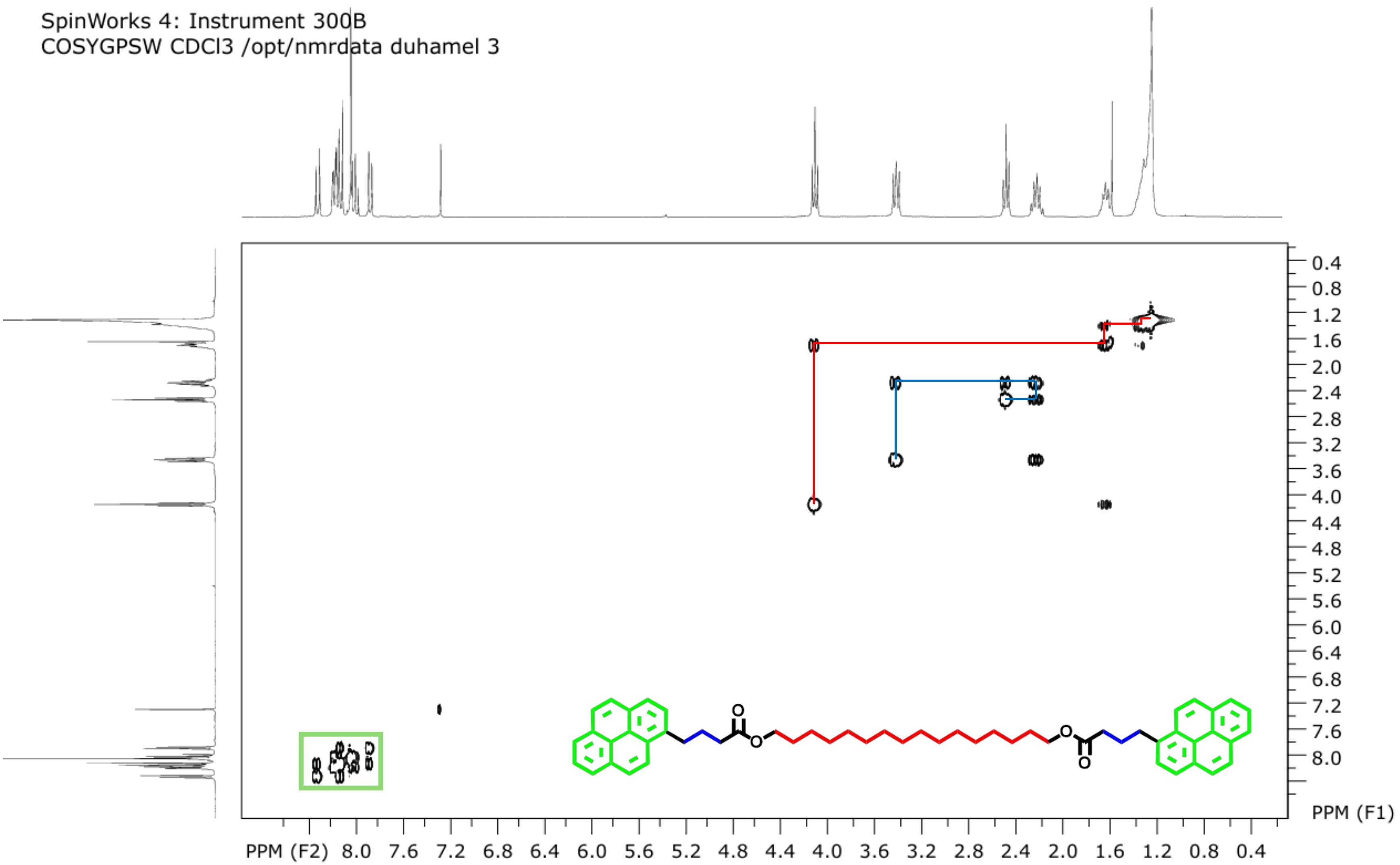

Figure S5. COSY spectrum of Py_2-HexadecDiol in $CDCl_3$.

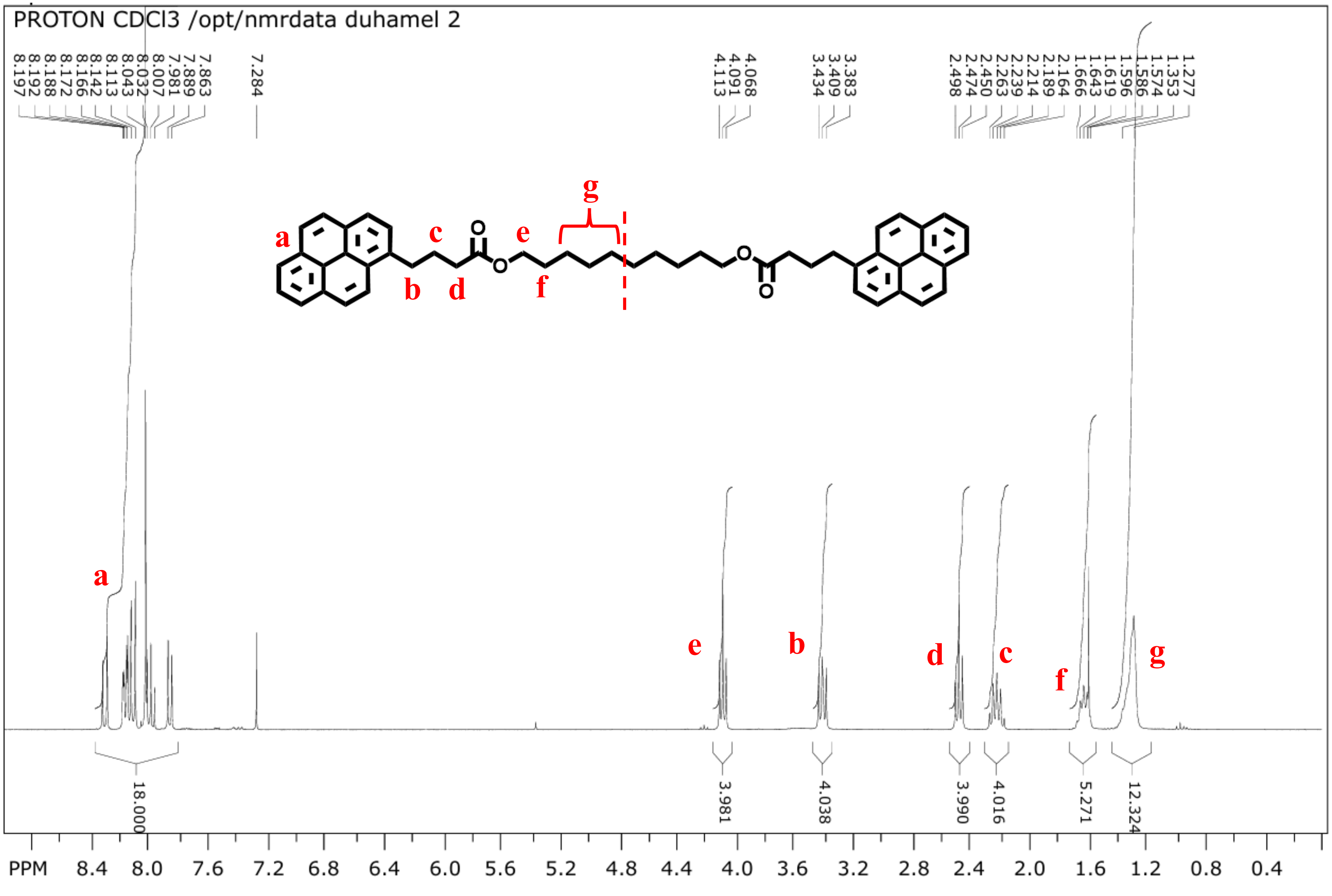

Figure S6. ^{1}H NMR spectrum of Py_2-DecDiol in $CDCl_3$. The peaks at 7.28 and 1.57 ppm are from $CHCl_3$ and H_2O, respectively.

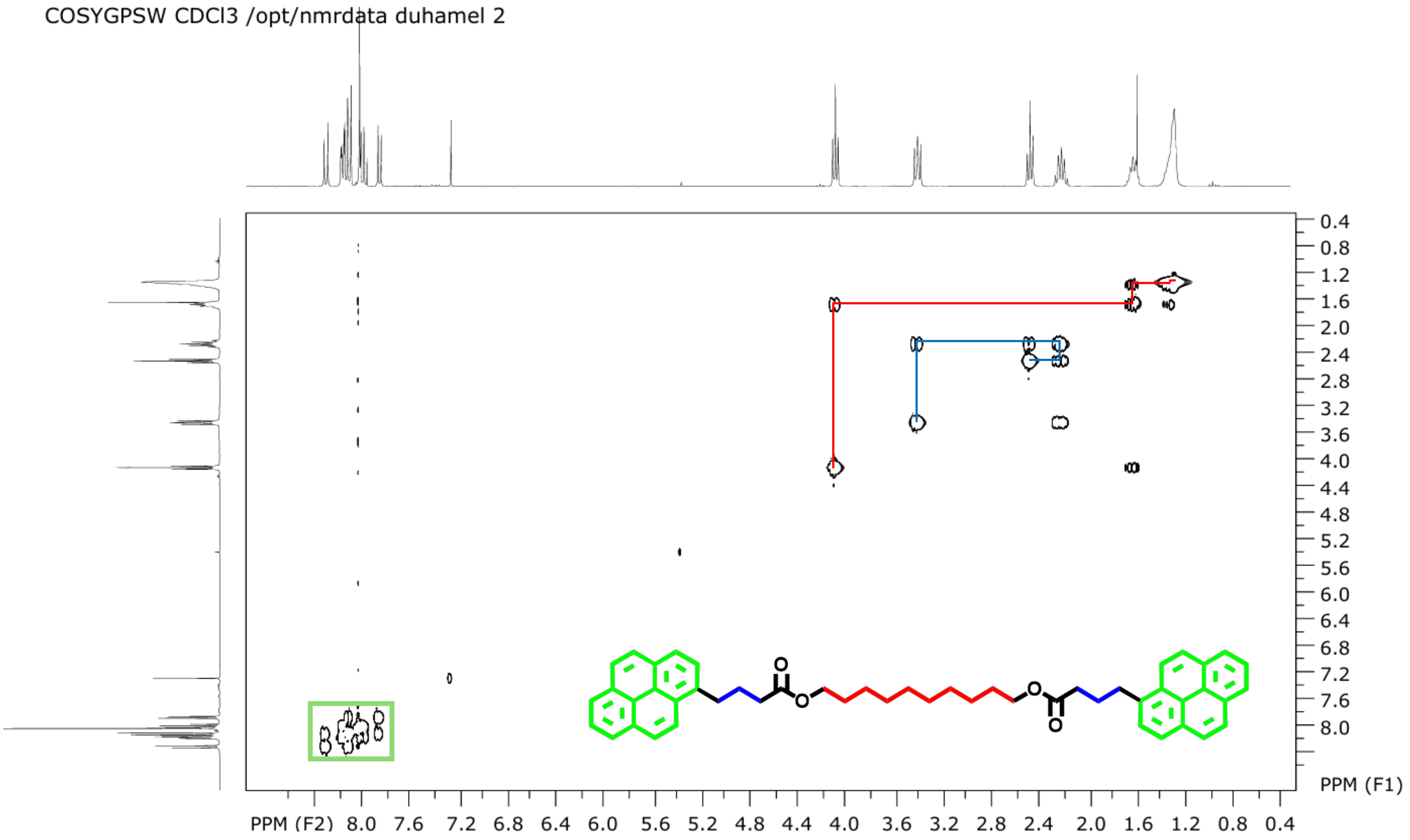

Figure S7. COSY spectrum of Py_2-DecDiol in $CDCl_3$.

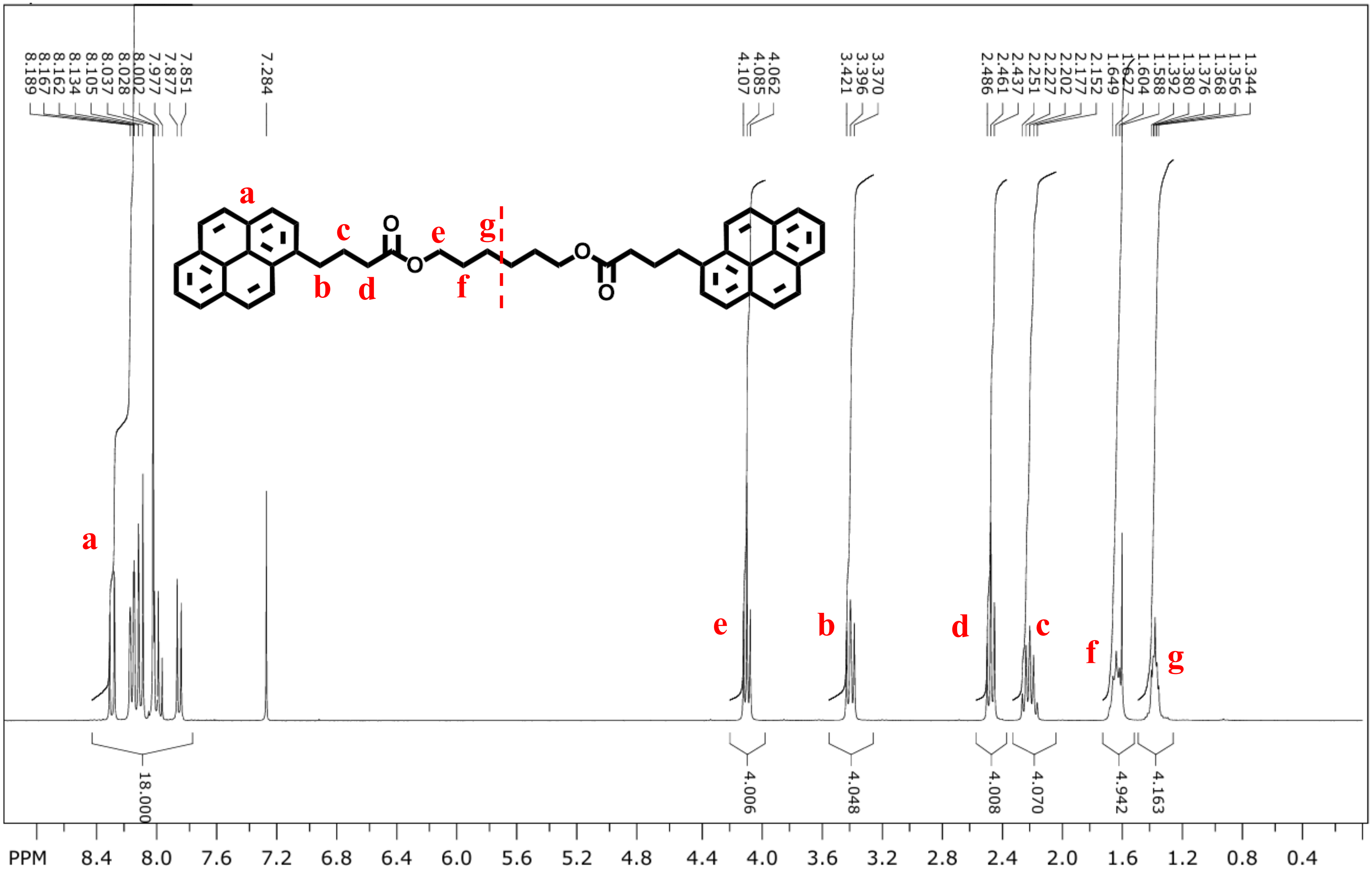

Figure S8. ^{1}H NMR spectrum of Py_2-HexDiol in $CDCl_3$. The peaks at 7.28 and 1.59 ppm are from $CHCl_3$ and H_2O, respectively.

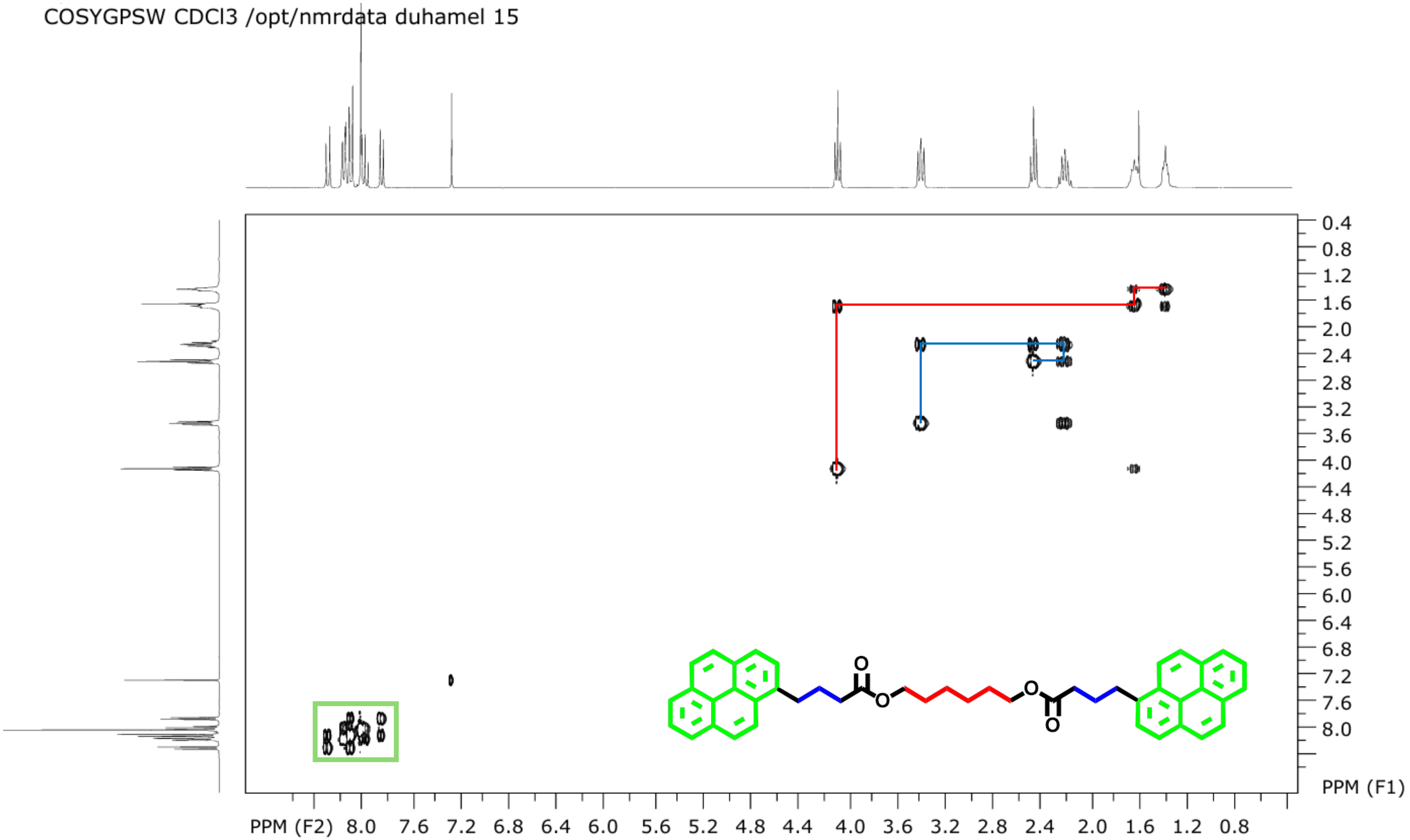

Figure S9. COSY spectrum of Py_2-HexDiol in $CDCl_3$.

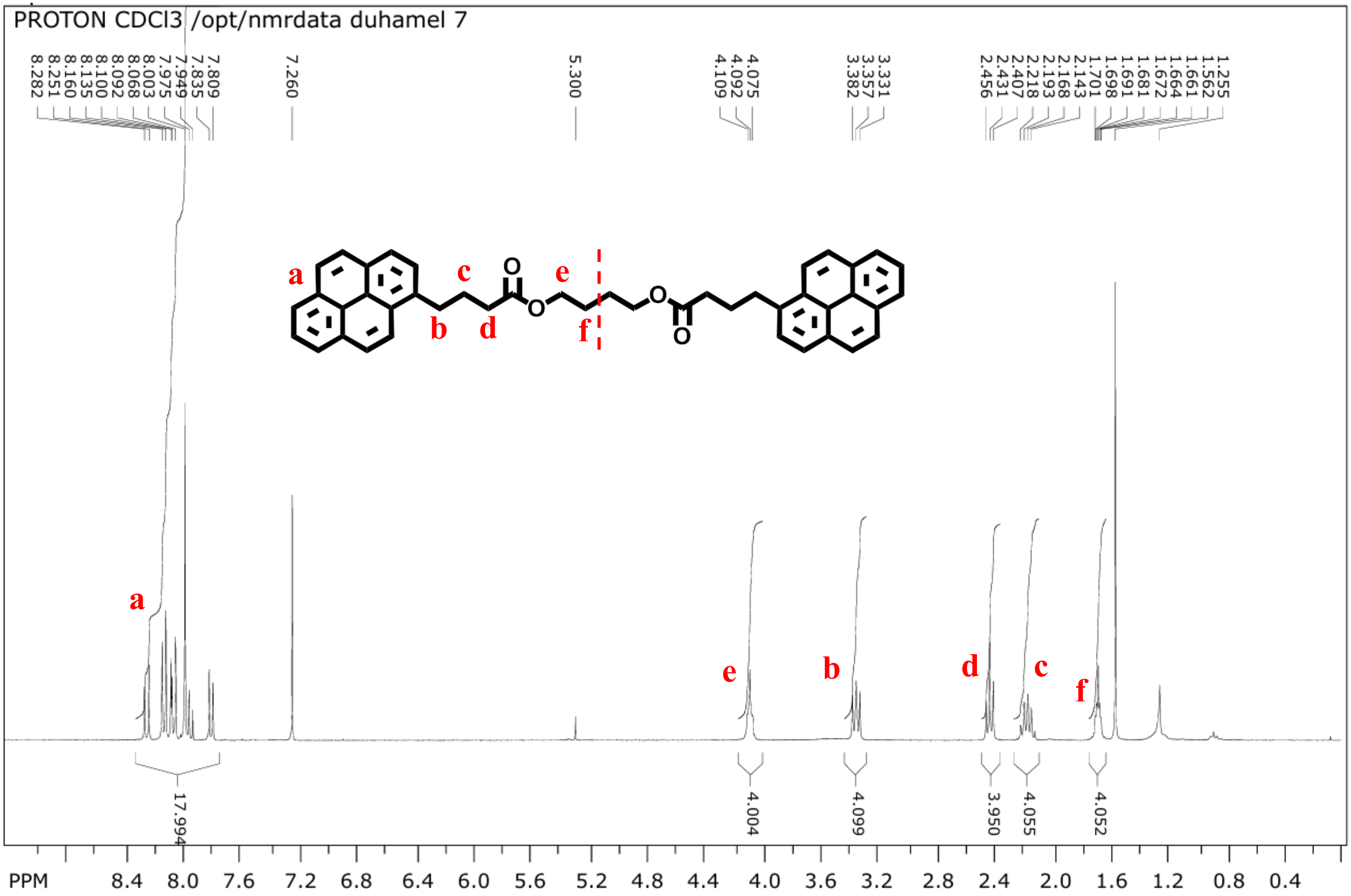

Figure S10. ^{1}H NMR spectrum of Py_2-ButDiol in $CDCl_3$. The peaks at 7.26 and 1.56 ppm are from $CHCl_3$ and H_2O, respectively. Peaks at ~0.8 and ~1.2 ppm are from hexane grease.

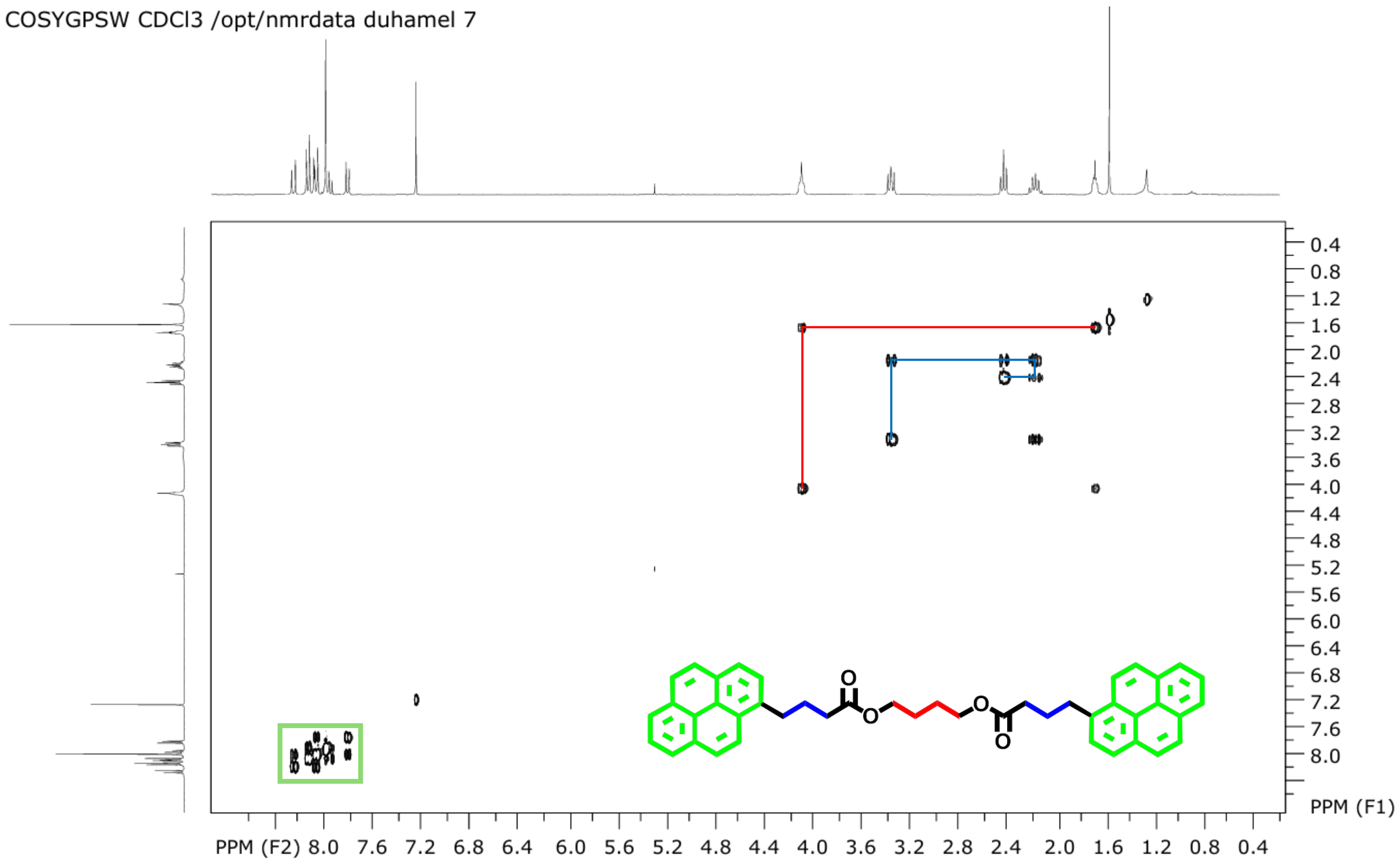

Figure S11. COSY spectrum of Py_2-ButDiol in $CDCl_3$.

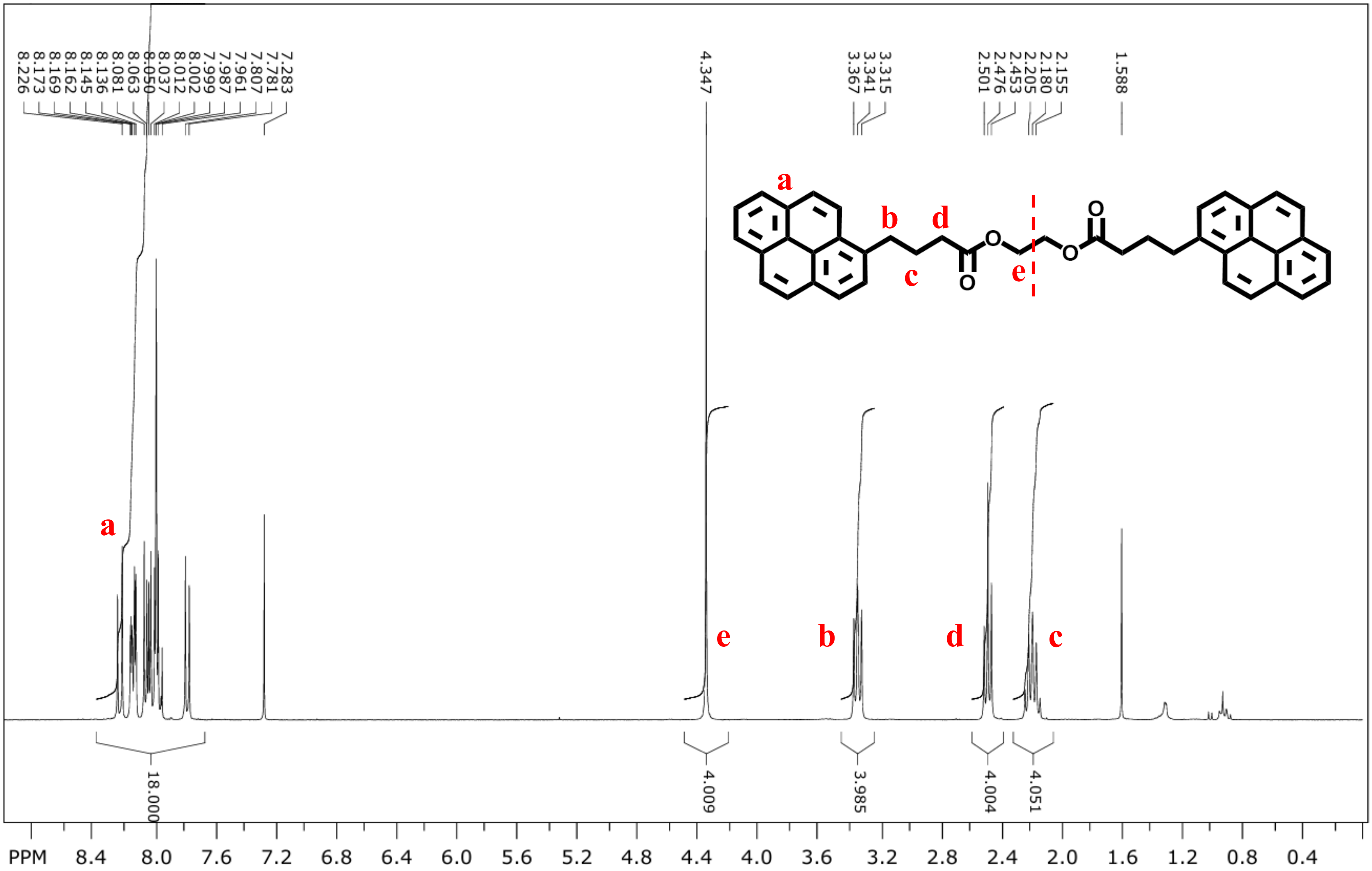

Figure S12. ^{1}H NMR spectrum of Py_2-EG in $CDCl_3$. The peaks at 7.28 and 1.59 ppm are from $CHCl_3$ and H_2O, respectively. Peaks at ~0.8 and ~1.2 ppm are from hexane grease.

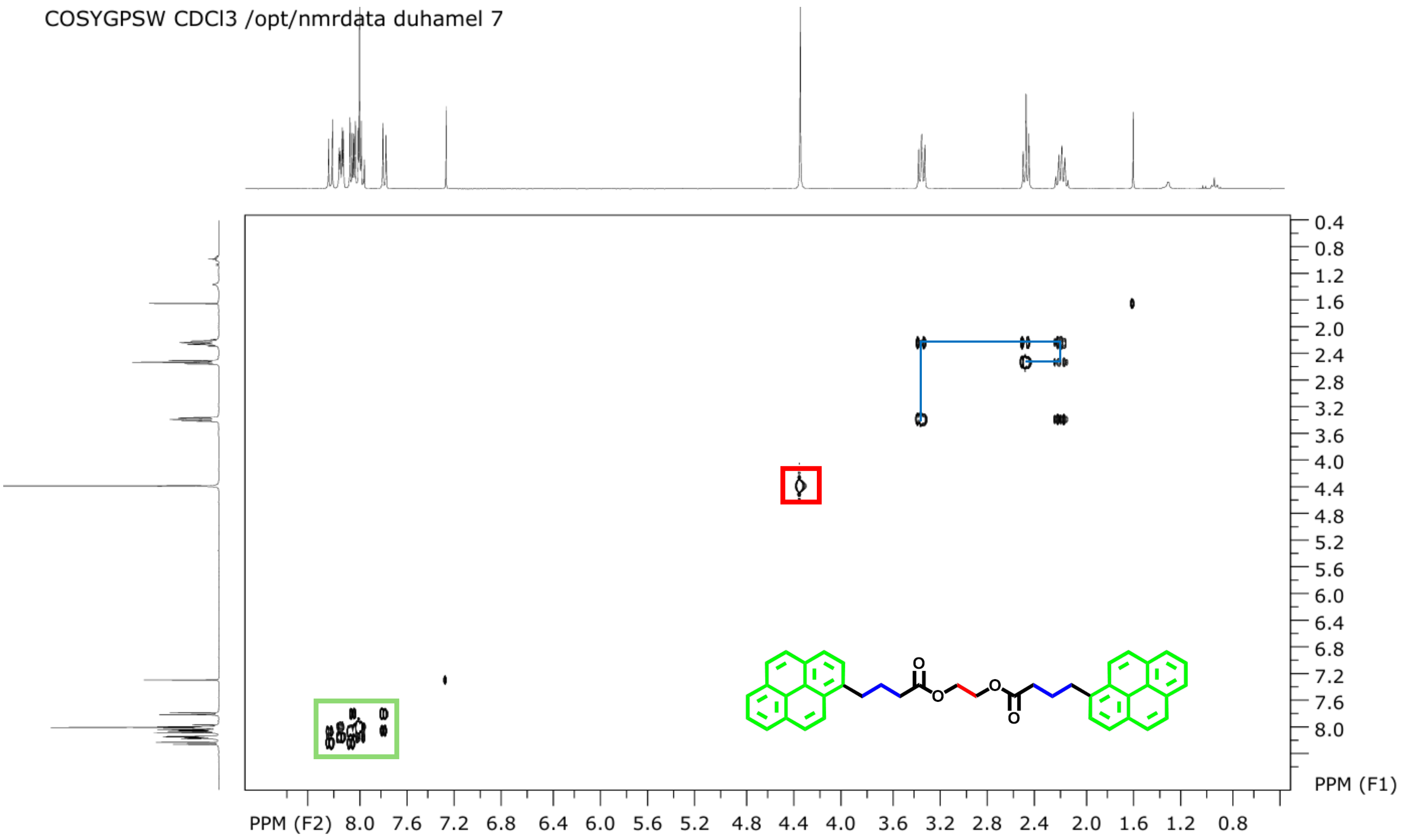

Figure S13. COSY spectrum of Py_2-EG in $CDCl_3$.

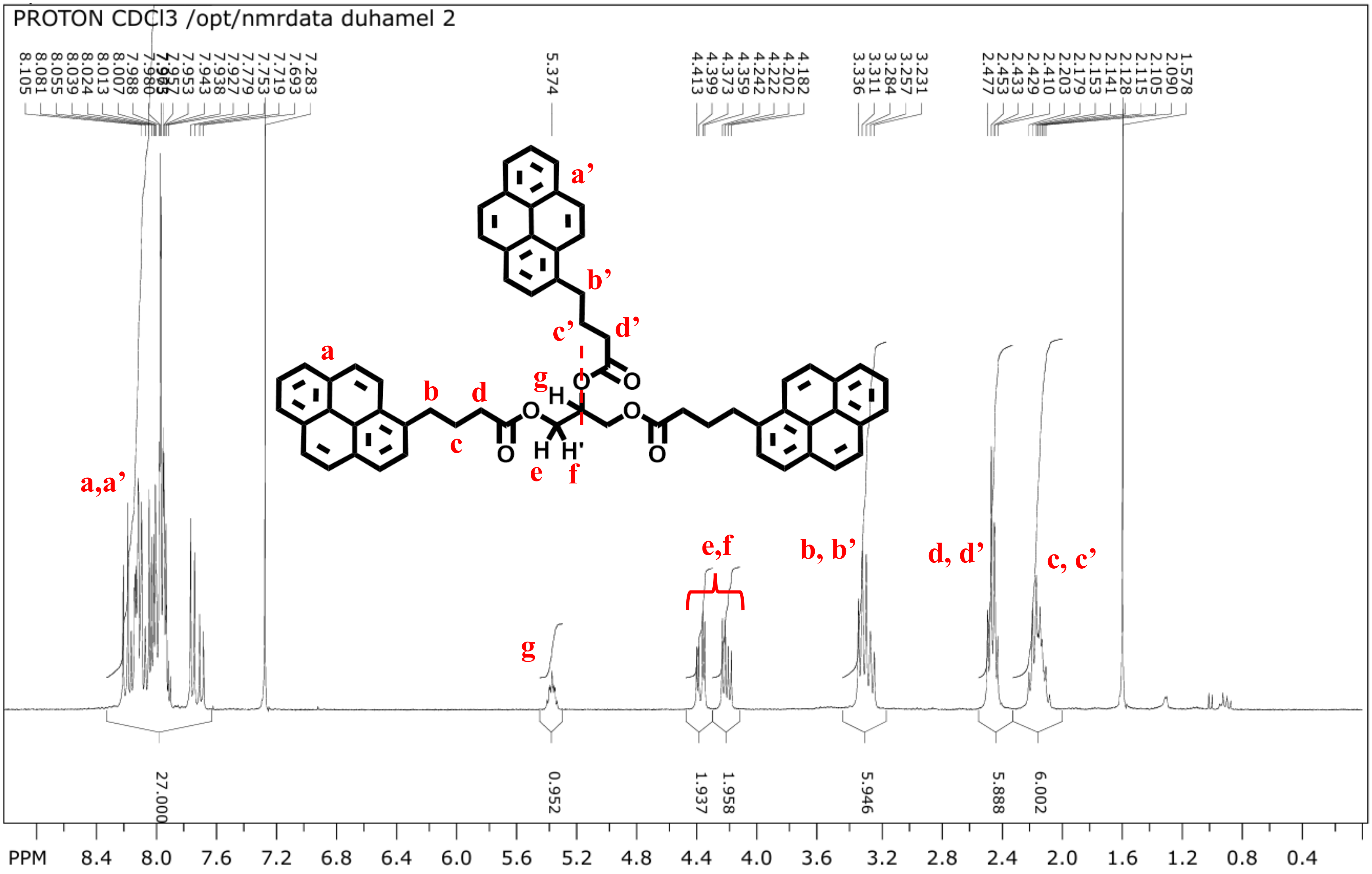

Figure S14. ^{1}H NMR spectrum of Py_3-Glycerol in $CDCl_3$. The peaks at 7.28 and 1.58 ppm are from $CHCl_3$ and H_2O, respectively. Peaks at ~0.8 and ~1.2 ppm are from hexane grease.

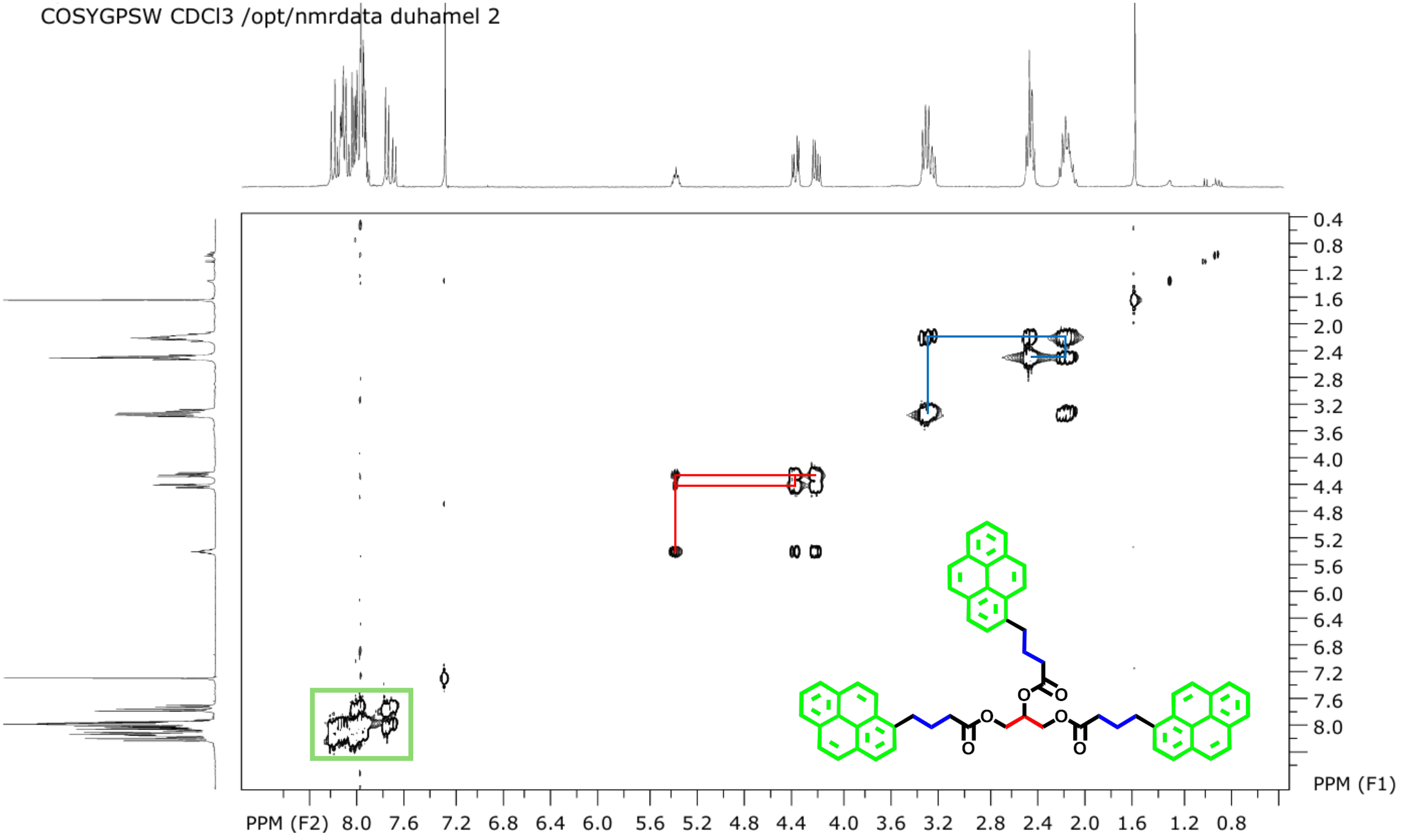

Figure S15. COSY spectrum of Py_3-Glycerol in $CDCl_3$.

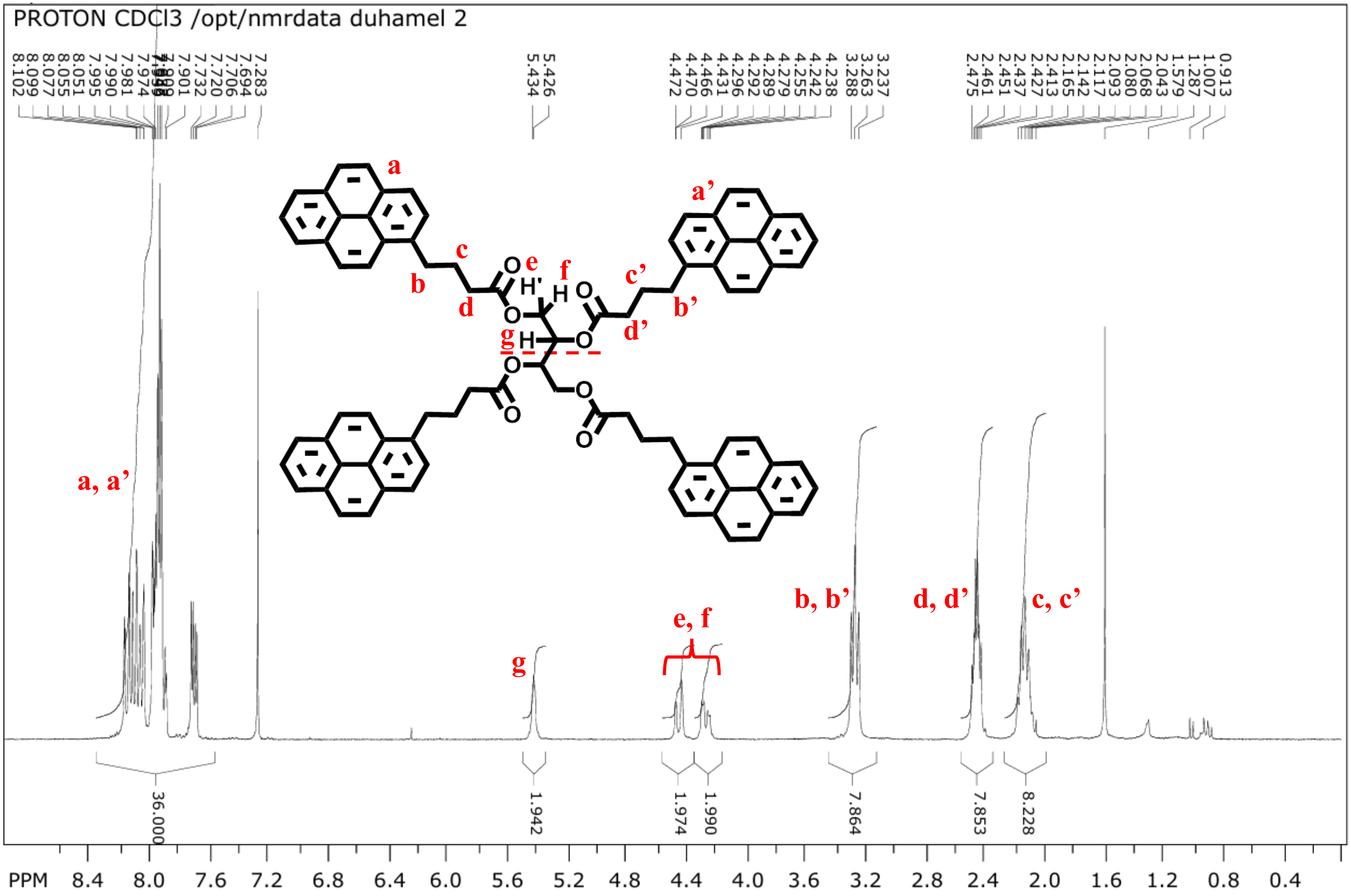

Figure S16. ^{1}H NMR spectrum of Py_4-Erythritol in $CDCl_3$. The peaks at 7.28 and 1.58 ppm are from $CHCl_3$ and H_2O, respectively. Peaks at ~0.8 and ~1.2 ppm are from hexane grease.

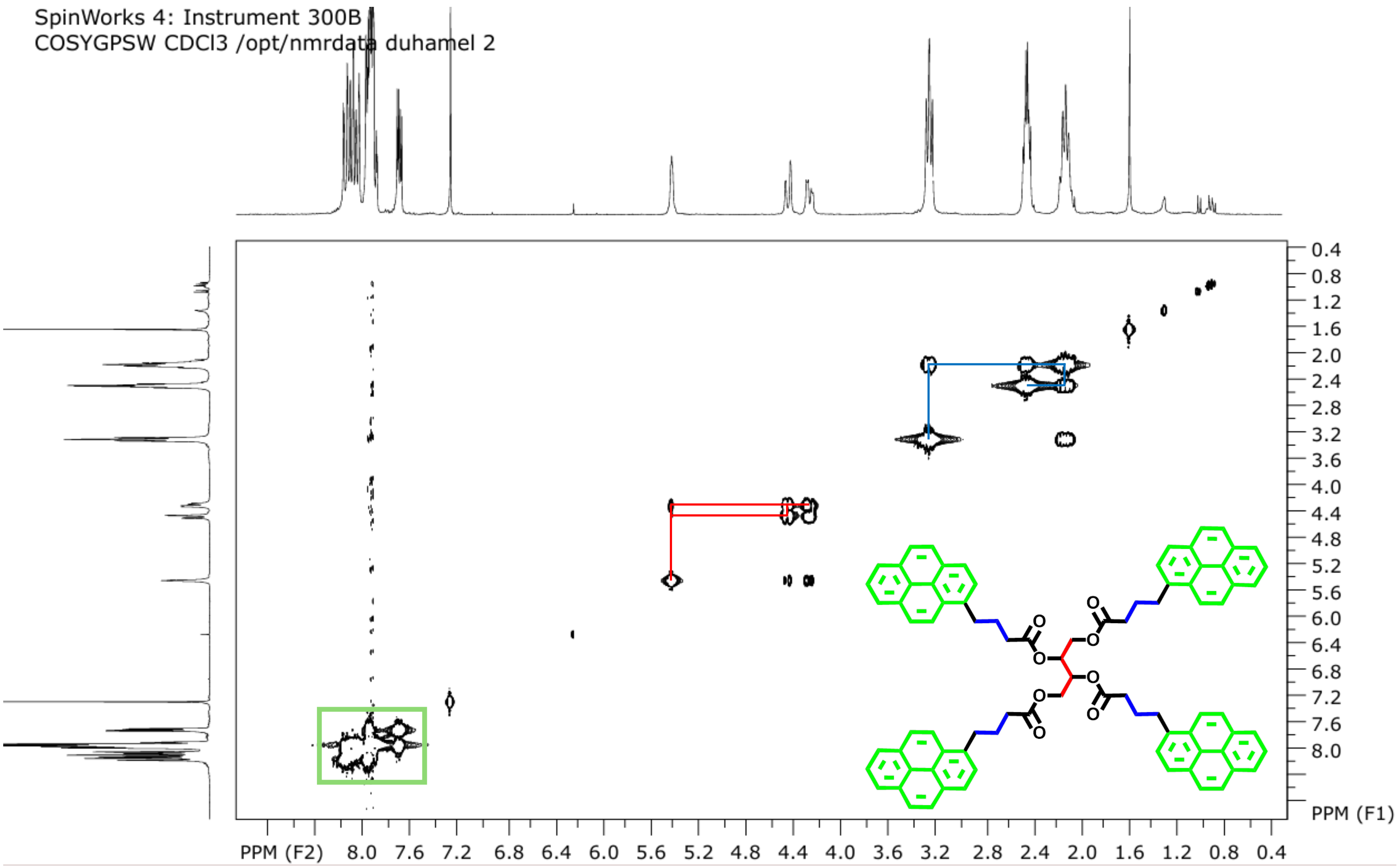

Figure S17. COSY spectrum of Py_4-Erythriol in $CDCl_3$.

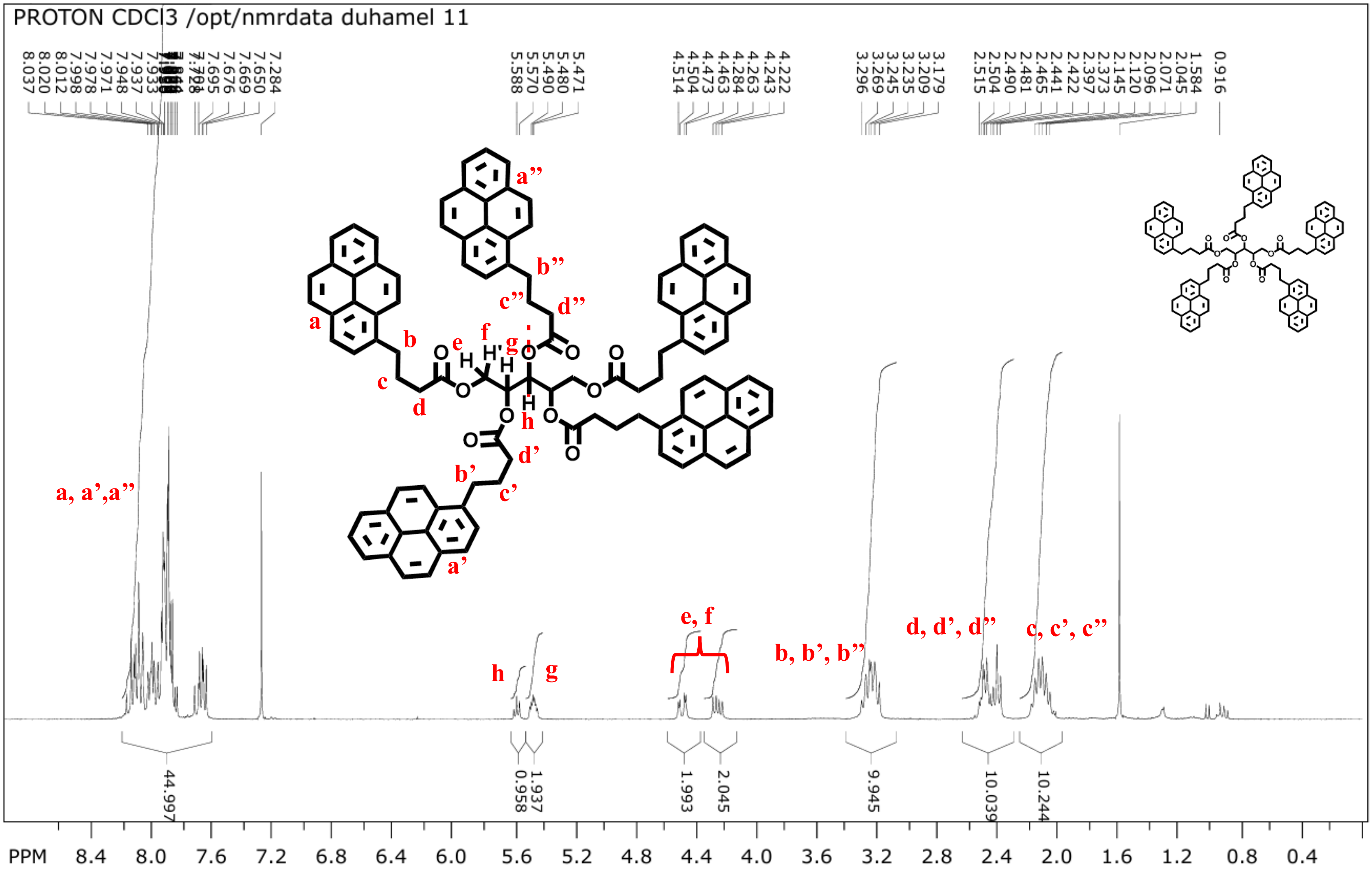

Figure S18. ^{1}H NMR spectrum of Py$_5$-Adonitol in $CDCl_3$. The peaks at 7.28 and 1.58 ppm are from $CHCl_3$ and H_2O, respectively. Peaks at ~0.8 and ~1.2 ppm are from hexane grease.

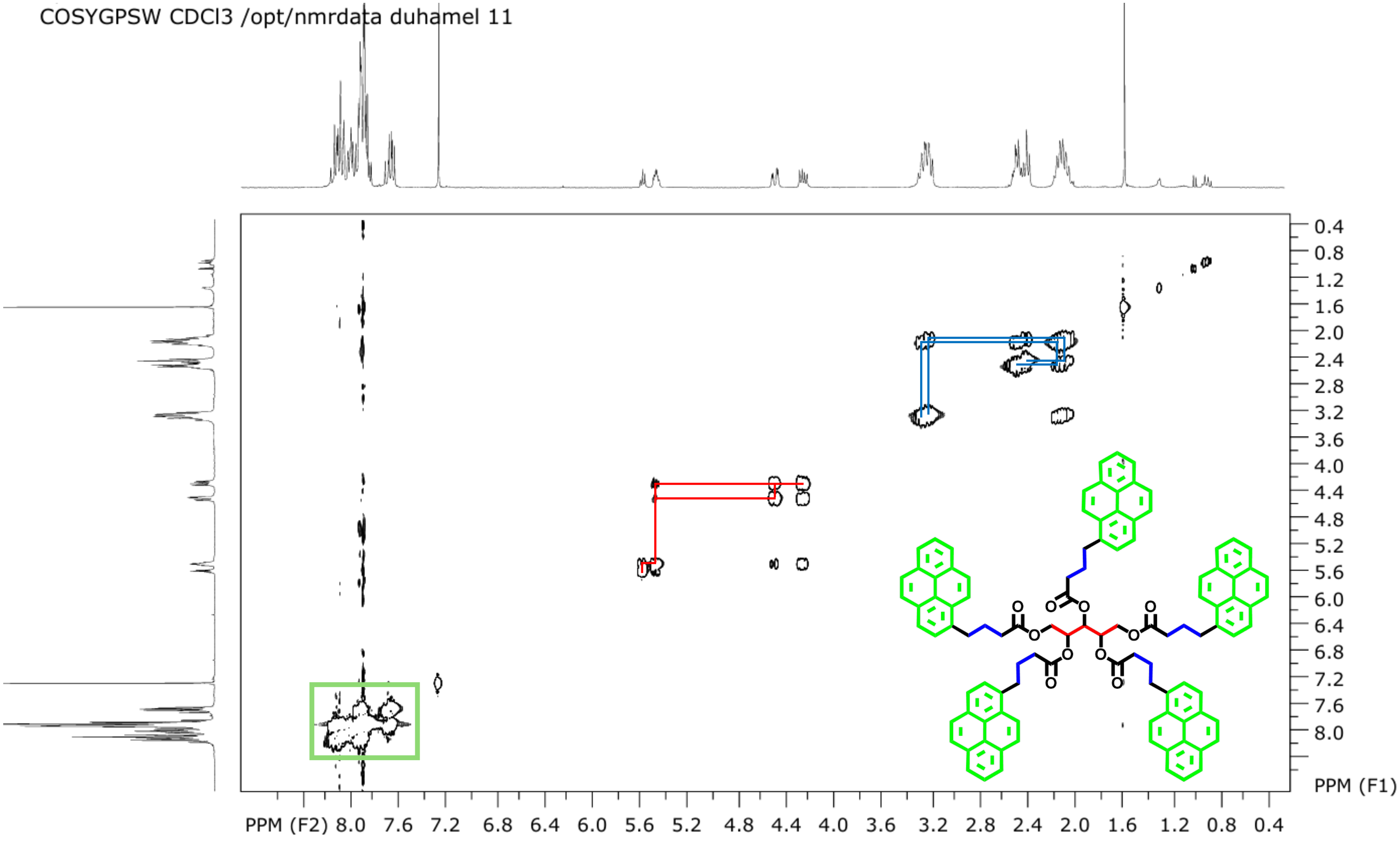

Figure S19. COSY spectrum of Py_5-Adonitol in $CDCl_3$.

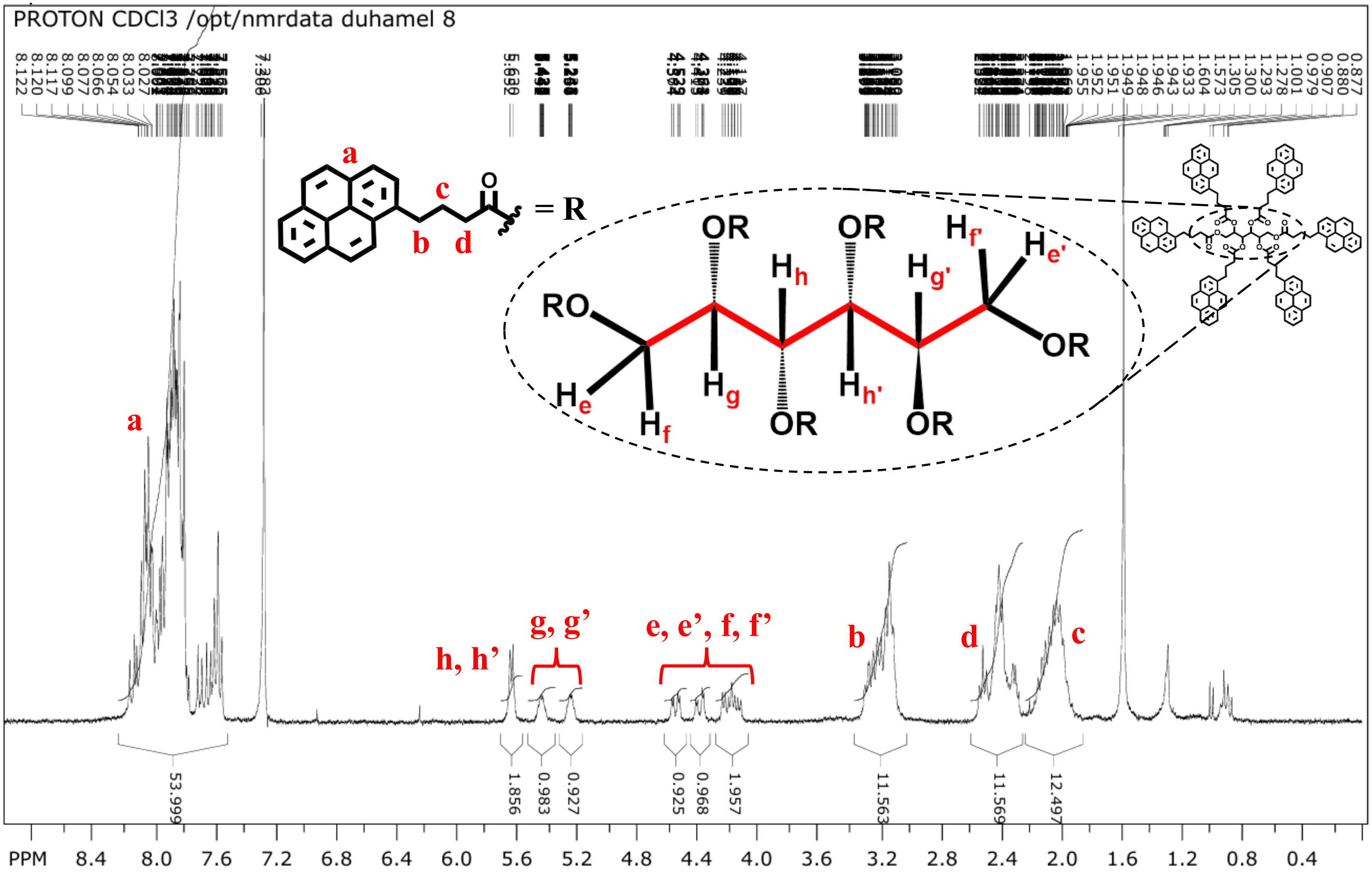

Figure S20. ^{1}H NMR spectrum of Py_6-Sorbitol in $CDCl_3$. The peaks at 7.28 and 1.57 ppm are from $CHCl_3$ and H_2O, respectively. Peaks at ~0.8 and ~1.2 ppm are from hexane grease.

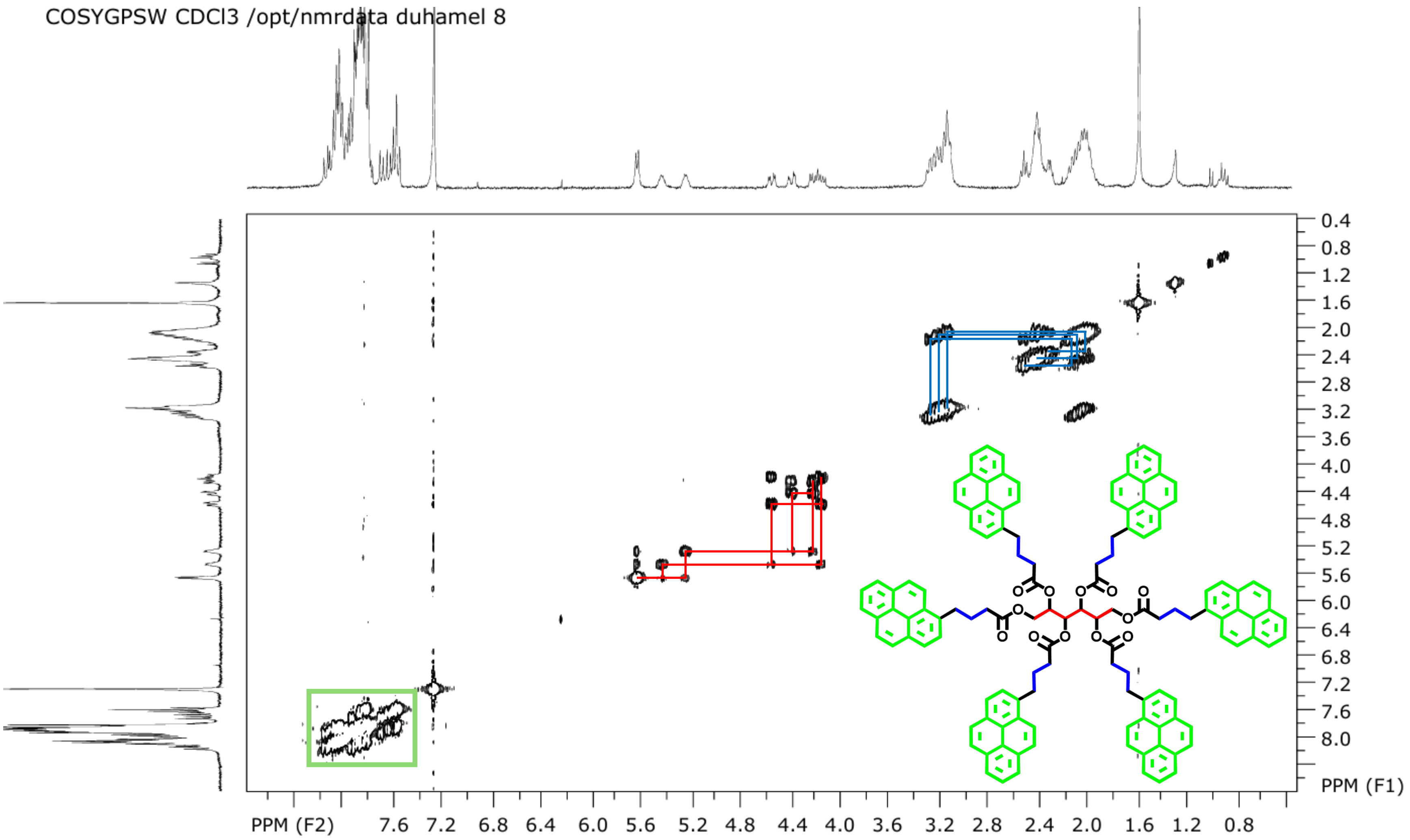

Figure S21. COSY spectrum of Py_6-Sorbitol in $CDCl_3$.

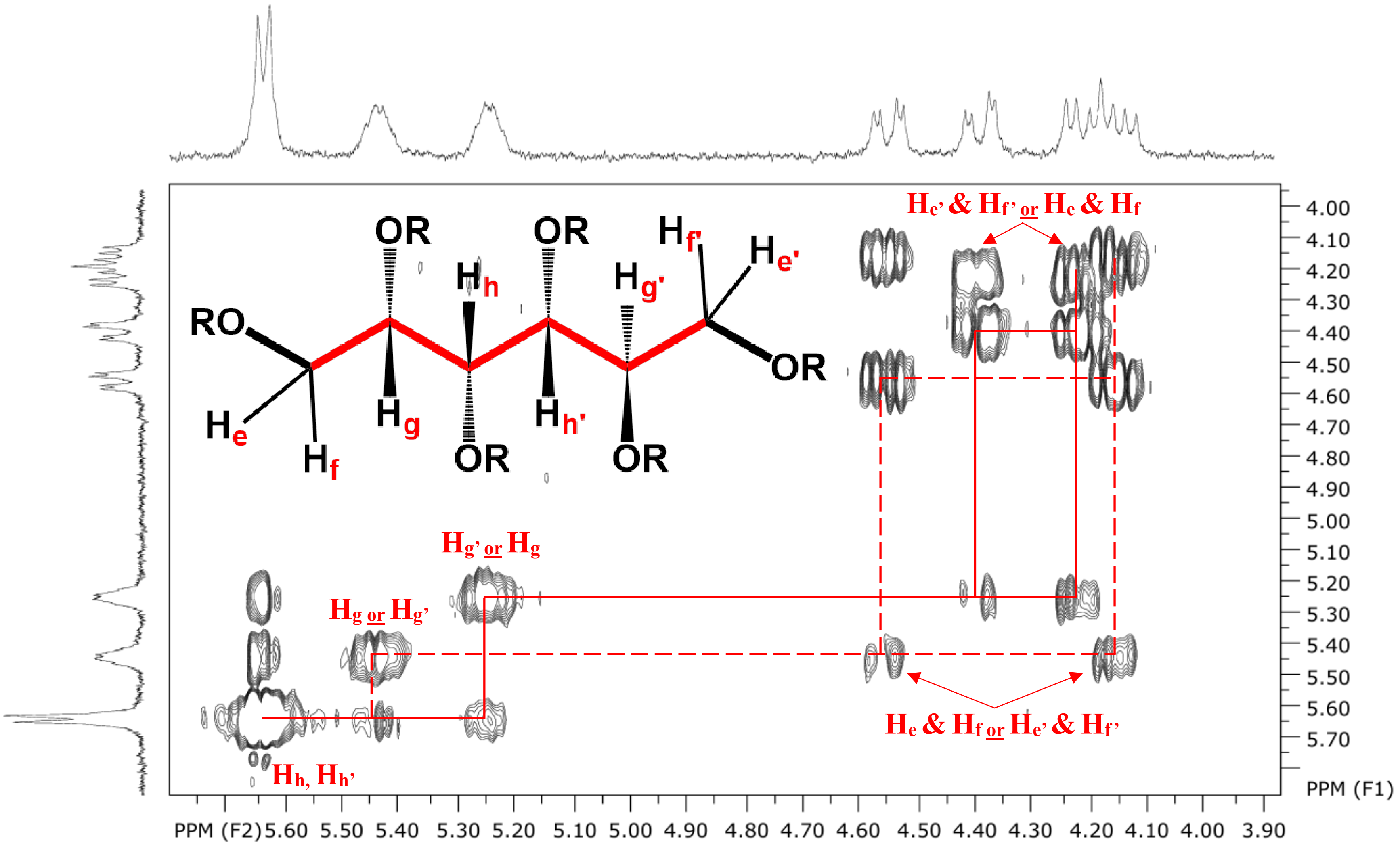

Figure S22. Zoomed in **COSY** spectrum of the core methine region of Py_6-Sorbitol in $CDCl_3$.

C) MS^n Fragmentation of the Py_2-DO and Py-PO samples

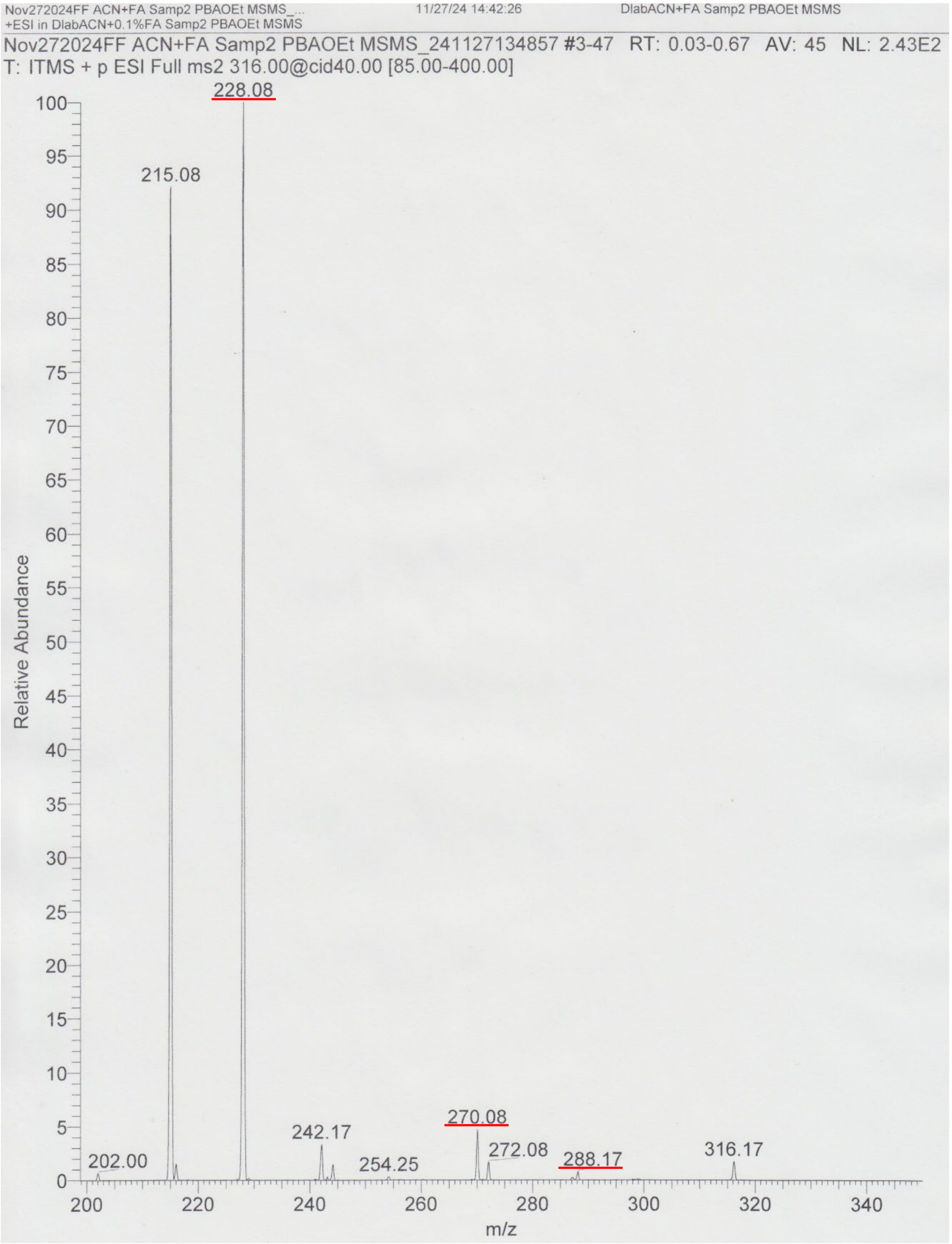

Figure S23. MS/MS Spectrum of PyBE with a selected precursor ion of 316.0 and common Py-PO fragments highlighted.

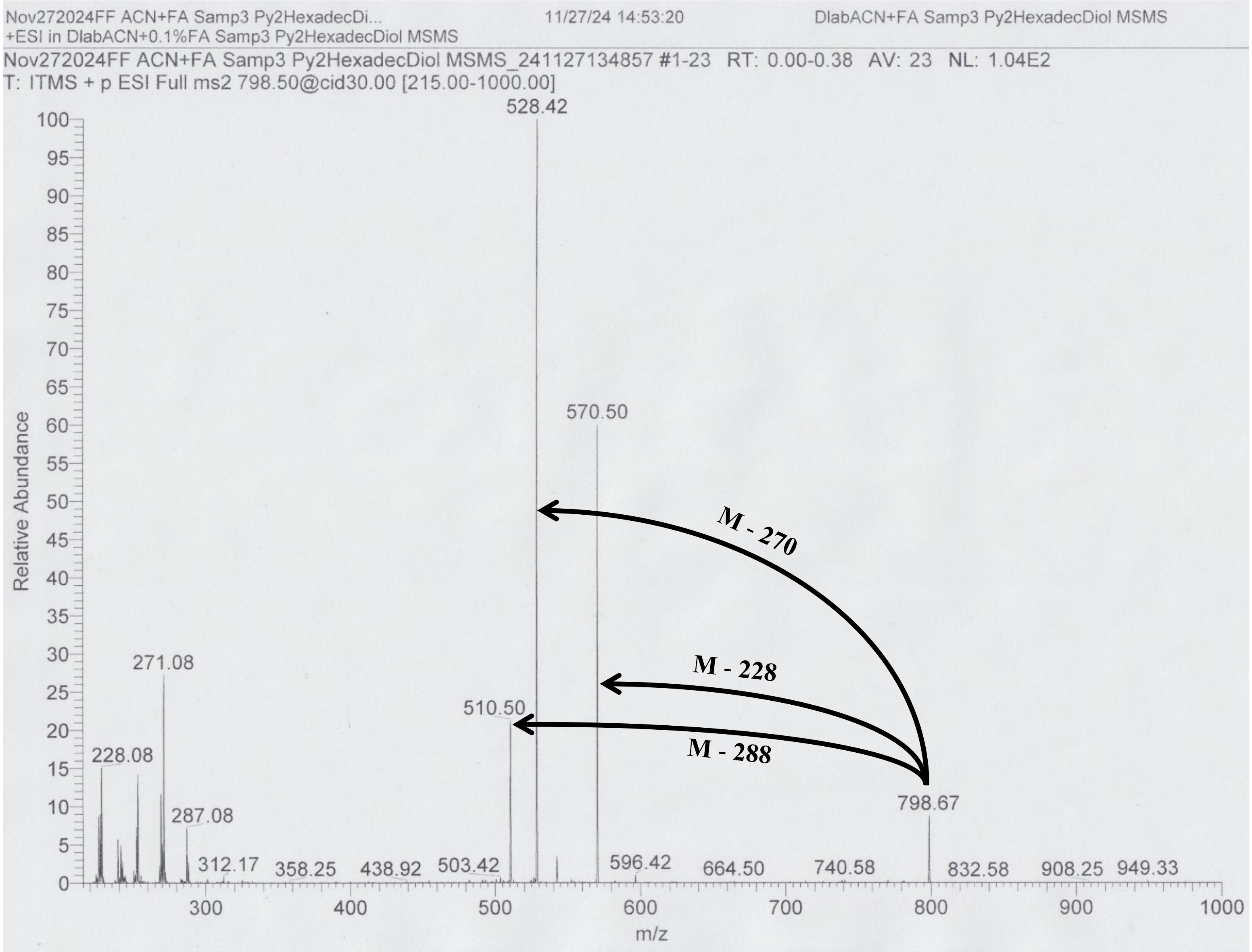

Figure S24. MS/MS Spectrum of Py_2-HexadecDiol with a selected precursor ion of 798.5 and common Py-PO fragments indicated.

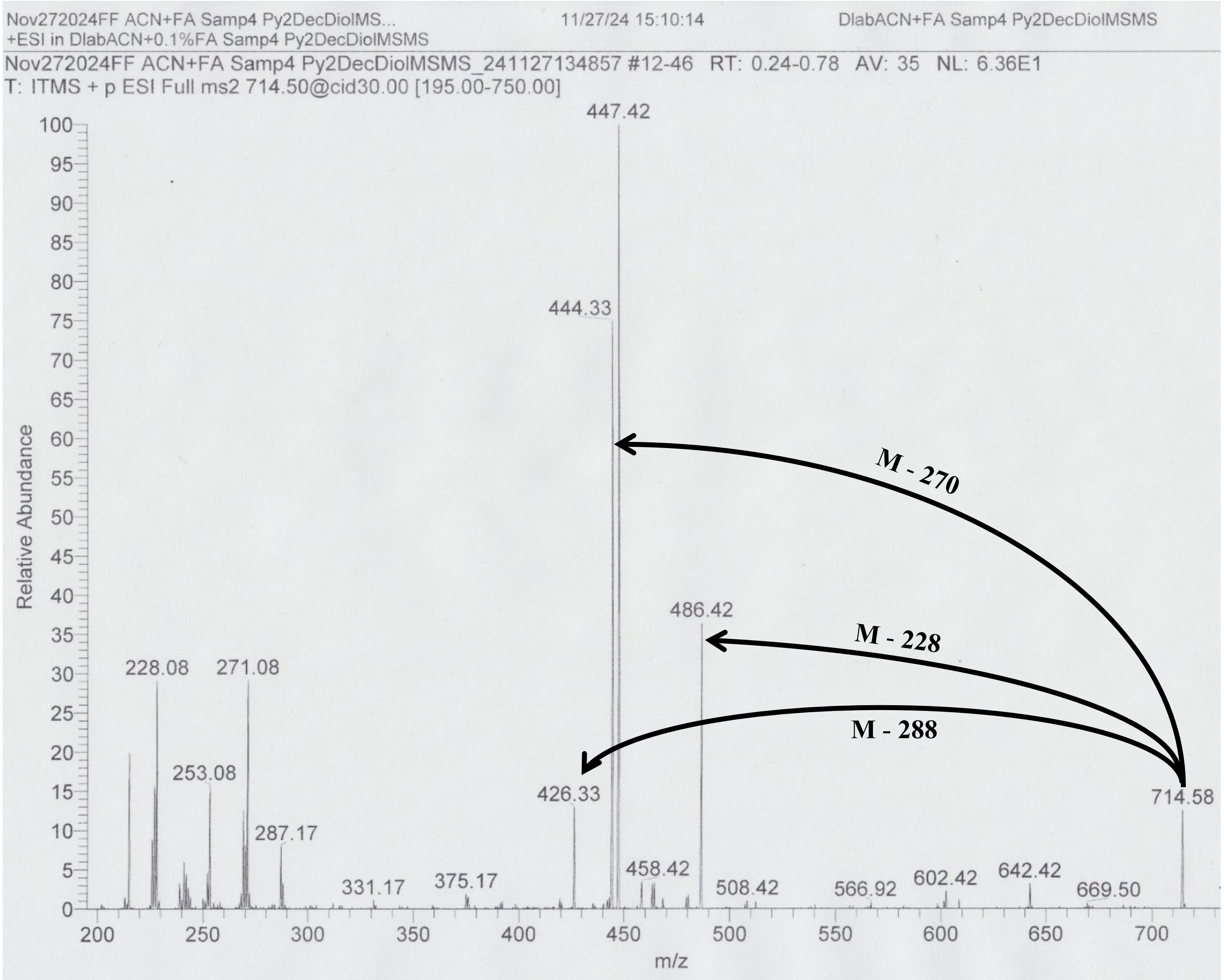

Figure S25. MS/MS Spectrum of Py_2-DecDiol with a selected precursor ion of 714.5 and common Py-PO fragments indicated.

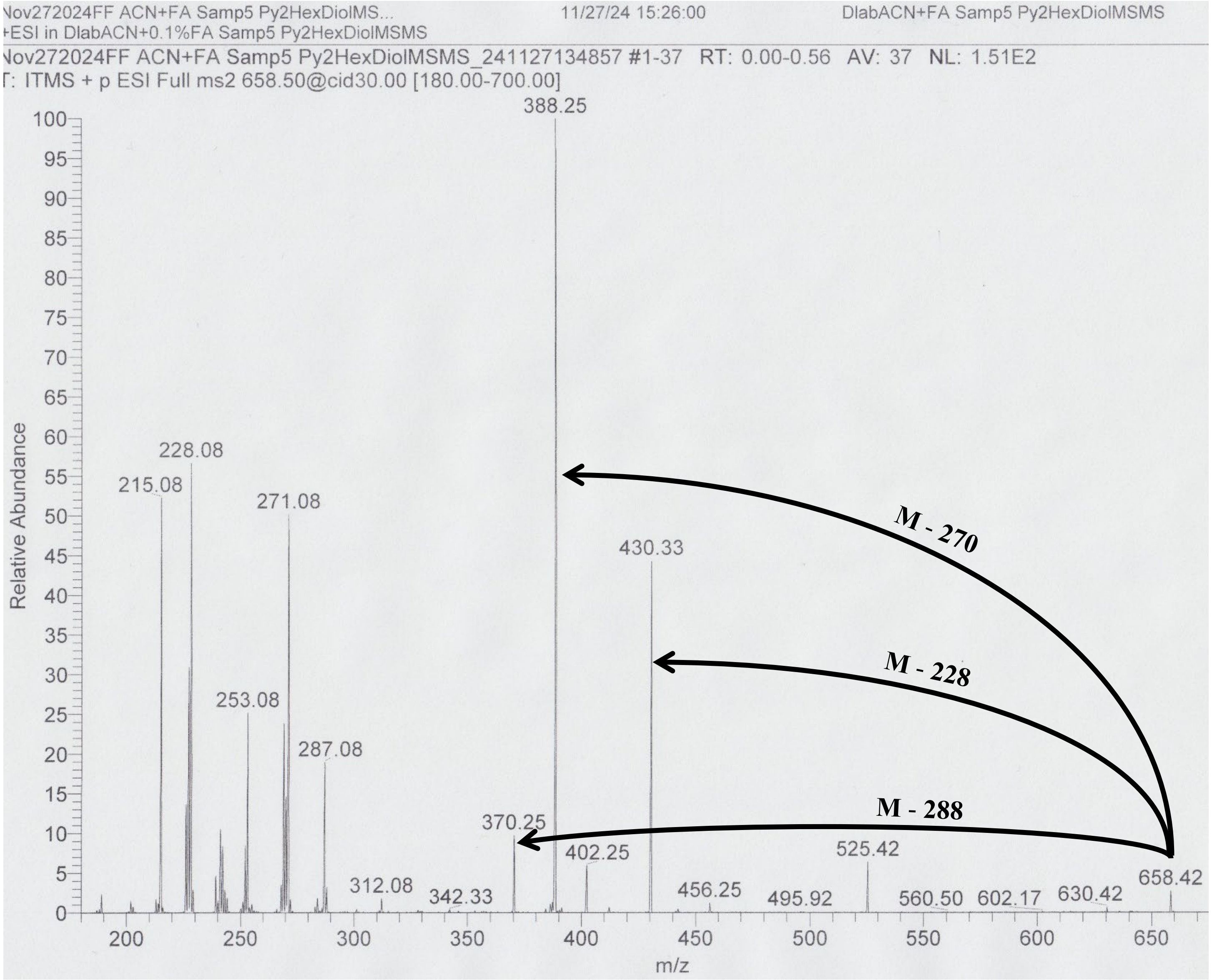

Figure S26. MS/MS Spectrum of Py_2-HexDiol with a selected precursor ion of 658.5 and common Py-PO fragments indicated.

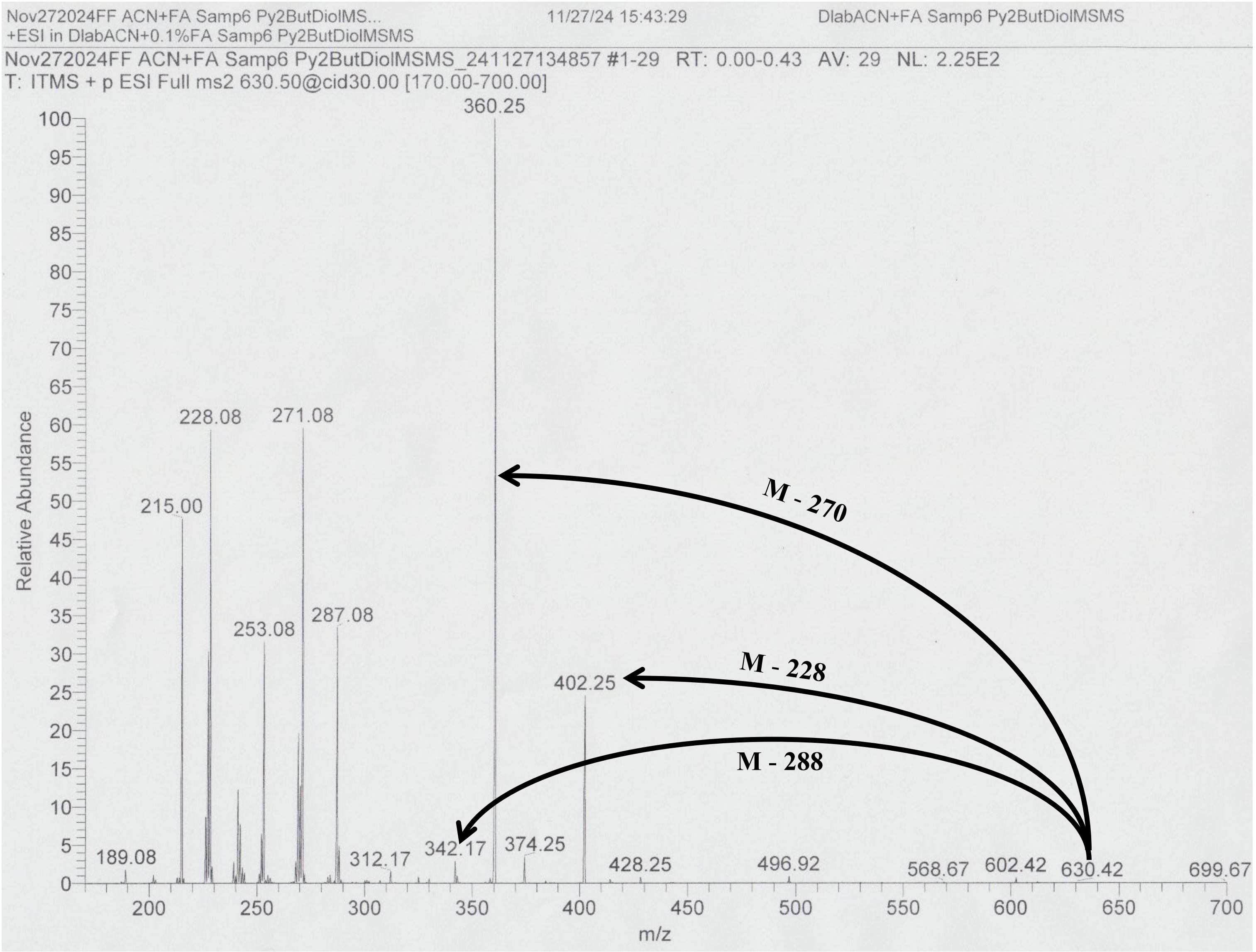

Figure S27. MS/MS Spectrum of Py_2-ButDiol with a selected precursor ion of 630.5 and common Py-PO fragments indicated.

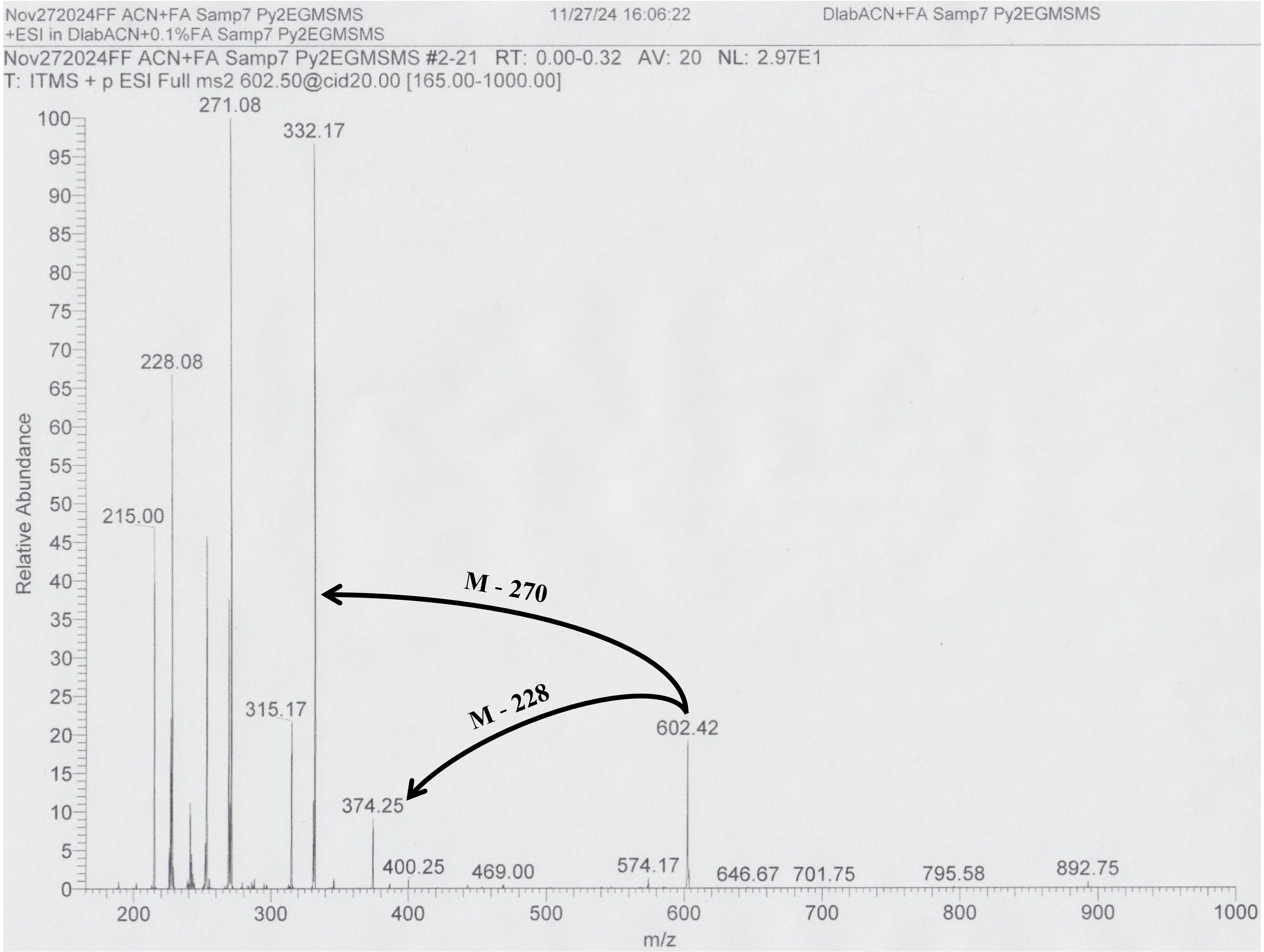

Figure S28. MS/MS Spectrum of Py_2-EG with a selected precursor ion of 602.5 and common Py-PO fragments indicated.

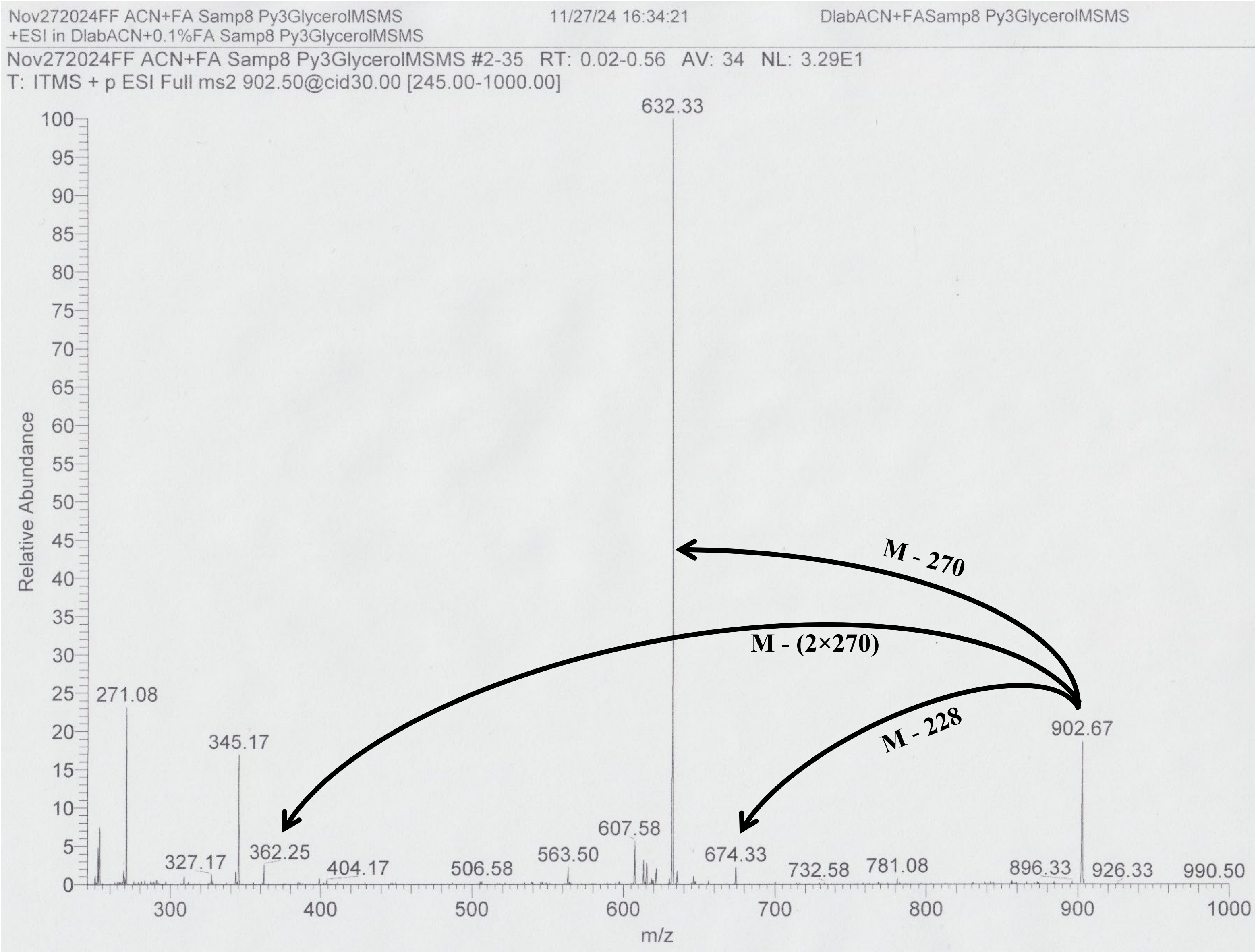

Figure S29. MS/MS Spectrum of Py_3-Glycerol with a selected precursor ion of 902.5 and common Py-PO fragments indicated.

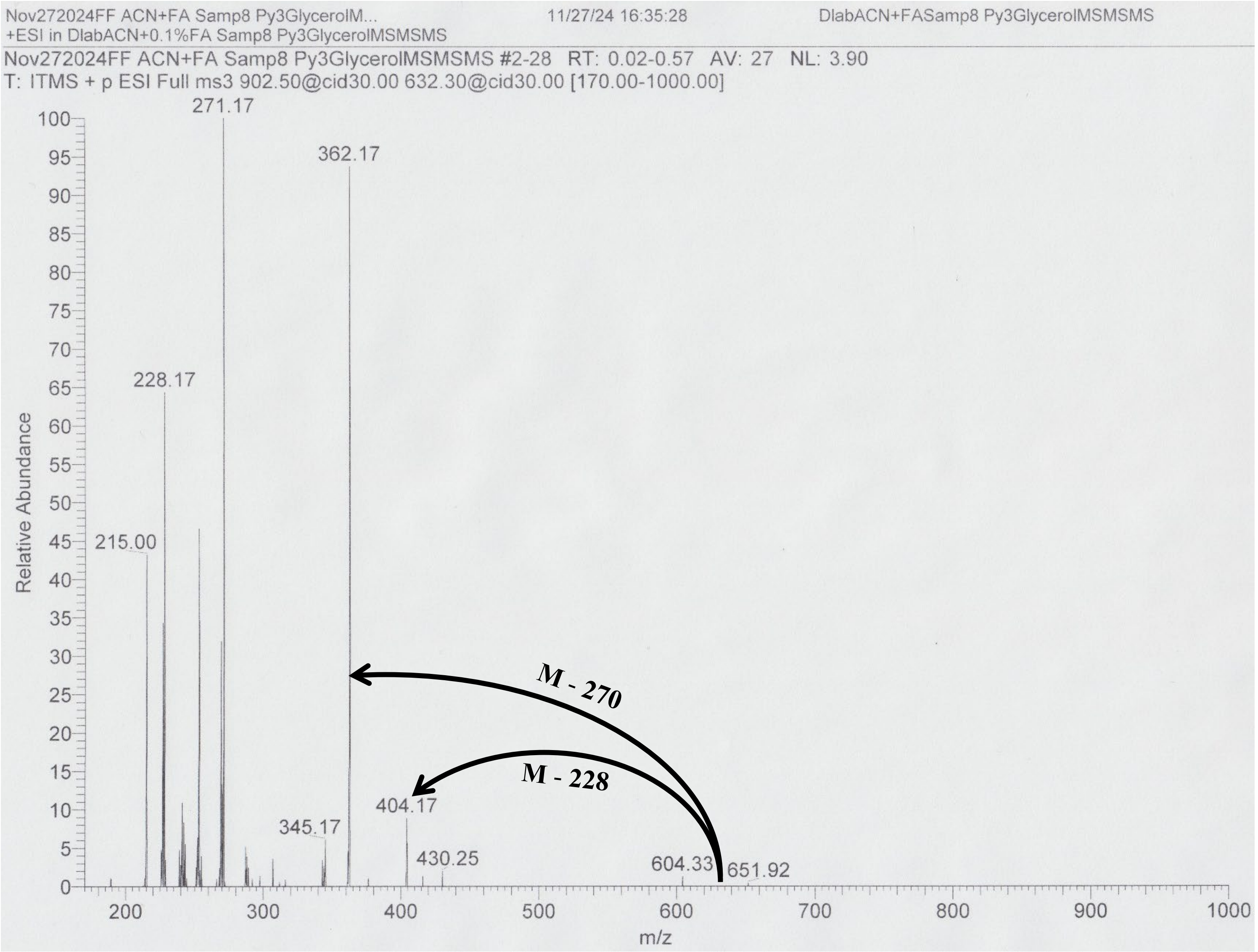

Figure S30. MS/MS/MS Spectrum of Py_3-Glycerol with selected precursor ions of 902.5 → 632.3 and common Py-PO fragments indicated.

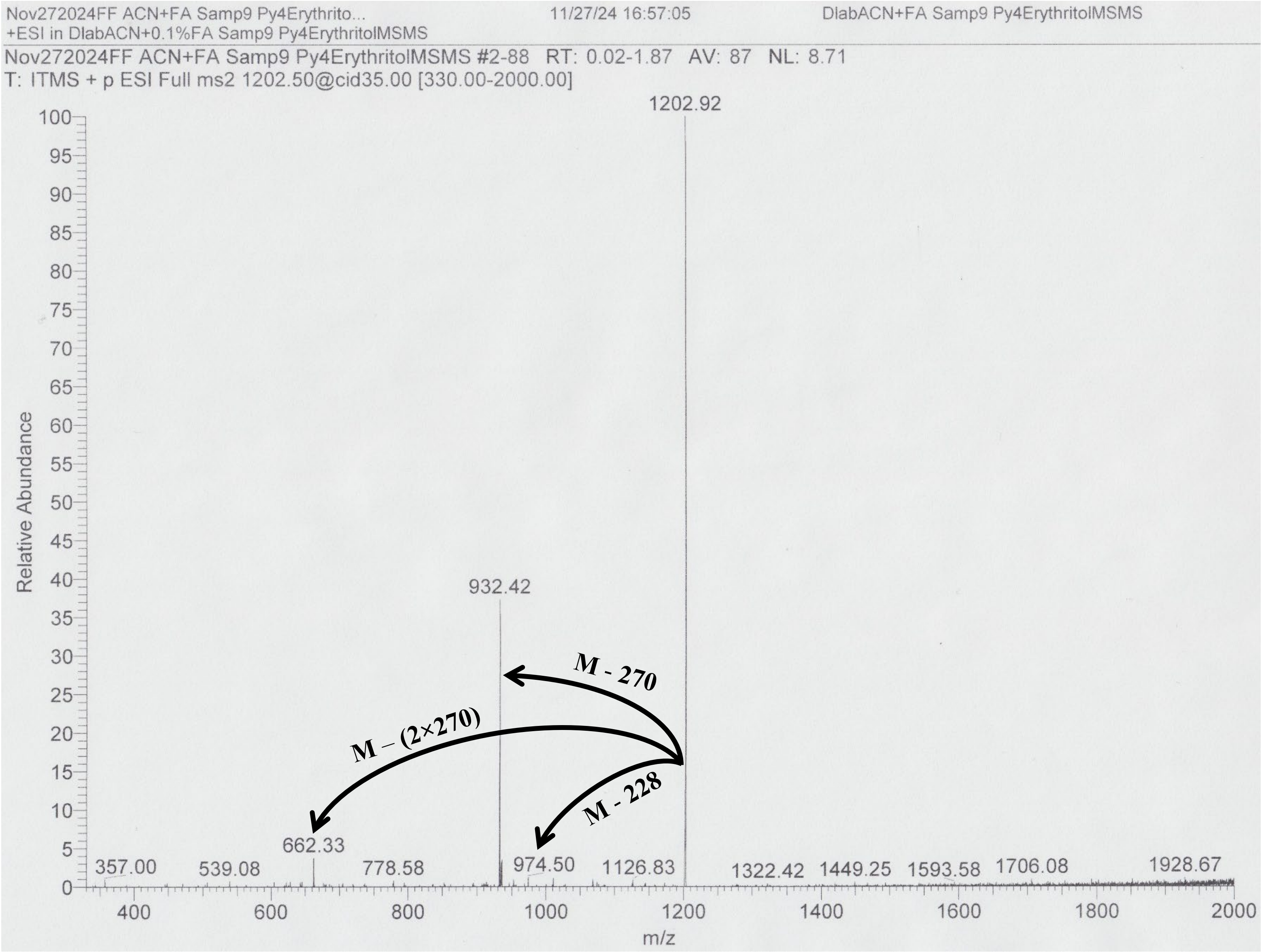

Figure S31. MS/MS Spectrum of Py_4-Erythritol with a selected precursor ion of 1202.5 and common Py-PO fragments indicated.

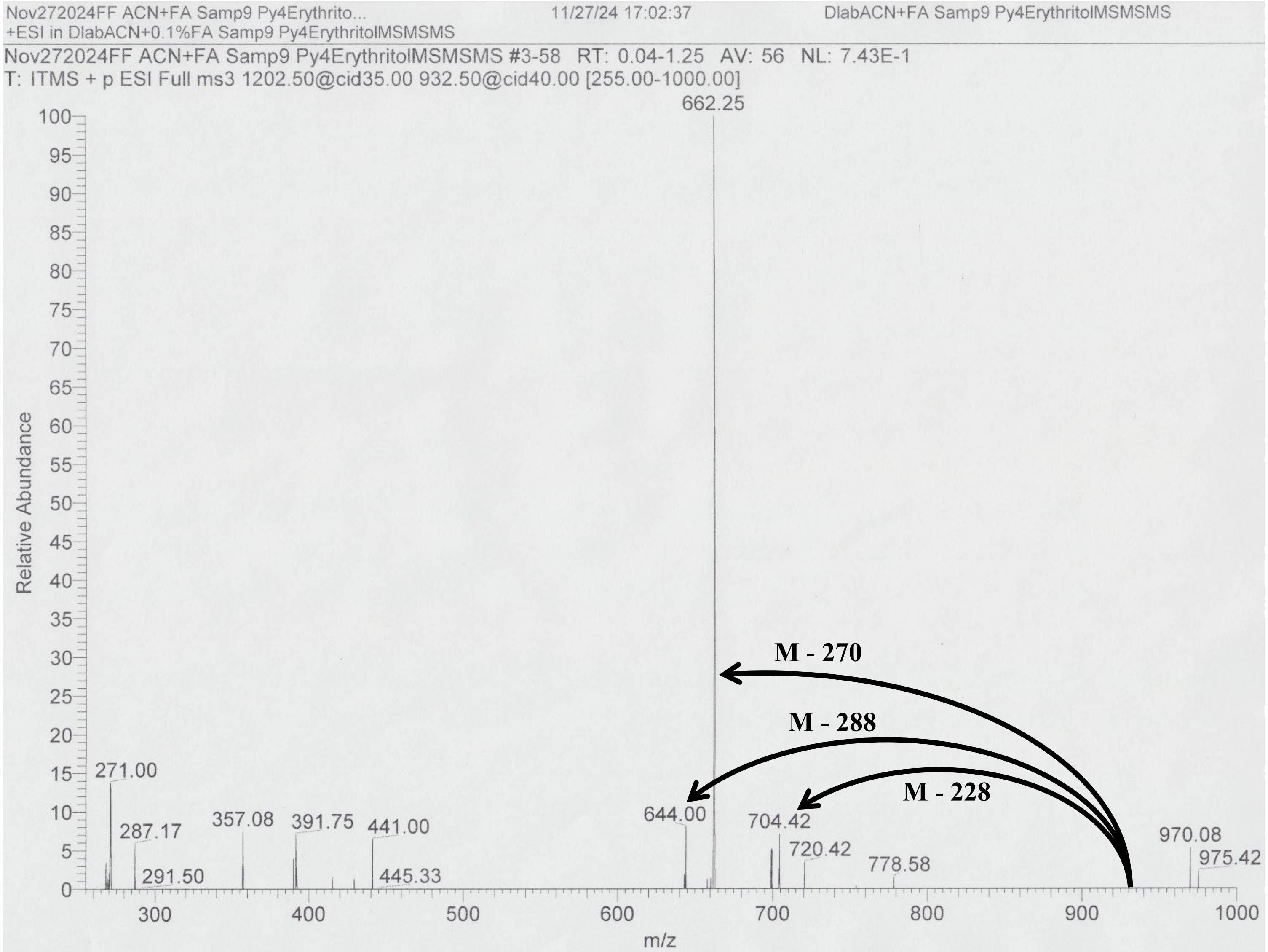

Figure S32. MS/MS/MS Spectrum of Py_4-Erythritol with selected precursor ions of 1202.5 → 932.5 and common Py-PO fragments indicated.

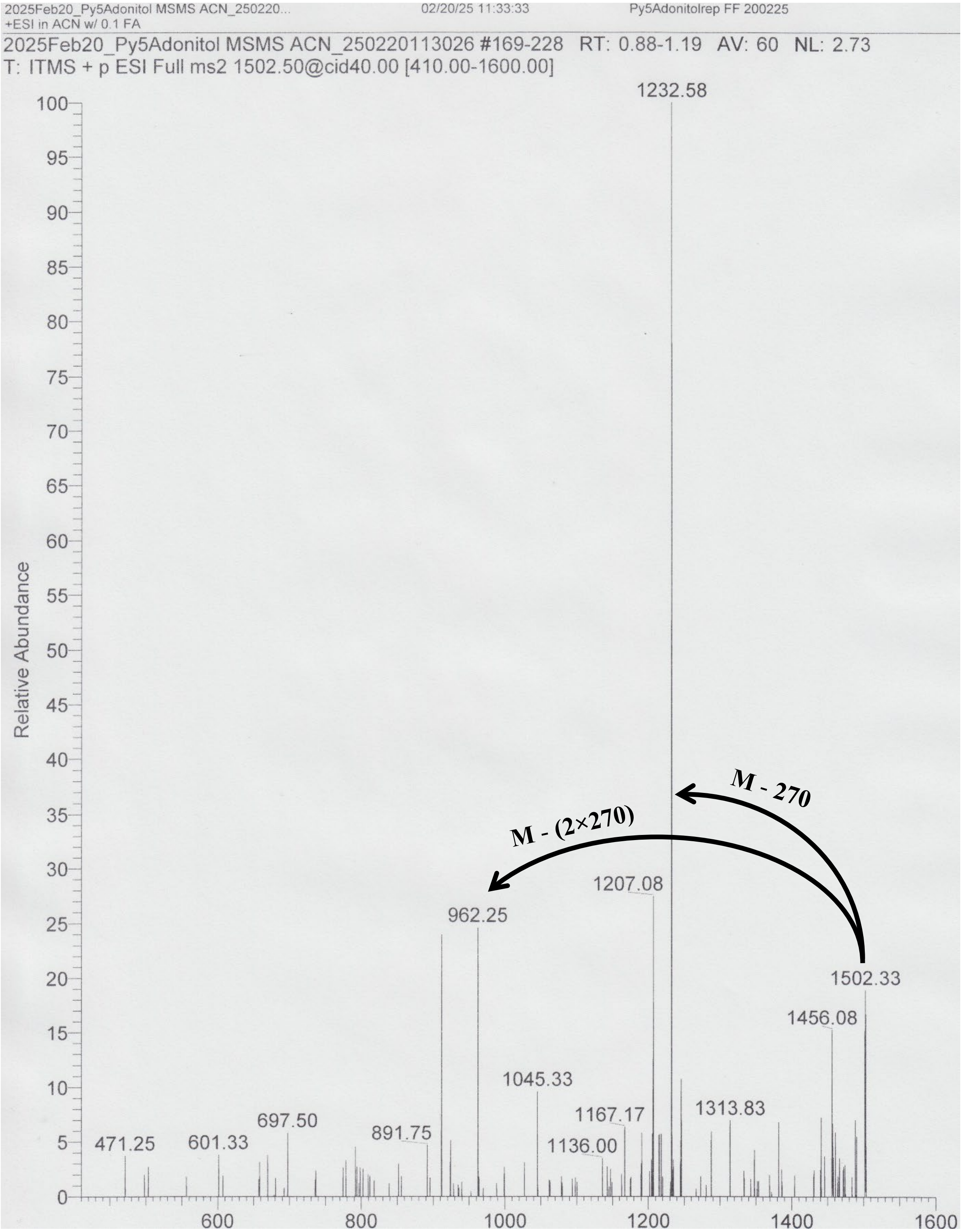

Figure S33. MS/MS Spectrum of Py_5-Adonitol with a selected precursor ion of 1502.5 and common Py-PO fragments indicated.

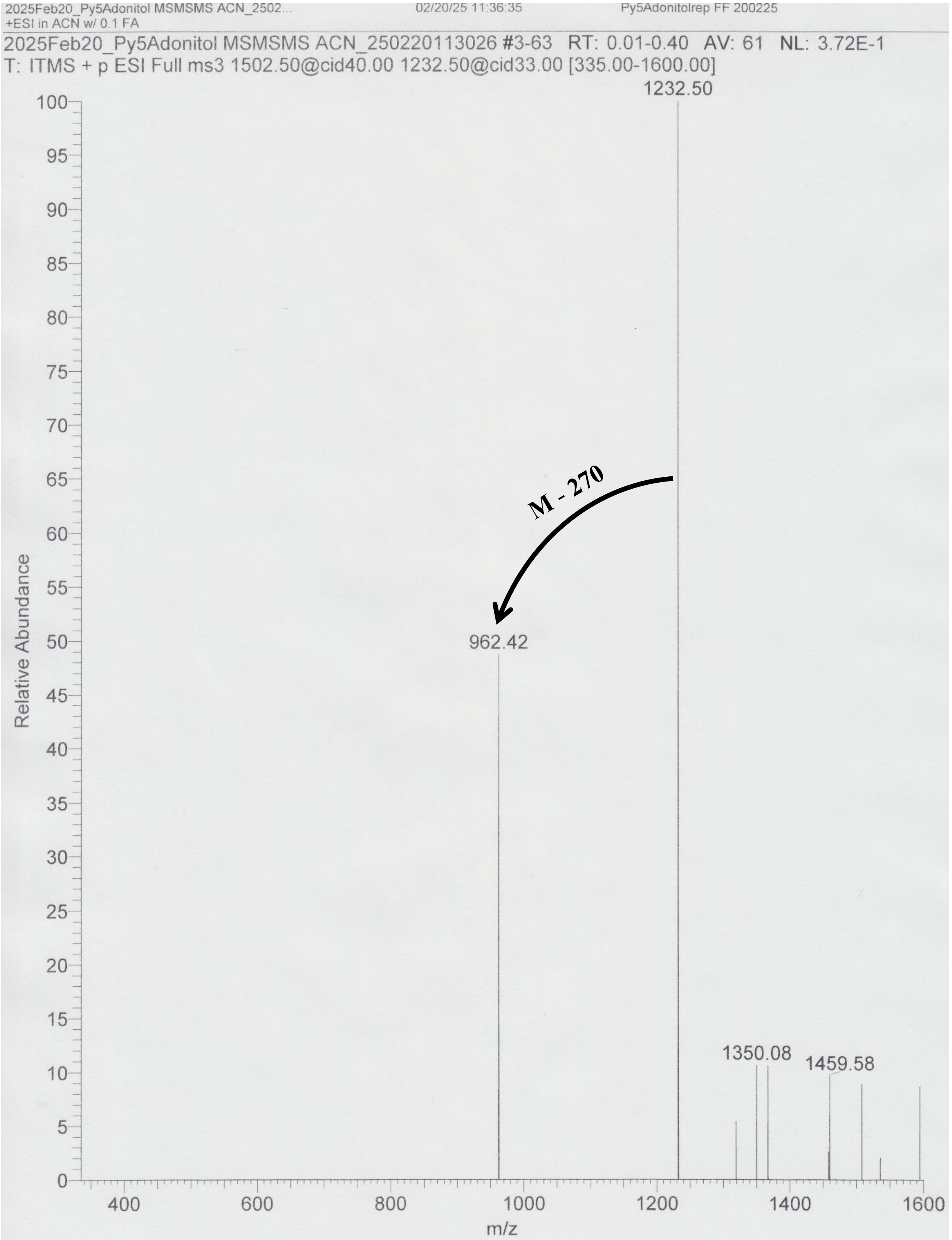

Figure S34. MS/MS/MS Spectrum of Py$_5$-Adonitol with selected precursor ions of 1502.5 → 1232.5 and common Py-PO fragments indicated.

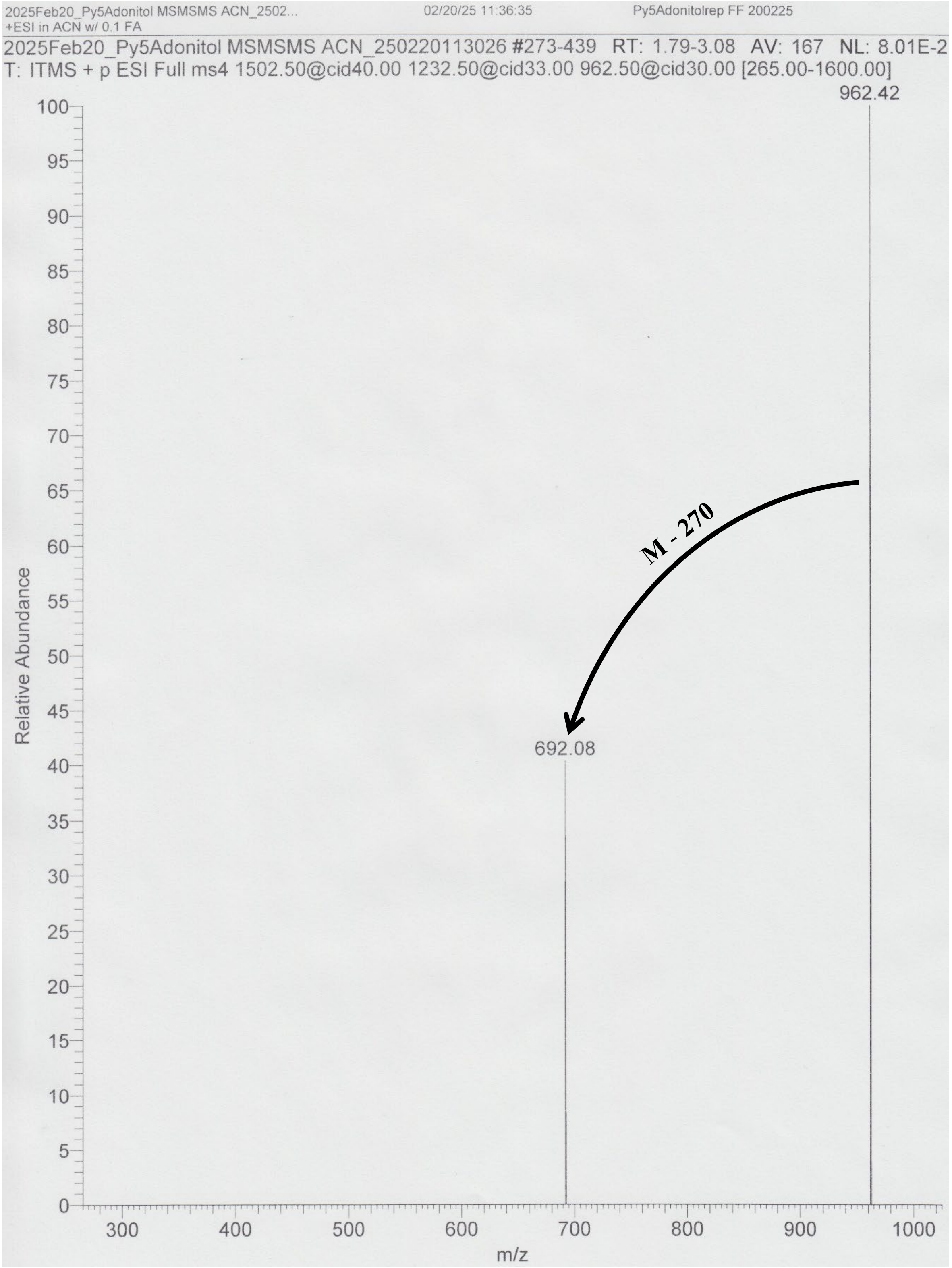

Figure S35. MS/MS/MS/MS Spectrum of Py$_5$-Adonitol with selected precursor ions of 1502.5 → 1232.5 → 962.5 and common Py-PO fragments indicated.

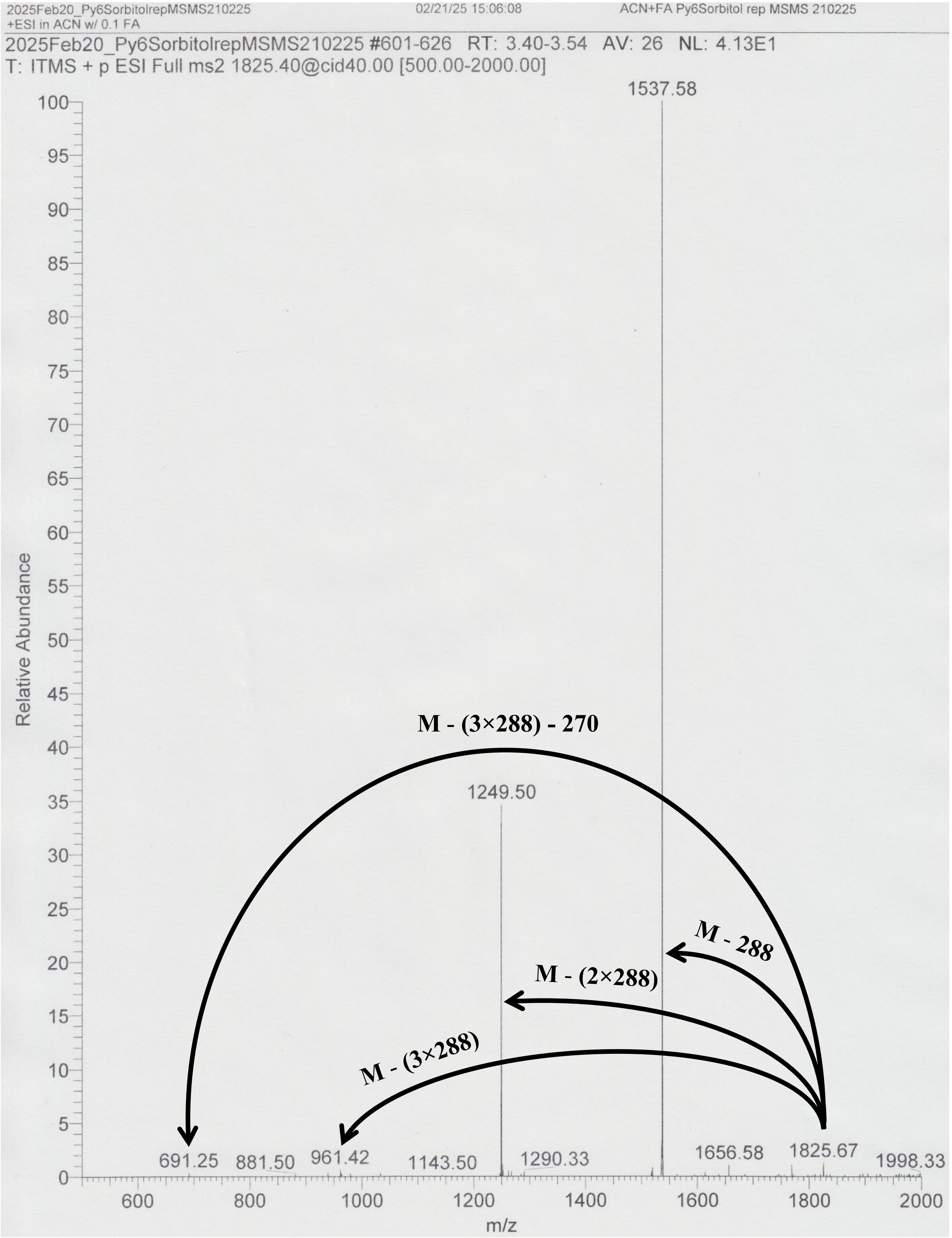

Figure S36. MS/MS Spectrum of Py_6-Sorbitol with a selected precursor ion of 1825.5 ($M+Na^+$ adduct) and common Py-PO fragments indicated.

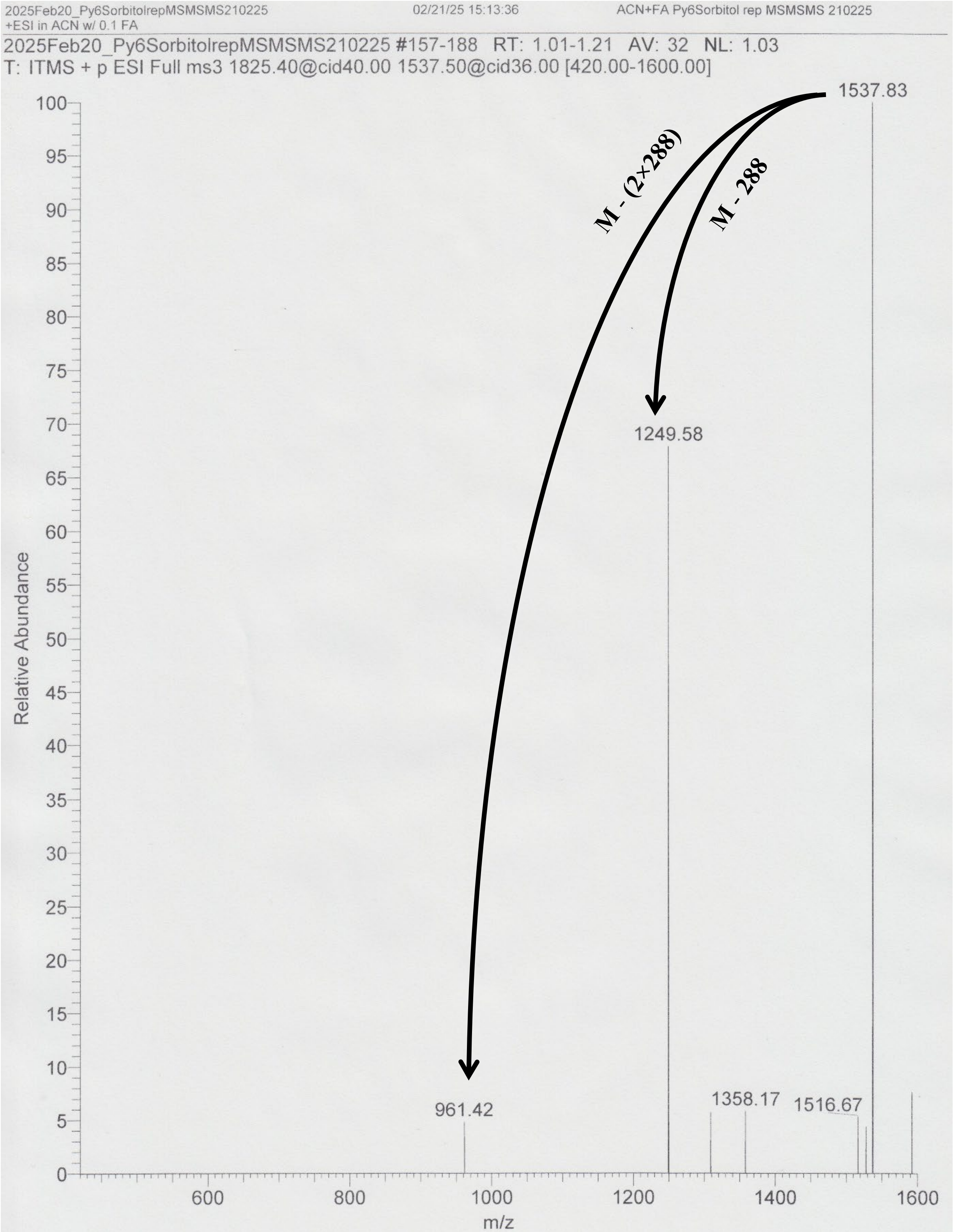

Figure S37. MS/MS/MS Spectrum of Py_6-Sorbitol with selected precursor ions of 1825.4 → 1537.5 and common Py-PO fragments indicated.

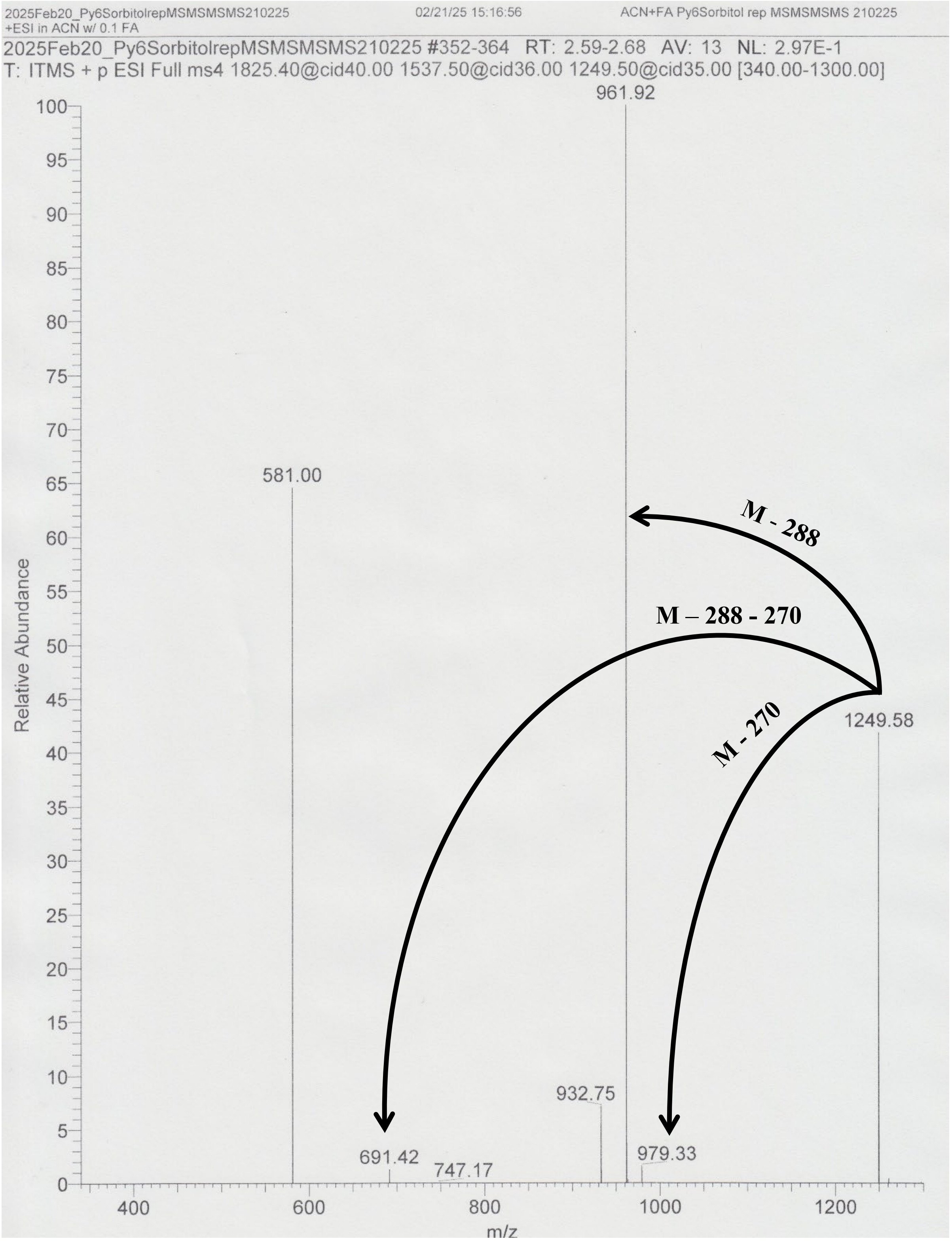

Figure S38. MS/MS/MS/MS Spectrum of Py_6-Sorbitol with selected precursor ions of 1825.4 → 1537.5 → 1249.5 and common Py-PO fragments indicated.

MS^n Discussion

The MS^n spectra showed similar features for all of the Py_2-DO and Py-PO samples, most notably the successive fragmentation of pyrene moieties from the Py-POs via α cleavage of an alkoxy radical in their ester linkages to yield the 270 MW fragments. There was also an appreciable amount of β-cleavage of the pyrene groups through either the McLafferty rearrangement to yield 228 MW fragments, or on the opposite side of the ester through loss of a carboxylic radical to yield the 288 MW fragments. It was noted that the Na-adducts tended to prefer the carboxylic cleavage to yield 288 MW fragments while most molecular (M^+) ions tended to favour the α-cleavage to yield 270 MW fragments. For the branched Py-POs with more than 2 pyrenes, cleavage appeared to occur multiple times and not just between MS^n to MS^{n+1} experiments but within a single MS/MS experiment (see Figures S29, S31, S33, and S36) showing successive fragmentation of the pyrene moieties from the parent ion. Further fragmentation with MS^n experiments (see Figures S30, S32, S34, S35, S37, and S38) showed that the pyrenes could be sequentially removed from the previous fragment which had lost one pyrene moiety to then lose a second pyrene, the fragment of which would then lose another pyrene upon fragmentation. These results along with the good agreement between the ^{1}H NMR and COSY spectra for the proposed Py_2-DO and Py-PO structures support that the polyols were fully labeled with 1-pyrenebutyric acid, a key requirement to calculate the $[Py]_{loc}$ values for the Py-PO samples.

D) SSF Spectra of the Py_2-DO and Py-PO samples in dioxane, DMF, and DMSO

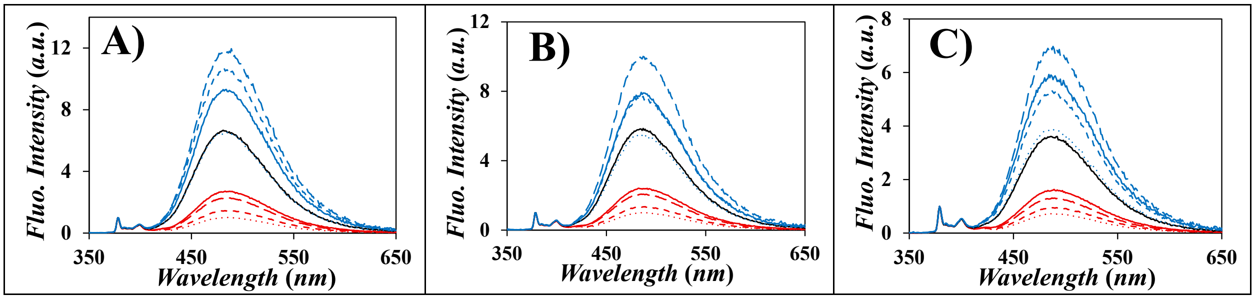

Figure S39. Plots of the steady-state fluorescence spectra of degassed solutions of the Py_2-DO and Py-PO samples with [*Py*] = 2.5 μM in A) dioxane, B) DMF, and C) DMSO. From top to bottom: (long dashes, blue) Py_5-Adonitol, (short dashes, blue) Py_4-Erythritol, (solid line, blue) Py_6-Sorbitol, (solid line, black) Py_2-EG, (dots, blue) Py_3-Glycerol, (solid line), red) Py_2-ButDiol, (long dashes, red) Py_2-HexDiol, (short dashes, red) Py_2-DecDiol, and (dots, red) Py_2-HexadecDiol.

E) Equations used for the global MFA of the monomer and excimer fluorescence decays

$$[M^*]_{(t)} = [Py_{diff}{}^*]_o \times \sum_{i=1}^{n} a_i \times \exp(-t/\tau_i) + [Py_{free}{}^*]_o \times \exp(-t/\tau_M) \tag{S1}$$

$$[E^*]_{(t)} = -[Py_{diffE0}{}^*]_o \sum_{i=1}^{n} a_i \frac{\frac{1}{\tau_i} - \frac{1}{\tau_M}}{\frac{1}{\tau_i} - \frac{1}{\tau_{E0}}} \exp(-t/\tau_i) + \left([E0^*]_o + [Py_{diffE0}{}^*]_o \sum_{i=1}^{n} a_i \frac{\frac{1}{\tau_i} - \frac{1}{\tau_M}}{\frac{1}{\tau_i} - \frac{1}{\tau_{E0}}} \right) \exp(-t/\tau_{E0})$$

$$-[Py_{diffD}{}^*]_o \sum_{i=1}^{n} a_i \frac{\frac{1}{\tau_i} - \frac{1}{\tau_M}}{\frac{1}{\tau_i} - \frac{1}{\tau_D}} \exp(-t/\tau_i) + \left([D^*]_o + [Py_{diffD}{}^*]_o \sum_{i=1}^{n} a_i \frac{\frac{1}{\tau_i} - \frac{1}{\tau_M}}{\frac{1}{\tau_i} - \frac{1}{\tau_D}} \right) \exp(-t/\tau_D) \tag{S2}$$

F) Equations used to calculate the molar fractions f_{diffE0}, f_{diffD}, f_{free}, f_{E0}, f_D, f_{diff}, and f_{agg}

$$f_{diffE0} = \frac{[Py_{diffE0}*]_o}{[Py_{diffE0}*]_o + [Py_{diffD}*]_o} \times \frac{1}{1 + \frac{[Py_{free}*]_o}{[Py_{diff}*]_o} + \frac{[E0*]_o + [D*]_o}{[Py_{diffE0}*]_o + [Py_{diffD}*]_o}} \quad \text{(S3)}$$

$$f_{diffD} = \frac{[Py_{diffD}*]_o}{[Py_{diffE0}*]_o + [Py_{diffD}*]_o} \times \frac{1}{1 + \frac{[Py_{free}*]_o}{[Py_{diff}*]_o} + \frac{[E0*]_o + [D*]_o}{[Py_{diffE0}*]_o + [Py_{diffD}*]_o}} \quad \text{(S4)}$$

$$f_{free} = (f_{diffE0} + f_{diffD}) \times \frac{[Py_{free}*]_o}{[Py_{diff}*]_o} \quad \text{(S5)}$$

$$f_{E0} = \frac{[E0*]_o}{[Py_{diffE0}*]_o + [Py_{diffD}*]_o} \times \frac{1}{1 + \frac{[Py_{free}*]_o}{[Py_{diff}*]_o} + \frac{[E0*]_o + [D*]_o}{[Py_{diffE0}*]_o + [Py_{diffD}*]_o}} \quad \text{(S6)}$$

$$f_{D} = \frac{[D*]_o}{[Py_{diffE0}*]_o + [Py_{diffD}*]_o} \times \frac{1}{1 + \frac{[Py_{free}*]_o}{[Py_{diff}*]_o} + \frac{[E0*]_o + [D*]_o}{[Py_{diffE0}*]_o + [Py_{diffD}*]_o}} \quad \text{(S7)}$$

$$f_{diff} = f_{diffE0} + f_{diffD} \quad \text{(S8)}$$

$$f_{agg} = f_{E0} + f_{D} \quad \text{(S9)}$$

G) Parameters obtained from the Model Free Analysis of the Py_2-DO and Py-PO samples

Table S1. Parameters retrieved from the MFA of the monomer fluorescence decays of the (Py_2-DO and Py-PO samples.

Solvent	Py-PO	a_1	τ_1 (ns)	a_2	τ_2 (ns)	a_3	τ_3 (ns)	f_{Mfree}	χ^2
**THF** τ_M = 217 ns	**Py_2-HexadecDiol**	0.83	16.90	0.16	40.62			0.01	1.01
	**Py_2-DecDiol**	0.91	12.33	0.09	52.79			0.00	1.14
	**Py_2-HexDiol**	0.95	8.36	0.05	46.64			0.00	1.00
	**Py_2-ButDiol**	0.97	6.30	0.02	48.19			0.01	1.03
	**Py_2-EG**	0.70	2.48	0.28	3.33	0.02	49.04	0.00	1.07
	**Py_3-Glycerol**	0.94	2.08	0.03	7.98	0.02	48.27	0.01	1.03
	**Py_4-Erythritol**	0.98	1.96	0.02	27.27			0.01	1.09
	**Py_5-Adonitol**	0.92	1.44	0.06	4.09	0.01	34.44	0.00	1.06
	**Py_6-Sorbitol**	0.97	1.30	0.02	15.61			0.01	1.01
**Dioxane** τ_M = 189 ns	**Py_2-HexadecDiol**	0.68	20.52	0.31	36.21			0.01	1.10
	**Py_2-DecDiol**	0.84	16.12	0.16	31.64			0.01	1.15
	**Py_2-HexDiol**	0.95	11.23	0.04	45.90			0.00	1.06
	**Py_2-ButDiol**	0.97	8.68	0.02	43.08			0.01	1.10
	**Py_2-EG**	0.92	3.66	0.06	6.28	0.01	58.69	0.00	1.07
	**Py_3-Glycerol**	0.43	2.42	0.54	3.86	0.02	35.24	0.01	0.97
	**Py_4-Erythritol**	0.73	2.22	0.25	3.78	0.01	34.26	0.00	1.04
	**Py_5-Adonitol**	0.67	1.67	0.31	3.07	0.01	25.22	0.00	1.10
	**Py_6-Sorbitol**	0.95	1.93	0.02	4.93	0.02	23.72	0.01	1.10
**DMF** τ_M = 181 ns	**Py_2-HexadecDiol**	0.91	21.43	0.08	51.26			0.00	1.00
	**Py_2-DecDiol**	0.72	14.61	0.27	24.20			0.01	1.02
	**Py_2-HexDiol**	0.96	10.56	0.04	44.59			0.00	1.02
	**Py_2-ButDiol**	0.97	8.30	0.02	41.88			0.01	1.06
	**Py_2-EG**	0.14	2.75	0.84	3.84	0.01	43.98	0.00	1.16
	**Py_3-Glycerol**	0.69	2.48	0.28	4.06	0.02	41.87	0.01	0.97
	**Py_4-Erythritol**	0.98	2.50	0.02	25.91			0.01	1.07
	**Py_5-Adonitol**	0.90	1.86	0.09	3.99	0.01	29.36	0.00	1.12
	**Py_6-Sorbitol**	0.97	1.82	0.02	12.22			0.01	1.06
**DMSO** τ_M = 145 ns	**Py_2-HexadecDiol**	0.53	22.22	0.47	30.84			0.00	1.10
	**Py_2-DecDiol**	0.80	18.36	0.20	28.14			0.01	1.03
	**Py_2-HexDiol**	0.95	13.67	0.05	34.26			0.00	1.09
	**Py_2-ButDiol**	0.96	11.18	0.02	28.21			0.01	1.10
	**Py_2-EG**	0.99	5.41	0.01	39.50			0.00	1.07
	**Py_3-Glycerol**	0.40	3.07	0.57	4.90	0.02	39.06	0.01	1.05
	**Py_4-Erythritol**	0.97	3.57	0.03	16.76			0.01	1.05
	**Py_5-Adonitol**	0.53	2.30	0.46	3.69	0.01	20.07	0.00	1.07
	**Py_6-Sorbitol**	0.45	1.88	0.52	3.34	0.01	22.93	0.01	1.08

Table S2. Parameters retrieved from the MFA of the excimer fluorescence decays of the Py_2-DO and Py-PO samples.

Solvent	Py-PO	$f_{EdiffE0}$	f_{EdiffD}	τ_{E0} (ns)	τ_D (ns)	f_{EE0}	f_{ED}	χ^2
THF	Py_2-HexadecDiol	0.47	0.53	44.36	60.55	0.00	0.00	1.01
	Py_2-DecDiol	0.43	0.57	45.46	56.74	0.00	0.00	1.02
	Py_2-HexDiol	0.30	0.69	33.60	60.53	0.00	0.01	1.02
	Py_2-ButDiol	0.52	0.46	44.72	66.03	0.00	0.02	1.06
	Py_2-EG	0.53	0.45	43.01	64.75	0.00	0.02	1.16
	Py_3-Glycerol	0.64	0.26	47.57	60.42	0.00	0.10	0.97
	Py_4-Erythritol	0.76		53.48		0.24		1.07
	Py_5-Adonitol	0.41	0.50	47.46	56.07	0.00	0.09	1.12
	Py_6-Sorbitol	0.88		52.20		0.12		1.06
Dioxane	Py_2-HexadecDiol	0.43	0.56	50.81	54.68	0.00	0.00	1.10
	Py_2-DecDiol	0.44	0.55	45.35	56.56	0.00	0.00	1.03
	Py_2-HexDiol	0.29	0.70	34.28	58.55	0.01	0.00	1.09
	Py_2-ButDiol	0.49	0.49	42.67	63.69	0.00	0.02	1.10
	Py_2-EG	0.49	0.45	39.86	62.77	0.00	0.05	1.07
	Py_3-Glycerol	0.51	0.39	43.95	60.00	0.00	0.10	1.05
	Py_4-Erythritol	0.49	0.40	44.30	59.23	0.00	0.11	1.05
	Py_5-Adonitol	0.60	0.27	45.78	61.07	0.00	0.13	1.07
	Py_6-Sorbitol	0.40	0.37	45.31	55.53	0.00	0.23	1.08
DMF	Py_2-HexadecDiol	1.00		50.53		0.00		1.00
	Py_2-DecDiol	0.57	0.43	44.51	57.25	0.00	0.00	1.02
	Py_2-HexDiol	0.58	0.42	40.82	61.51	0.00	0.00	1.02
	Py_2-ButDiol	0.50	0.48	43.10	60.36	0.00	0.01	1.06
	Py_2-EG	0.65	0.32	42.51	61.88	0.00	0.02	1.16
	Py_3-Glycerol	0.61	0.33	43.71	56.85	0.00	0.06	0.97
	Py_4-Erythritol	0.54	0.34	43.91	55.50	0.00	0.12	1.07
	Py_5-Adonitol	0.68	0.24	46.64	57.42	0.00	0.08	1.12
	Py_6-Sorbitol	0.51	0.40	44.36	55.19	0.00	0.09	1.06
DMSO	Py_2-HexadecDiol	0.98		47.63		0.02		1.10
	Py_2-DecDiol	0.98		47.15		0.02		1.03
	Py_2-HexDiol	0.56	0.42	38.41	56.08	0.00	0.02	1.09
	Py_2-ButDiol	0.33	0.64	37.66	53.17	0.00	0.03	1.10
	Py_2-EG	0.11	0.83	21.28	47.52	0.00	0.06	1.07
	Py_3-Glycerol	0.18	0.74	33.16	47.52	0.00	0.08	1.05
	Py_4-Erythritol	0.47	0.43	38.71	51.97	0.00	0.09	1.05
	Py_5-Adonitol	0.58	0.30	43.62	52.72	0.05	0.08	1.07
	Py_6-Sorbitol	0.63	0.27	42.06	54.50	0.00	0.10	1.08

Table S3. Parameters obtained from the MFA of the monomer and excimer fluorescence decays of the Py$_2$-DO and Py-PO samples.

Solvent	Py-PO	f_{diffE0}	f_{diffD}	f_{E0}	f_{D}	f_{diff}	f_{agg}	f_{free}
**THF**	**Py$_2$-HexadecDiol**	0.46	0.52	0.00	0.00	0.98	0.00	0.01
	**Py$_2$-DecDiol**	0.43	0.57	0.00	0.00	1.00	0.00	0.00
	**Py$_2$-HexDiol**	0.30	0.69	0.00	0.01	0.99	0.01	0.00
	**Py$_2$-ButDiol**	0.52	0.46	0.00	0.02	0.97	0.02	0.01
	**Py$_2$-EG**	0.53	0.45	0.00	0.02	0.97	0.02	0.00
	**Py$_3$-Glycerol**	0.63	0.26	0.00	0.10	0.89	0.10	0.01
	**Py$_4$-Erythritol**	0.76		0.24		0.76	0.24	0.00
	**Py$_5$-Adonitol**	0.41	0.50	0.00	0.09	0.90	0.09	0.00
	**Py$_6$-Sorbitol**	0.87		0.12		0.87	0.12	0.01
**Dioxane**	**Py$_2$-HexadecDiol**	0.43	0.56	0.00	0.00	0.99	0.00	0.01
	**Py$_2$-DecDiol**	0.44	0.55	0.00	0.00	0.99	0.00	0.01
	**Py$_2$-HexDiol**	0.29	0.69	0.01	0.00	0.99	0.01	0.00
	**Py$_2$-ButDiol**	0.48	0.49	0.00	0.02	0.97	0.02	0.01
	**Py$_2$-EG**	0.49	0.45	0.00	0.05	0.94	0.05	0.00
	**Py$_3$-Glycerol**	0.51	0.39	0.00	0.10	0.90	0.10	0.01
	**Py$_4$-Erythritol**	0.49	0.40	0.00	0.11	0.89	0.11	0.00
	**Py$_5$-Adonitol**	0.60	0.27	0.00	0.13	0.87	0.13	0.00
	**Py$_6$-Sorbitol**	0.39	0.37	0.00	0.23	0.76	0.23	0.01
**DMF**	**Py$_2$-HexadecDiol**	0.99		0.00		0.99	0.00	0.00
	**Py$_2$-DecDiol**	0.56	0.43	0.00	0.00	0.99	0.00	0.01
	**Py$_2$-HexDiol**	0.57	0.42	0.00	0.00	0.99	0.00	0.00
	**Py$_2$-ButDiol**	0.50	0.48	0.00	0.01	0.97	0.01	0.01
	**Py$_2$-EG**	0.65	0.32	0.00	0.02	0.97	0.02	0.00
	**Py$_3$-Glycerol**	0.61	0.33	0.00	0.06	0.93	0.06	0.01
	**Py$_4$-Erythritol**	0.54	0.34	0.00	0.11	0.88	0.11	0.01
	**Py$_5$-Adonitol**	0.68	0.24	0.00	0.08	0.91	0.08	0.00
	**Py$_6$-Sorbitol**	0.50	0.40	0.00	0.09	0.90	0.09	0.01
**DMSO**	**Py$_2$-HexadecDiol**	0.98		0.02		0.98	0.02	0.00
	**Py$_2$-DecDiol**	0.98		0.02		0.98	0.02	0.01
	**Py$_2$-HexDiol**	0.55	0.42	0.00	0.02	0.97	0.02	0.00
	**Py$_2$-ButDiol**	0.33	0.63	0.00	0.03	0.96	0.03	0.01
	**Py$_2$-EG**	0.11	0.83	0.00	0.06	0.93	0.06	0.00
	**Py$_3$-Glycerol**	0.18	0.73	0.00	0.08	0.91	0.08	0.01
	**Py$_4$-Erythritol**	0.47	0.43	0.00	0.09	0.90	0.10	0.01
	**Py$_5$-Adonitol**	0.57	0.30	0.05	0.08	0.87	0.12	0.00
	**Py$_6$-Sorbitol**	0.63	0.26	0.00	0.10	0.89	0.10	0.01

H) Molar Fractions of the Py_2-DO and Py-PO samples obtained from the Model Free Analysis

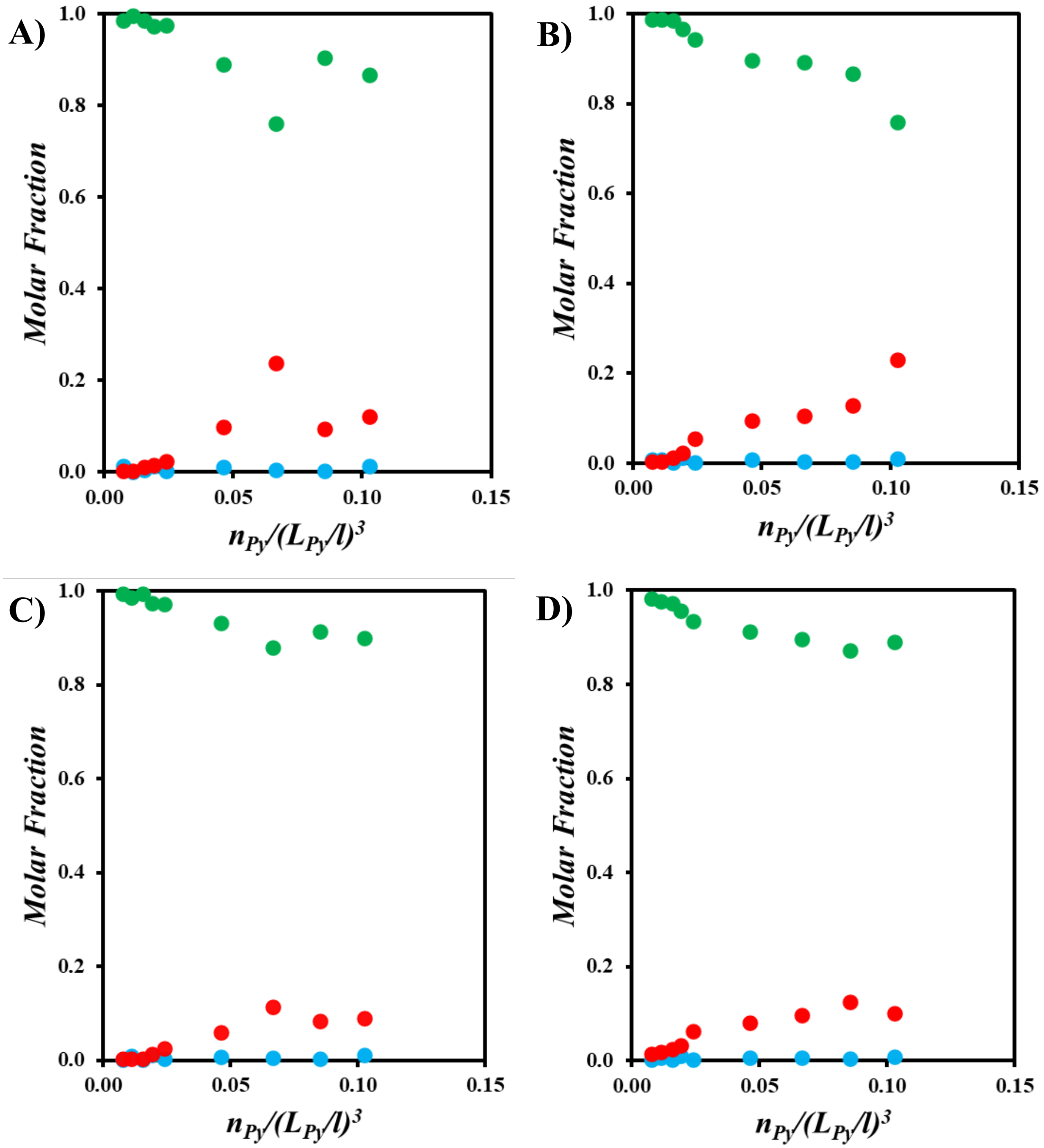

Figure S40. Molar fractions f_{diff} (green), f_{agg} (red), and f_{free} (blue) vs. $n_{Py}/(L_{Py}/l)^3$ obtained by the global MFA of the monomer and excimer fluorescence decays of the Py_2-DO and Py-PO samples in A) THF, B) dioxane, C) DMF, and D) DMSO.

I) Determination of k_{diff} for PyBE in Dioxane

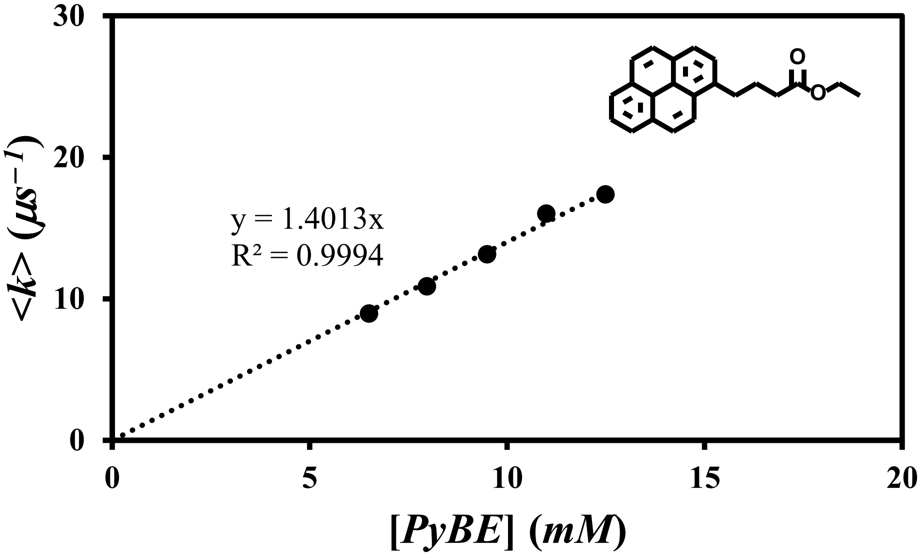

Figure S41. Plot of <*k*>-*vs*.-[*Py*] for degassed solutions of the model compound PyBE in dioxane.
